# Total synthesis of atropurpuran

**DOI:** 10.1038/ncomms12183

**Published:** 2016-07-08

**Authors:** Jing Gong, Huan Chen, Xiao-Yu Liu, Zhi-Xiu Wang, Wei Nie, Yong Qin

**Affiliations:** 1 Key Laboratory of Drug Targeting and Drug Delivery Systems of the Ministry of Education, Department of Chemistry of Medicinal Natural Products, West China School of Pharmacy, and State Key Laboratory of Biotherapy, Sichuan University, No. 17, Section 3, Renmin Nan Road, Chengdu 610041, China

## Abstract

Due to their architectural intricacy and biological significance, the synthesis of polycyclic diterpenes and their biogenetically related alkaloids have been the subject of considerable interest over the last few decades, with progress including the impressive synthesis of several elusive targets. Despite tremendous efforts, conquering the unique structural types of this large natural product family remains a long-term challenge. The arcutane diterpenes and related alkaloids, bearing a congested tetracyclo[5.3.3.0^4,9^.0^4,12^]tridecane unit, are included in these unsolved enigmas. Here we report a concise approach to the construction of the core structure of these molecules and the first total synthesis of (±)-atropurpuran. Pivotal features of the synthesis include an oxidative dearomatization/intramolecular Diels-Alder cycloaddition cascade, sequential aldol and ketyl-olefin cyclizations to assemble the highly caged framework, and a chemoselective and stereoselective reduction to install the requisite allylic hydroxyl group in the target molecule.

## Abstract (web summary)

Polycyclic and caged molecules are common in nature but present challenging targets for synthesis. Here, the authors report a route to caged diterpene cores and apply to the total synthesis of atropurpuran, with oxidative dearomatization/intramolecular Diels-Alder cascade, aldol and ketyl-olefin cyclizations as key steps.

## Graphical abstract

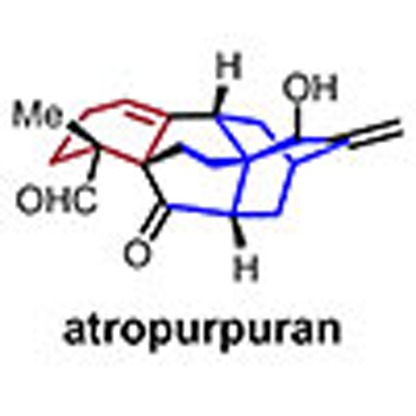

Three-dimensionally complex and highly cage-like molecules represent some of the amazing natural products that provide both enormous benefits and new challenges to mankind^1^. For instance, the polycyclic diterpenes and their alkaloidal congeners, both featuring difficult chemical structures and possible medicinal applications, fall into such a category^2^,^3^,^4^. From a biogenetic viewpoint, the formation of structurally diverse diterpenes relies on cyclization and oxidation stages, starting from geranylgeranyl diphosphate^5^,^6^, and the introduction of nitrogen atoms into the well-established diterpene skeletons affords the diterpenoid alkaloids^7^,^8^. While the genera of *Aconitum* and *Delphinium* provided rich sources for various types of these pseudo-alkaloids^7^, only a few of their parent diterpene molecules were isolated from nature. As illustrated in Fig. 1a, identification of the atisane-, hetidane- and arcutane-type diterpenes (**1**–**3**, Fig. 1a) belonged to such exceptional cases^9^,^10^,^11^ in which the corresponding natural alkaloids (**4**–**6**) were isolated^12^,^13^,^14^,^15^,^16^. All these structural types were biosynthetically derived from atisane diterpenes and are closely related to each other. Specifically, based on the atisane/atisine cores, C14–C20 bond construction afforded hetidanes/hetidines^17^ and further bond rearrangement (C10–C20→C5–C20) gave rise to the arcutane/arcutine frameworks^17^,^18^.

Over the past decade, the synthetic community has made great efforts in pursuit of the total synthesis of atisane diterpenes and their related diterpenoid alkaloids with some notable successes^2^,^3^,^4^,^19^,^20^,^21^,^22^,^23^,^24^,^25^,^26^,^27^,^28^. The teams of Carreira and Liu reported creative solutions for the total synthesis of a 3,4-seco atisane diterpene (crotogoudin, **7**, Fig. 1b)^23^,^26^, respectively. Baran and co-workers disclosed a unified access to methyl atisenoate (**1**), isoatisine (**8**) and the hetidine skeleton from a common intermediate^24^. Additionally, the Fukuyama group contributed to the first total synthesis of a denudatine alkaloid (lepenine, **9**)^25^, while Gin *et al* realized a refined synthesis of the hetisine-type alkaloid nominine (**10**)^19^. Very recently, an elegant approach to weisaconitine D (**11**) and liljestrandinine (**12**) was developed by Sarpong and co-workers employing a network analysis strategy^27^, which has enabled this area to reach new technical heights. Despite these advances, the total synthesis of the unique arcutane/arcutine group (for example, atropurpuran **3** and arcutinine **6**) has been impeded by the complexity of the cage-like tetracyclo[5.3.3.0^4,9^.0^4,12^]tridecane unit (blue bonds shown in Fig. 1a). Aside from the bridged ring systems, the challenging task was further complicated by the presence of multiple stereogenic centres embedded in the molecules, including two all-carbon bridgehead quaternary centres (C5 and C8). These natural products have been the focus of many synthetic studies^29^,^30^,^31^,^32^ due not only to their alluring structures, but also because their biological functions are unknown. Here, we describe our recent endeavours that led to the first total synthesis of (±)-atropurpuran.

## Results

### Retrosynthetic analysis

**Figure 2:**
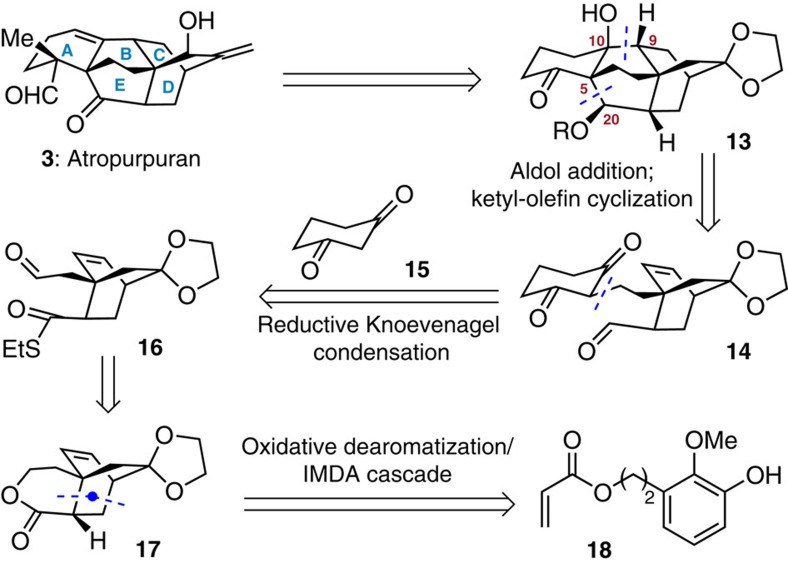
Retrosynthetic analysis of atropurpuran (3). The key disconnections involve an aldol reaction and a ketyl-olefin cyclization to construct rings E and B, respectively, at a late stage.

Our retrosynthetic analysis is outlined in Fig. 2. The target molecule atropurpuran (**3**) could be accessed through functional group manipulations from an advanced intermediate **13**. Central to the synthesis of **3** would be the assembly of its core structure (that is, **13**), which can be realized from keto aldehyde **14** via a sequence of aldol addition and ketyl-olefin cyclization^33^. Construction of the C5–C20 bond before the C9–C10 bond would be critical for delivering the right stereochemical outcome at C9. We speculated that the precursor **14** could in turn be obtained by a reductive Knoevenagel condensation^34^ between 1,3-cyclohexanedione (**15**) and aldehyde **16**. Generation of tricycle **17** (the precursor of **16**) would rely on an oxidative dearomatization/intramolecular Diels-Alder cycloaddition (IMDA) cascade reaction from the olefinic phenol **18**, which has been proven to be efficient for stereoselective synthesis of bicyclo[2.2.2]octane systems^35^.

### Synthesis of the pentacyclic core structure

**Figure 3:**
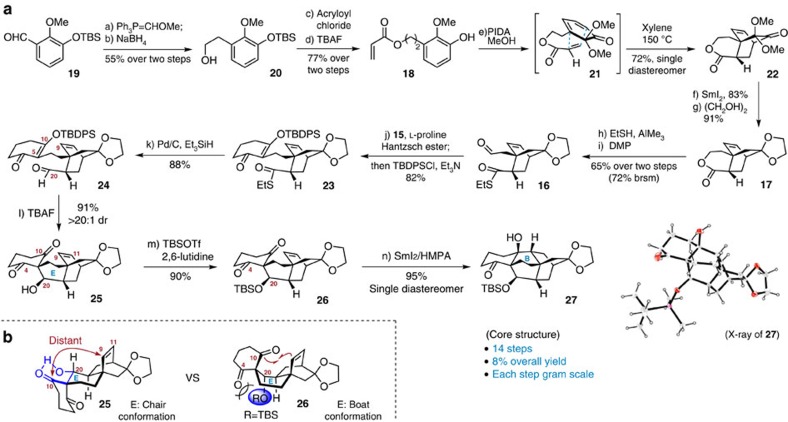
Access to the core structure for both arcutane diterpenes and arcutine alkaloids. (**a**) Conditions: (a) Ph_3_PCH_2_OMeCl, *t*-BuOK, THF, 0 °C; then TMSCl, NaI, MeCN, −18 °C; (b) NaBH_4_, MeOH, 0 °C, 55% over two steps; (c) acryloyl chloride, Et_3_N, CH_2_Cl_2_, rt; (d) TBAF, THF, rt, 77% over two steps; (e) PIDA, MeOH, 0 °C; then xylene, BHT, 150 °C; 72%; (f) SmI_2_, THF/MeOH (20:1), rt, 83%; (g) ethylene glycol, TMSCl, CH_2_Cl_2_, rt, 91%; (h) EtSH, AlMe_3_, CH_2_Cl_2_, 0 °C to rt; (i) DMP, CH_2_Cl_2_, rt, 65% over two steps (72% brsm); (j) 1,3-cyclohexanedione (**15**), Hantzsch ester, L-proline, CH_2_Cl_2_; then TBDPSCl, Et_3_N, CH_2_Cl_2_, rt, 82%; (k) Pd/C, Et_3_SiH, CH_2_Cl_2_, rt, 88%; (l) TBAF, THF, rt, 91% (>20:1 dr); (m) TBSOTf, 2,6-lutidine, CH_2_Cl_2_, –40 °C, 90%; (n) SmI_2_, HMPA, THF/*t*-BuOH (20:1), rt, 95%. (**b**) Comparison of the conformational preferences of intermediates **25** and **26**. BHT, 2,6-bis(1,1-dimethylethyl)-4-methylphenol; brsm, based on recovered starting material; HMPA, hexamethylphosphoramide; PIDA, iodobenzene diacetate; TBAF, tetra-*n*-butylammonium fluoride; TBDPSCl, *tert*-butyl(chloro)diphenylsilane; TBS, *tert*-butyldimethylsilyl; TBSOTf, *tert*-butyldimethylsilyl trifluoromethanesulfonate; THF, tetrahydrofuran; TMSCl, chlorotrimethylsilane.

The multiple step synthesis begins with preparation of the tricyclic intermediate **17** as shown in Fig. 3a. Homologation of readily available aldehyde **19** (ref. 36) was achieved using the *in situ* generated ylide Ph_3_P=CHOMe followed by workup; reduction of the resultant aldehyde gave alcohol **20** in 55% overall yield in two steps. Subjecting **20** to a sequence of esterification with acryloyl chloride and desilylation by TBAF (tetra-*n*-butylammonium fluoride) generated the olefinic phenol **18**. Having a rapid approach to the precursor **18** established, the stage was thus set for the crucial oxidative dearomatization/IMDA cascade reaction. As anticipated, phenol **18** underwent facile oxidation on exposure to PIDA (iodobenzene diacetate) in MeOH. After switching the solvent from methanol to xylene, the ensuing IMDA cycloaddition proceeded smoothly at 150 °C through the *endo* transition state (**21**), securing the desired bicyclo[2.2.2]octane unit (**22**) as a single diastereomer (Supplementary Figs 11–13). Removal of the two methoxy groups in **22** in the presence of SmI_2_ (ref. 37) followed by subsequent ketone protection furnished **17** in 76% overall yield.

The next step was to assemble the pentacyclic backbone of atropurpuran (Fig. 3a). The cleavage of the lactone in **17** with EtSH, followed by Dess-Martin oxidation of the resultant primary alcohol, led to aldehyde **16** in 65% overall yield. A reductive Knoevenagel condensation between 1,3-cyclohexanedione (**15**) and aldehyde **16** took place smoothly utilizing L-proline as the catalyst and Hantzsch ester as the hydrogen source. Given the limited stability of the resulting product, this intermediate was masked immediately as an enol silane in one pot to give thioester **23** in 82% yield. Conversion of thioester **23** into aldehyde **24** was readily achieved according to Fukuyama's protocol^38^. At this point, we were faced with the construction of two critical C–C bonds (that is, C5–C20 and C9–C10) before arriving at the pentacyclic framework of the target molecule. We reasoned that establishment of the C5–C20 bond first would be beneficial to the installation of the requisite stereocenter at C9. As a result, subjection of the labile silyl enol ether (**24**) to TBAF secured the formation of the E ring to afford tetracyclic diketone **25** and its 20-hydroxyl epimer (91% yield, >20:1 dr).

With the aforementioned strategy in mind, we then focused on investigating the pivotal ketyl-olefin cyclization to set up the B ring. After much experimentation, the cyclization of **25** proved to be challenging in that the starting material remained under most attempted conditions using SmI_2_ as a radical initiator. This could be explained by the assumption that a chair conformation was adopted for the E ring due to its tendency to release the steric repulsion between the cyclohexanedione and the bridged bicyclo[2.2.2]octane system (**25**, Fig. 3b), which resulted in a significant spatial distance between the generated ketyl radical at C10 and the radical acceptor of Δ^9^,^11^ olefin. In addition, the intramolecular H-bonding between the proximal ketone and free hydroxyl group might stabilize this chair conformation. In this context, we hypothesized that a bulky substituent at the C20 position would not only interrupt the H-bonding but also force the formation of a boat conformation for the E ring because of the steric repulsion between the bulky OR group at C20 and the ketone group at C4 (**26**, Fig. 3b). In the boat conformation, the ketone moiety at C10 was in close proximity to the olefin, thus favouring the desired cyclization^33^. Working on this hypothesis, a bulky TBS protecting group was then installed on the C20 hydroxyl group in **25** to give **26**. Gratifyingly, on treatment of intermediate **26** with SmI_2_/HMPA, the expected cyclization proceeded with remarkable efficiency to yield **27** as a single diastereomer in 95% yield. Its structure was unambiguously confirmed by X-ray crystallographic analysis (see Supplementary Methods). Overall, the 14-step sequence enabled an efficient access to the core structure for both arcutane diterpenes and arcutine alkaloids on a gram scale.

### Total synthesis of atropurpuran

**Figure 4:**
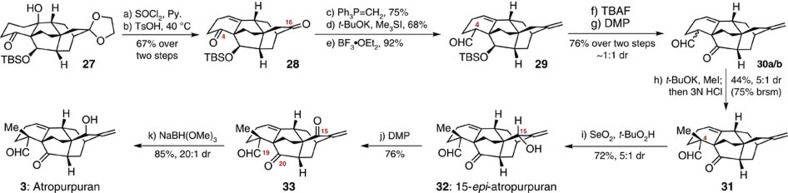
Completion of the total synthesis of (±)-atropurpuran (3). Conditions: (a) SOCl_2_, Py., 0 °C; (b) TsOH, acetone/H_2_O (10:1), 40 °C, 67% over two steps; (c) Ph_3_PCH_3_Br, *t*-BuOK, PhMe, rt, 75%; (d) Me_3_SI, *t*-BuOK, DMSO, THF, rt, 68%; (e) BF_3_·OEt_2_, PhMe, –20 °C, 92%; (f) TBAF, THF, reflux; (g) DMP, CH_2_Cl_2_, rt, 76% over two steps (∼1:1 dr); (h) *t*-BuOK, MeI, *t*-BuOH, rt; then 3N HCl, THF, 44% (75% brsm, 5:1 dr); (i) SeO_2_, *t*-BuO_2_H, CH_2_Cl_2_, 30 °C, 72% (5:1 dr); (j) DMP, CH_2_Cl_2_, rt, 76%; (k) NaBH(OMe)_3_, THF/MeOH (10:1), −30 °C, 85% (20:1 dr). DMP, Dess-Martin periodinane; THF, tetrahydrofuran.

The final steps of our synthesis of (±)-atropurpuran are illustrated in Fig. 4. Dehydration of the tertiary hydroxyl group in **27** with SOCl_2_ in pyridine, followed by removal of dioxolane in aqueous acetone, afforded diketone **28** in 67% overall yield. A three-step transformation including selective Wittig methylenation at C16, Corey-Chaykovsky epoxidation^39^ at C4, and epoxide opening with BF_3_·OEt_2_ (ref. 40) enabled access to aldehyde **29** as a single diastereomer for further methylation at C4. The stereochemistry of the aldehyde group in **29** was not determined at this stage. Because the initial efforts for α-methylation of aldehyde **29** failed, probably due to its sterically hindered environment, desilylation was conducted using TBAF, leading to a pair of inseparable hydroxyl aldehydes along with corresponding mixtures of a cyclic hemiacetal. On treating the above crude mixture with Dess-Martin periodinane, clean keto aldehyde **30** was obtained as a pair of separable diastereomers (∼1:1 dr). As anticipated, under the conditions of *t*-BuOK/MeI, both diastereomers were converted to the desired product **31** and its C4 methyl epimer in 75% yield (5:1 dr) based on recycling of **30a/b** (Supplementary Methods).

With **31** in hand, we envisioned that an allylic oxidation of the exocyclic alkene might give access to our target. However, the oxidation occurred predominately from the less hindered α face and produced the undesired 15-epi-atropurpuran **32** as the major product, along with the minor product atropurpuran **3**. Interestingly, the yield and diastereomeric ratio for the allylic oxidation were observed to be 45% and 14:1, respectively, when using an old SeO_2_ stored in our laboratory, while the yield increased to 72% and the dr value dropped to 5:1 with a freshly ordered SeO_2_, possibly owing to the different reactivity of the two reagents (see Supplementary Methods). After extensive experimentation, inversion of the stereochemistry of the C15 hydroxyl group in **32** proved to be fruitless. In this context, we turned to an oxidation–reduction sequence for setting up the right stereochemistry at C15, which seemed challenging but possible. On one hand, hydride attack to the ketone at C15 (**33**) from the less hindered α face during the reduction would stereoselectively generate the desired C15 hydroxyl group. Furthermore, a chemoselective reduction could be realized through a substrate-controlled manner, owing to the crowded circumstances of two carbonyl groups at C19 and C20 compared with that of C15. Thus, enone **33** was readily prepared via Dess-Martin oxidation of alcohol **32**, before we screened the reduction conditions in detail. Initially, reductants such as reactive diisobutylaluminium hydride, lithium tri-*sec*-butylborohydride (L-selectride), and NaBH_4_–CeCl_3_ resulted in over-reduction even at a low temperature (–78 °C). In contrast, no reaction was observed using the less reactive sodium triacetoxyborohydride, sodium triethylborohydride and potassium tri-*sec*-butylborohydride (K-selectride) at temperatures below –30 °C. Finally, it was found that reduction of enone **33** with NaBH_4_ at –60 °C in tetrahydrofuran/MeOH (10:1) for 1 h provided atropurpuran **3** and 15-*epi*-atropurpuran **32** in 75% yield and 2:1 dr, without reduction of the carbonyl groups at C19 and C20. The stereoselectivity was greatly improved to 20:1 dr to give atropurpuran **3** with 85% yield when more bulky sodium trimethoxyborohydride was employed as the reductant at –30 °C in tetrahydrofuran/MeOH (10:1) for 16 h. The spectral data of synthetic atropurpuran **3** were identical in all respects to those reported for the natural product^11^.

## Discussion

In summary, we have disclosed an expedient and scalable approach to synthesize the core structure for arcutane diterpenes and related alkaloids and achieved the first total synthesis of (±)-atropurpuran. The oxidative dearomatization/IMDA cascade was employed as a key reaction in constructing the bicyclo[2.2.2]octane ring. Sequential aldol and ketyl-olefin cyclizations were used to secure the highly bridged tetracyclo[5.3.3.0^4,9^.0^4,12^]tridecane unit with excellent stereoselectivity. Of particular note, a chemoselective^41^ and stereoselective reduction allowed us to install the requisite allylic hydroxyl group in the target atropurpuran. Having had success with this synthetic approach, our future efforts will focus on pursuit of an asymmetric version, as well as an extension to the total synthesis of corresponding diterpenoid alkaloids.

## Methods

### General

All reactions that require anhydrous conditions were performed in flame-dried glassware under Ar atmosphere and all reagents were purchased from commercial suppliers. Solvent purification was conducted according to Purification of Laboratory Chemicals^42^. The products were purified by flash column chromatography on silica gel (200–300 meshes).

The reactions were monitored by thin layer chromatography. Visualization was accomplished with ultraviolet light, exposure to iodine, staining with an ethanolic solution of phosphomolybdic acid or basic solution of KMnO_4_. ^1^H nuclear magnetic resonance (NMR) and ^13^C NMR spectra were recorded on Varian INOVA-400/54 and Agilent DD2-600/54 instruments and calibrated by using residual undeuterated chloroform (δ, ^1^H NMR=7.260, ^13^C NMR=77.00). The following abbreviations were used to explain the multiplicities: br, broad; d, doublet; dt, double triplet; m, multiplet; s, singlet; t, triplet; td, triple doublet; q=quartet and coupling constants (*J*) are reported in Hertz (Hz). Infrared (IR) spectra were recorded on a Perkin Elmer Spectrum Two fourier transform infrared spectrometer. High-resolution mass spectra were recorded on Bruker Apex IV FTMS or Thermo Scientific LTQ Orbitrap XL ESI mass spectrometers.

### Experimental data

For NMR spectra of synthetic intermediates, see Supplementary Figs 1–52. For the comparisons of ^1^H and ^13^C NMR spectra of the natural and synthetic atropurpuran, see Supplementary Figs 53–58. For the comparisons of ^1^H and ^13^C NMR spectroscopic data of the natural and synthetic atropurpuran, see Supplementary Tables 1 and 2. For the experimental procedures and spectroscopic and physical data of compounds and the crystallographic data of compound **27**, see Supplementary Methods.

### Data availability

The X-ray crystallographic coordinates for structure **27** reported in this study have been deposited at the Cambridge Crystallographic Data Centre (CCDC) with the accession code CCDC 1435700 (www.ccdc.cam.ac.uk/data_request/cif). The authors declare that all other relevant data supporting the findings of this study are available within the article and its Supplementary Information files.

## Additional information

**How to cite this article:** Gong, J. *et al*. Total synthesis of atropurpuran. *Nat. Commun.* 7:12183 doi: 10.1038/ncomms12183 (2016).

## Supplementary Material

Supplementary Information Supplementary Figures 1-58, Supplementary Tables 1-2 and Supplementary Methods

## Figures and Tables

**Figure 1:**
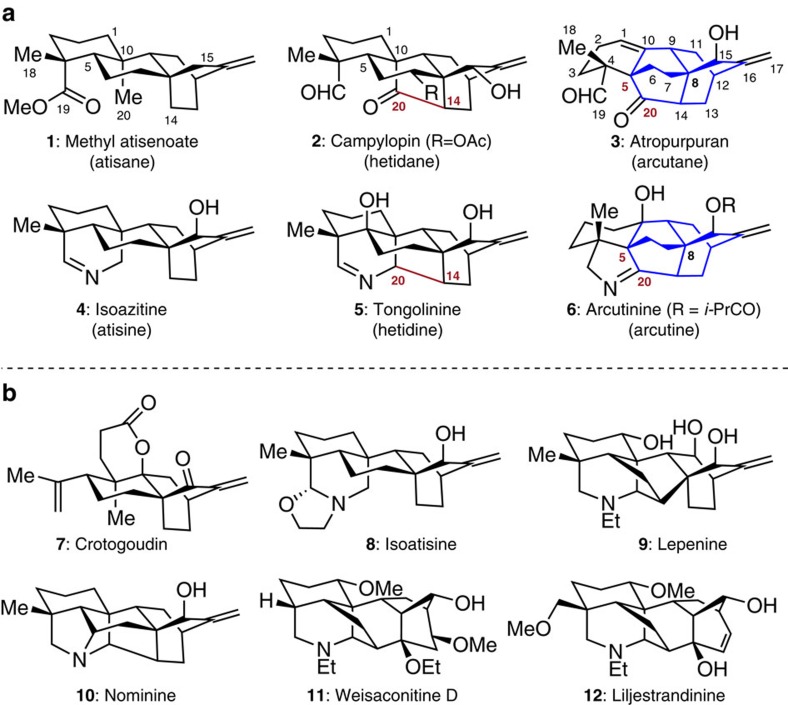
Three-dimensionally cage-like diterpenes and diterpenoid alkaloids. (**a**) Selected members of three related types of diterpenes and their alkaloidal congeners. Among them, the arcutane diterpenes (that is, **3**) and arcutine alkaloids (that is, **6**), containing a unique tetracyclo[5.3.3.0^4,9^.0^4,12^]tridecane moiety (shown in blue bonds) and two all-carbon bridgehead quaternary centres (C5 and C8), posed formidable synthetic challenges. (**b**) Recent accomplishments in the total synthesis of complex atisane-type diterpenes and diterpenoid alkaloids. Ac, acetyl; *i*-Pr, isopropyl.
