# Supplementary material for: Total synthesis of atropurpuran
Source: Nat Commun. 2016 Jul 8;7:12183. doi: 10.1038/ncomms12183 (PMC4941107; doi:10.1038/ncomms12183)
Supplement: Supplementary Information — Supplementary Figures 1-58, Supplementary Tables 1-2 and Supplementary Methods [file ncomms12183-s1.pdf]

Supplementary Figures

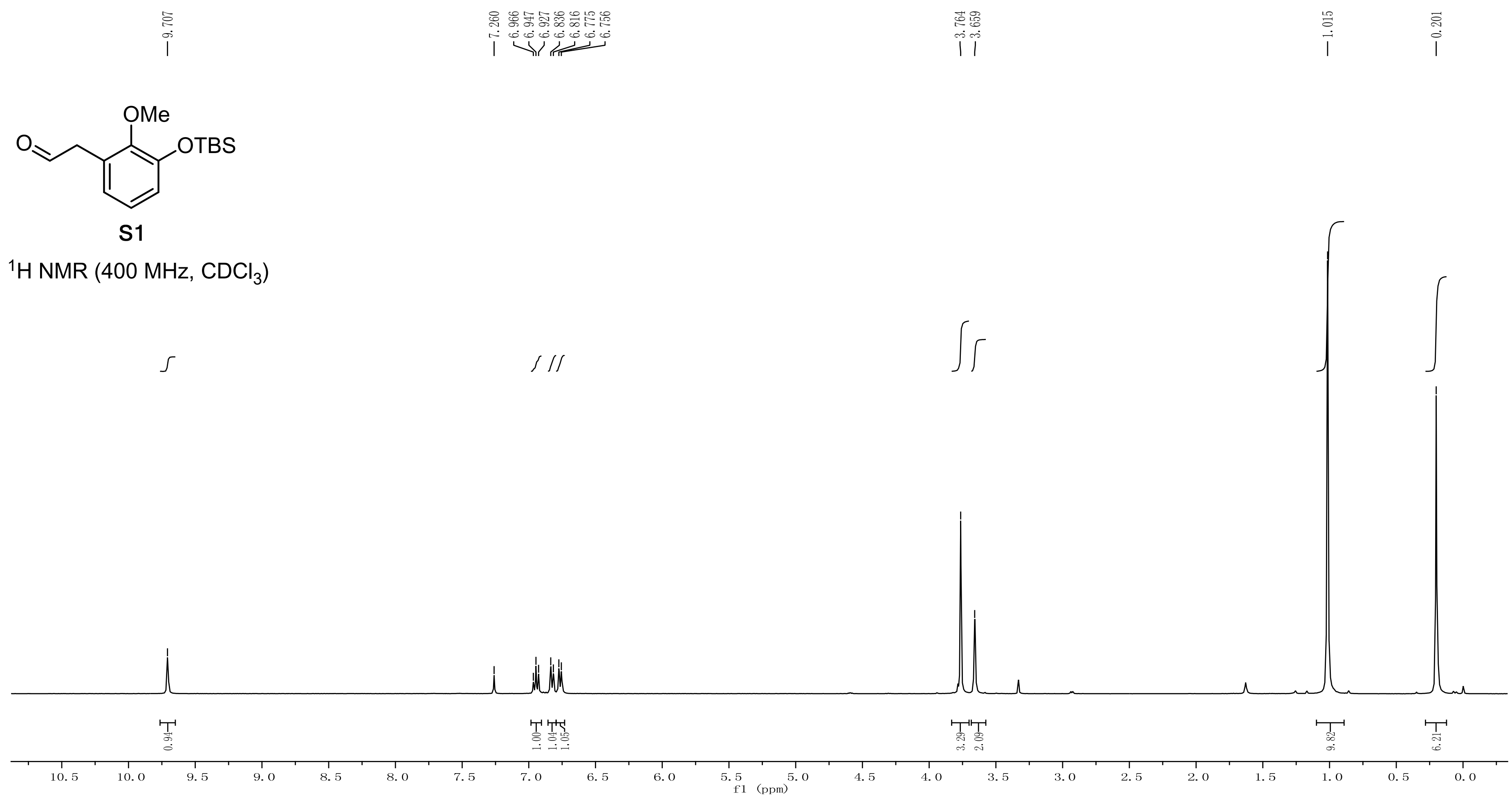

Supplementary Figure 1. ^{1}H NMR spectrum of S1

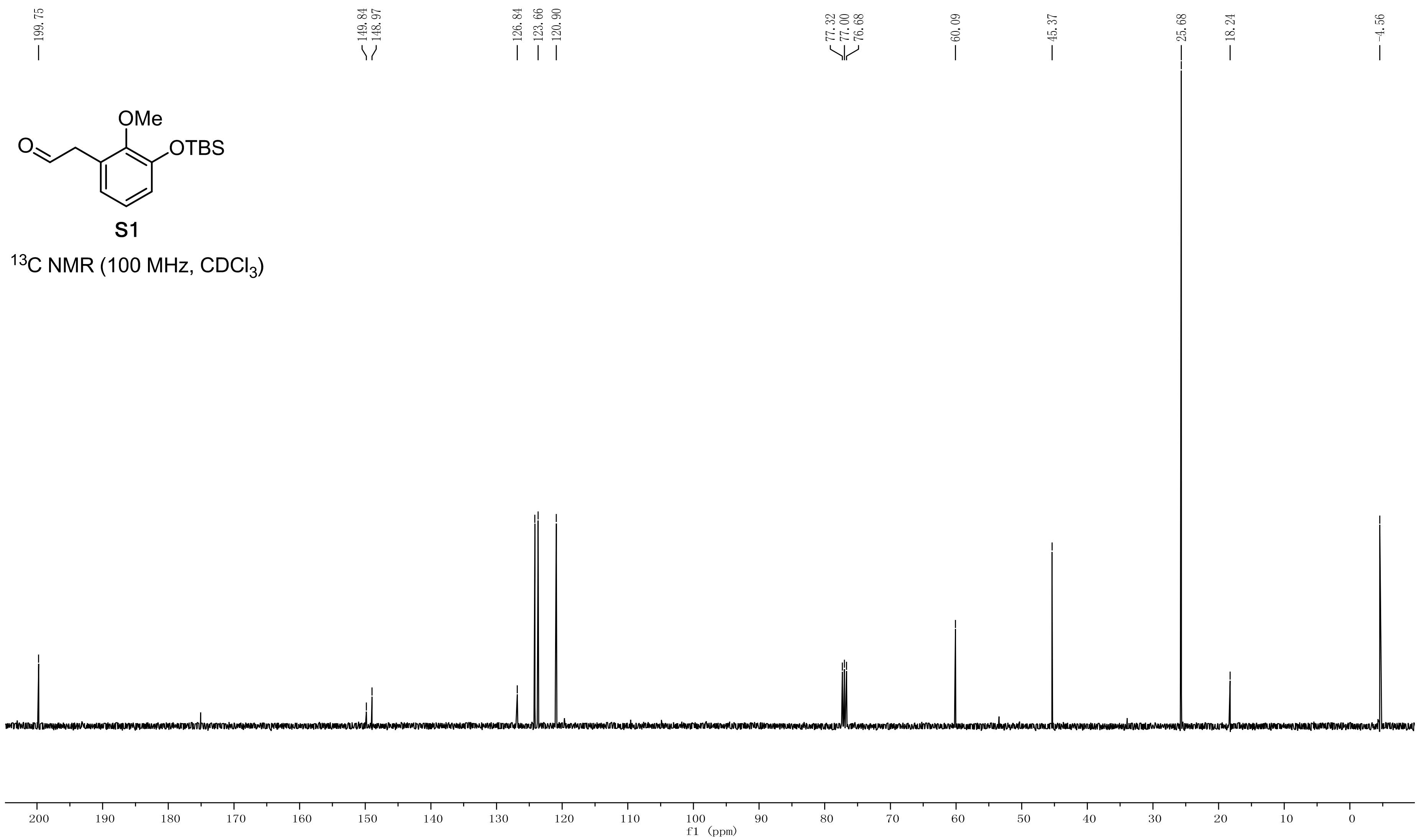

Supplementary Figure 2. ^{13}C NMR spectrum of S1

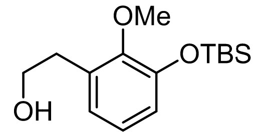

20

^{1}H NMR (400 MHz, $CDCl_3$)

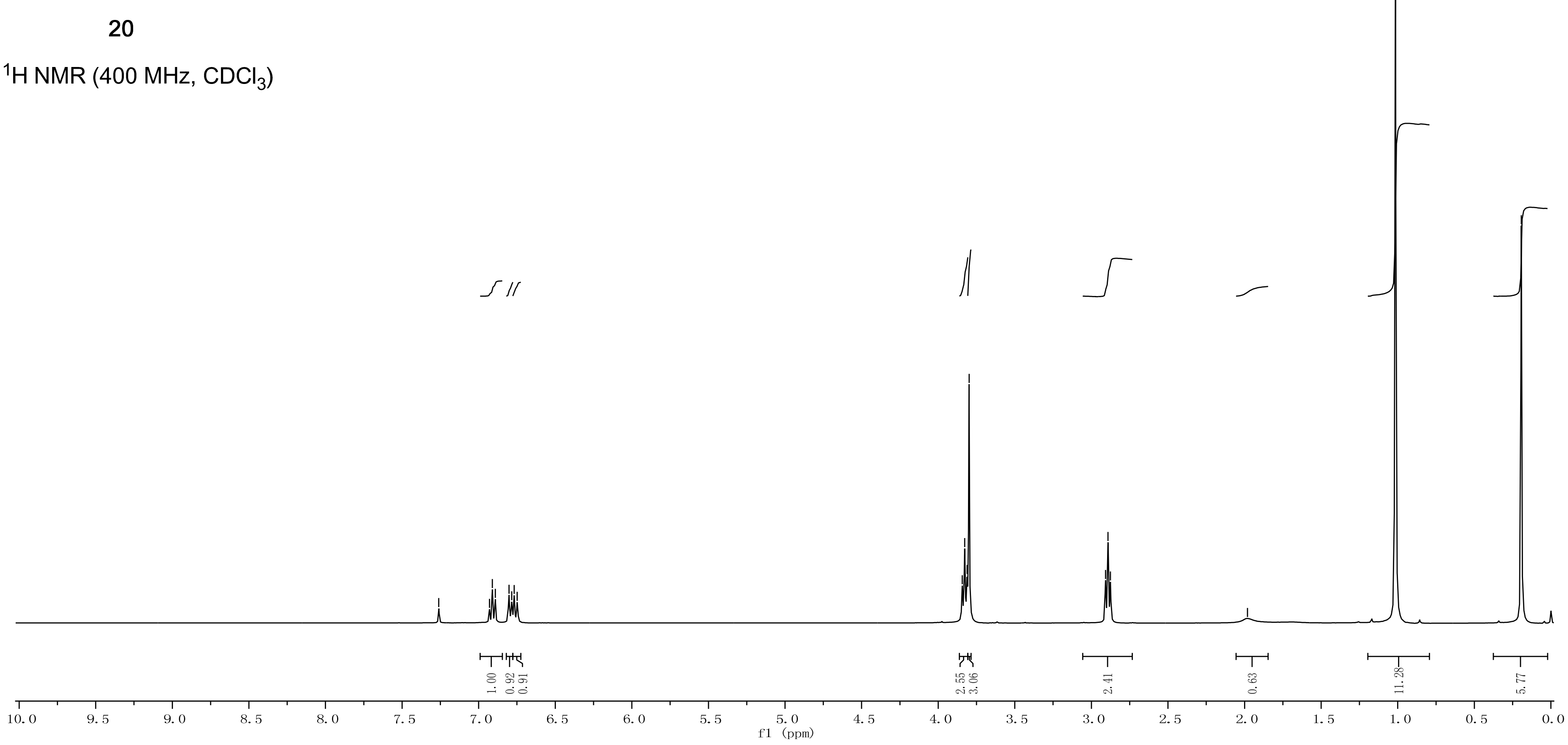

Supplementary Figure 3. ^{1}H NMR spectrum of 20

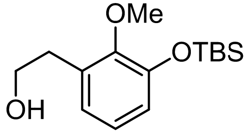

20

^{13}C NMR (100 MHz, $CDCl_3$)

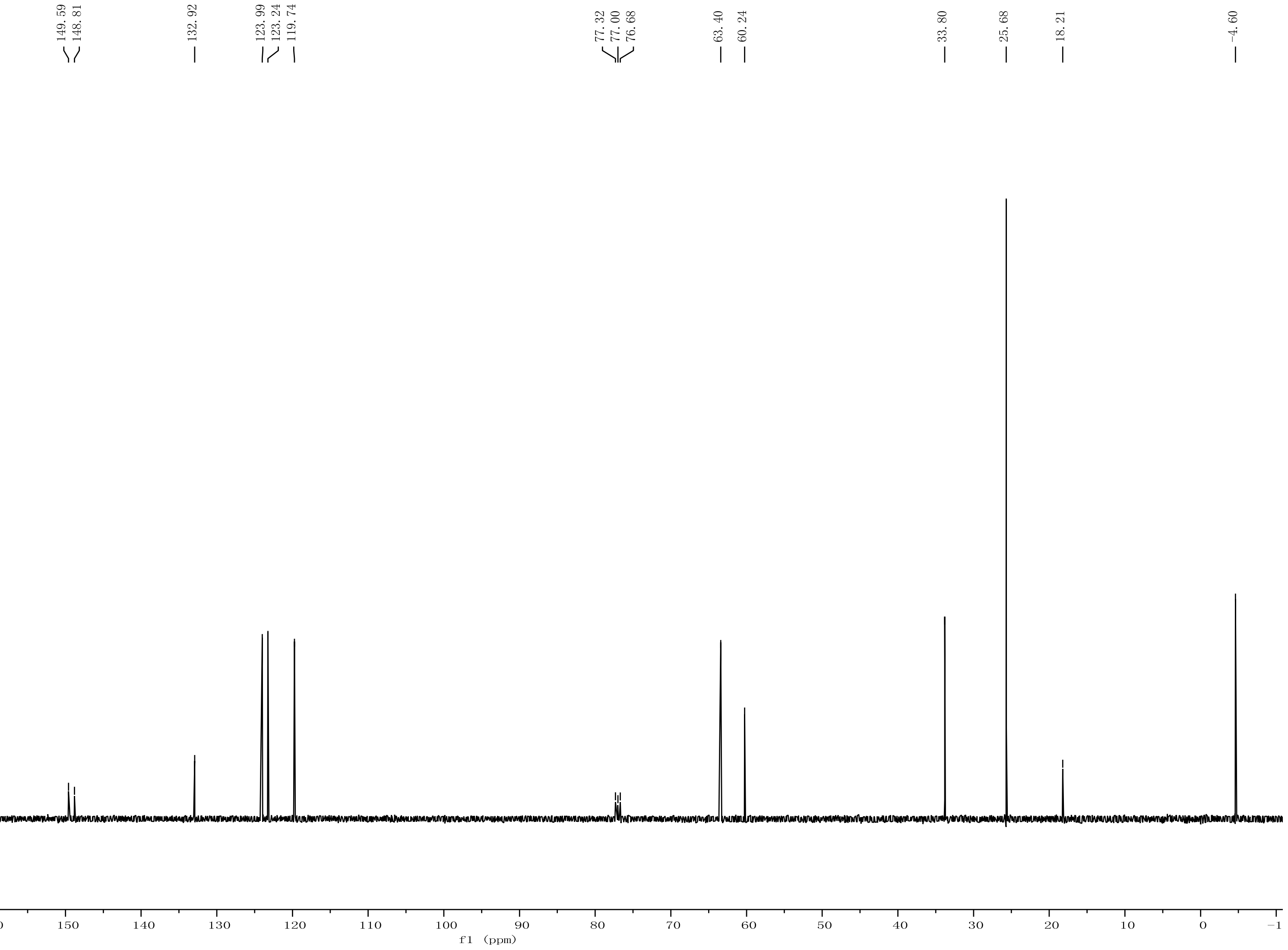

Supplementary Figure 4. ^{13}C NMR spectrum of 20

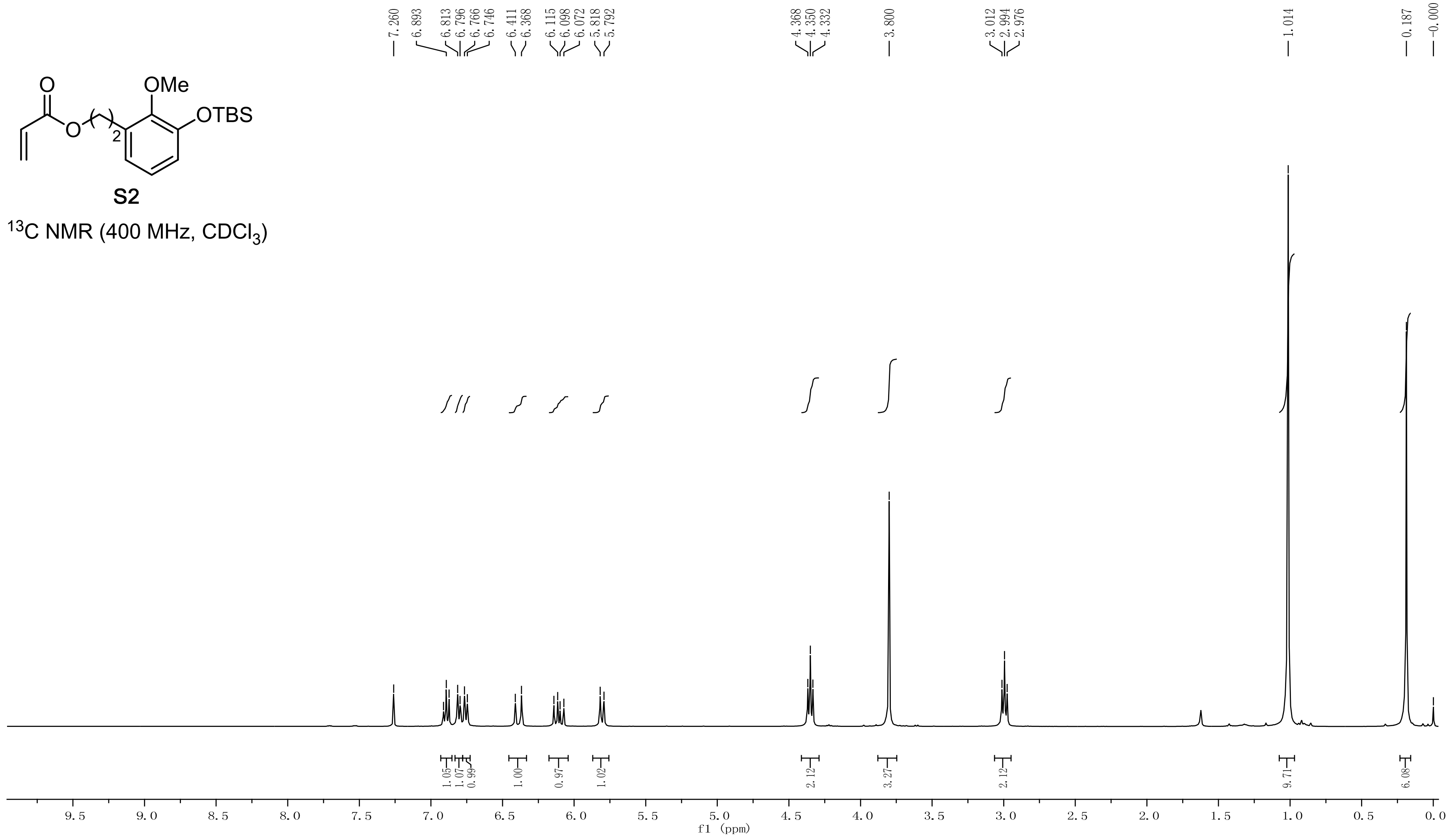

Supplementary Figure 5. ^{1}H NMR spectrum of S2

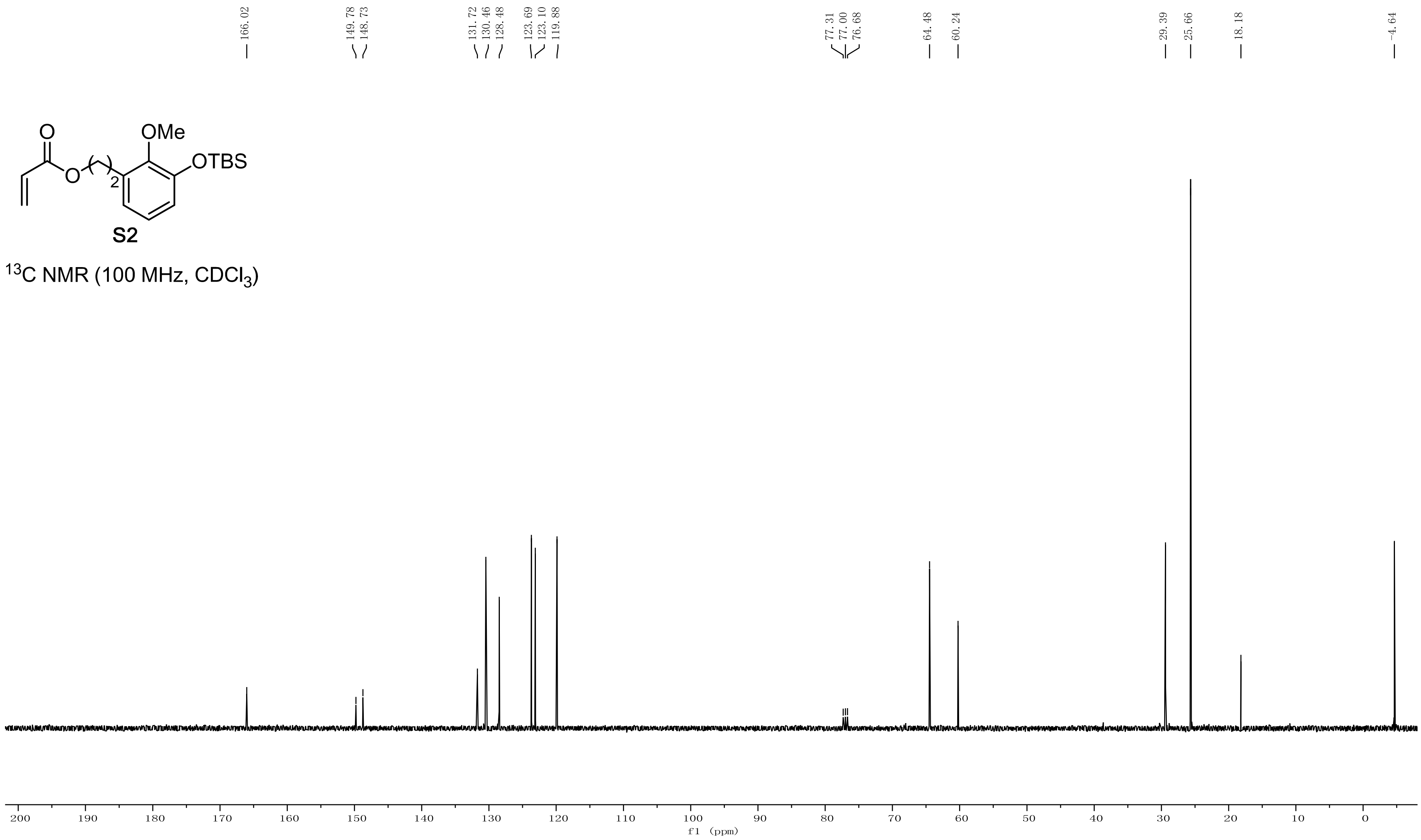

Supplementary Figure 6. ^{13}C NMR spectrum of S2

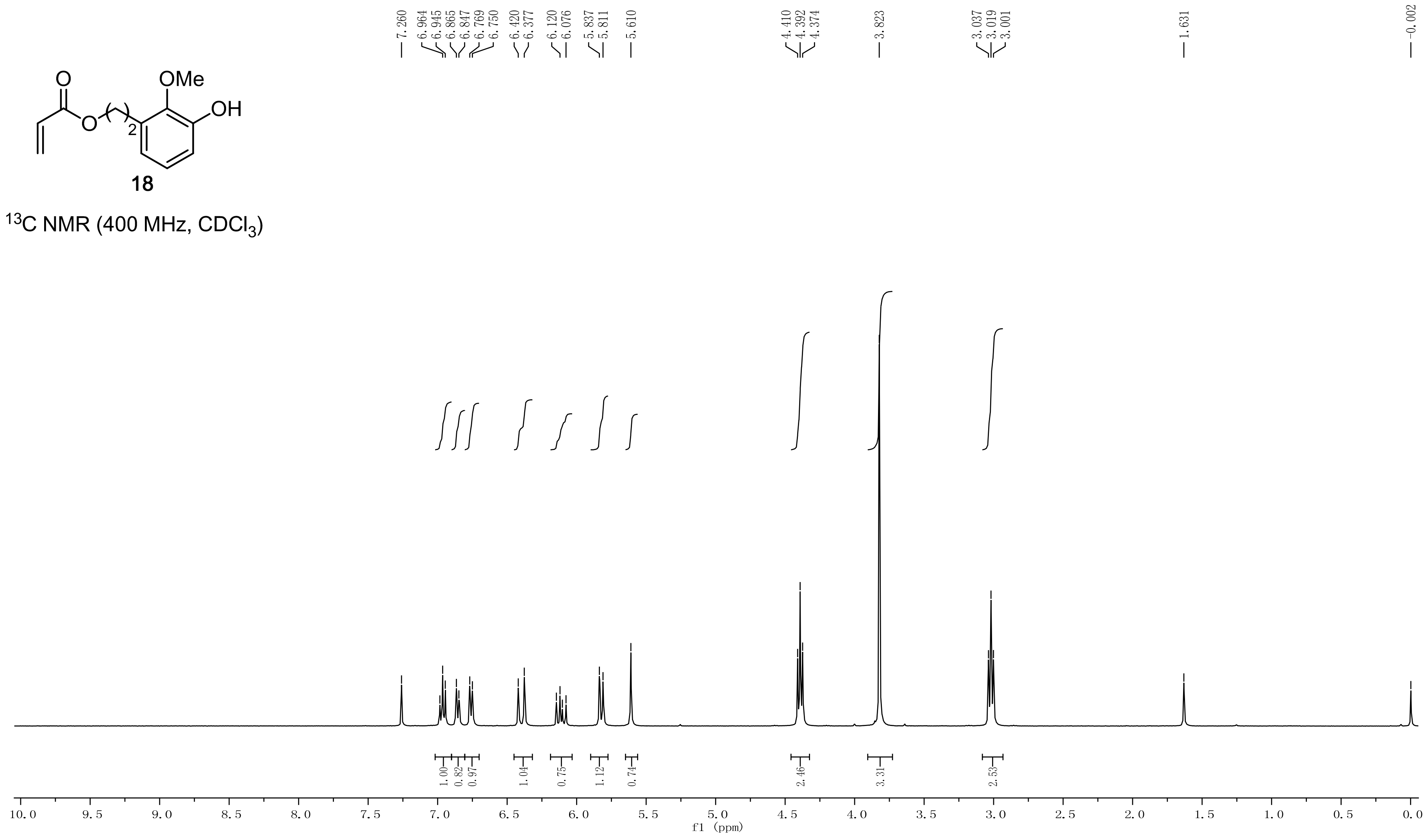

Supplementary Figure 7. ^{1}H NMR spectrum of 18

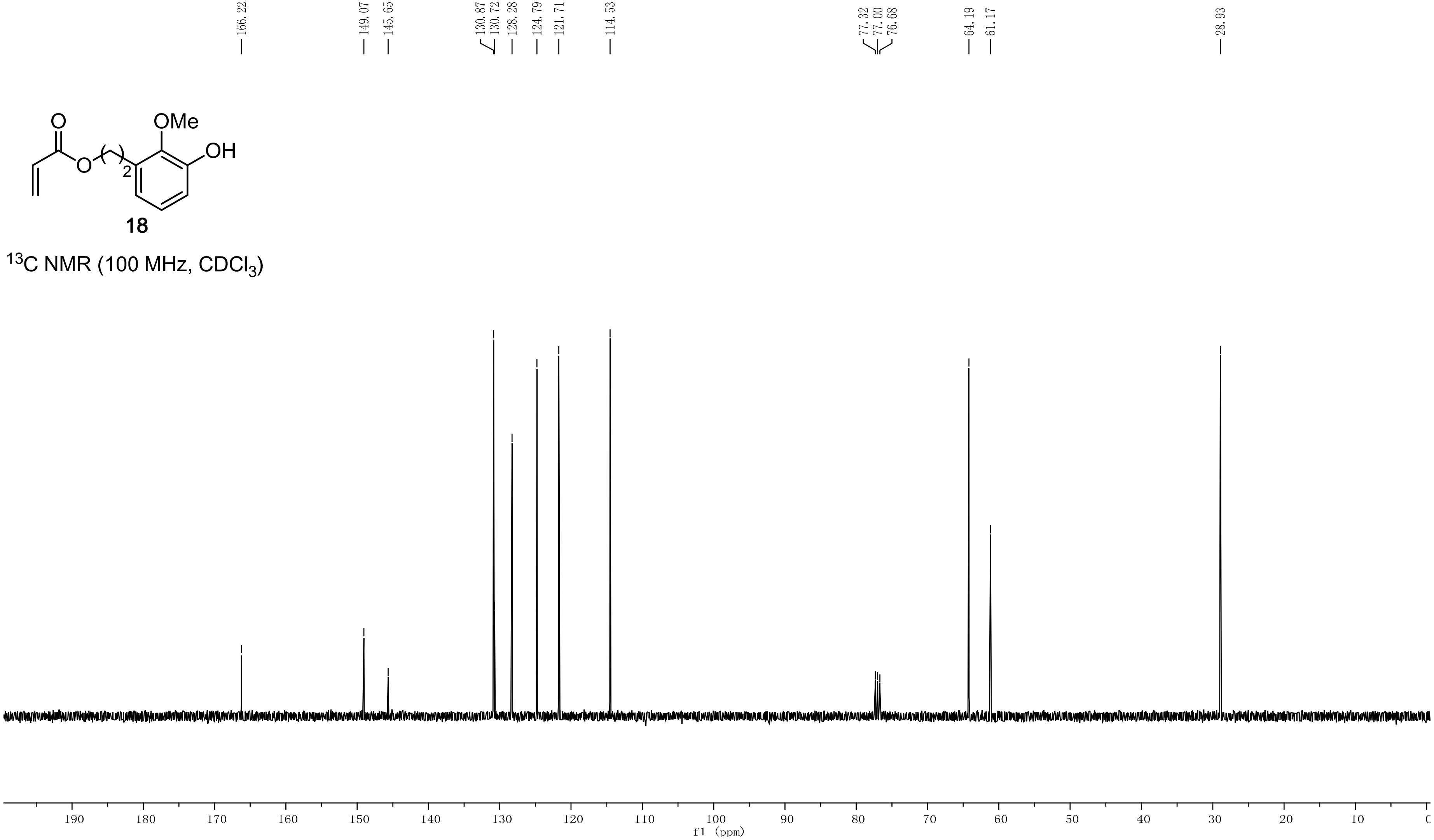

Supplementary Figure 8. ^{13}C NMR spectrum of 18

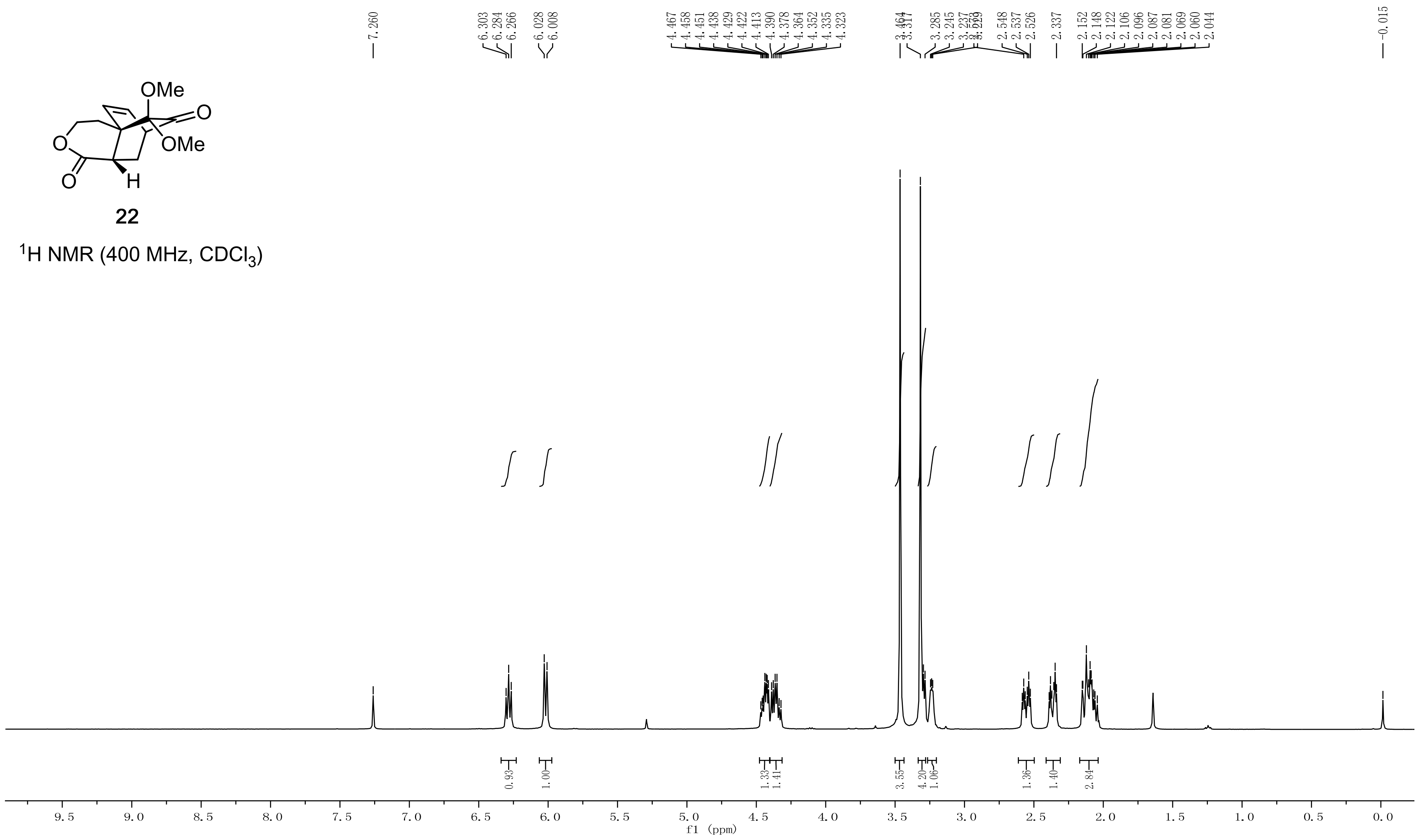

Supplementary Figure 9. ^{1}H NMR spectrum of 22

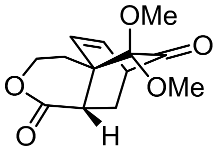

22

^{13}C NMR (100 MHz, $CDCl_3$)

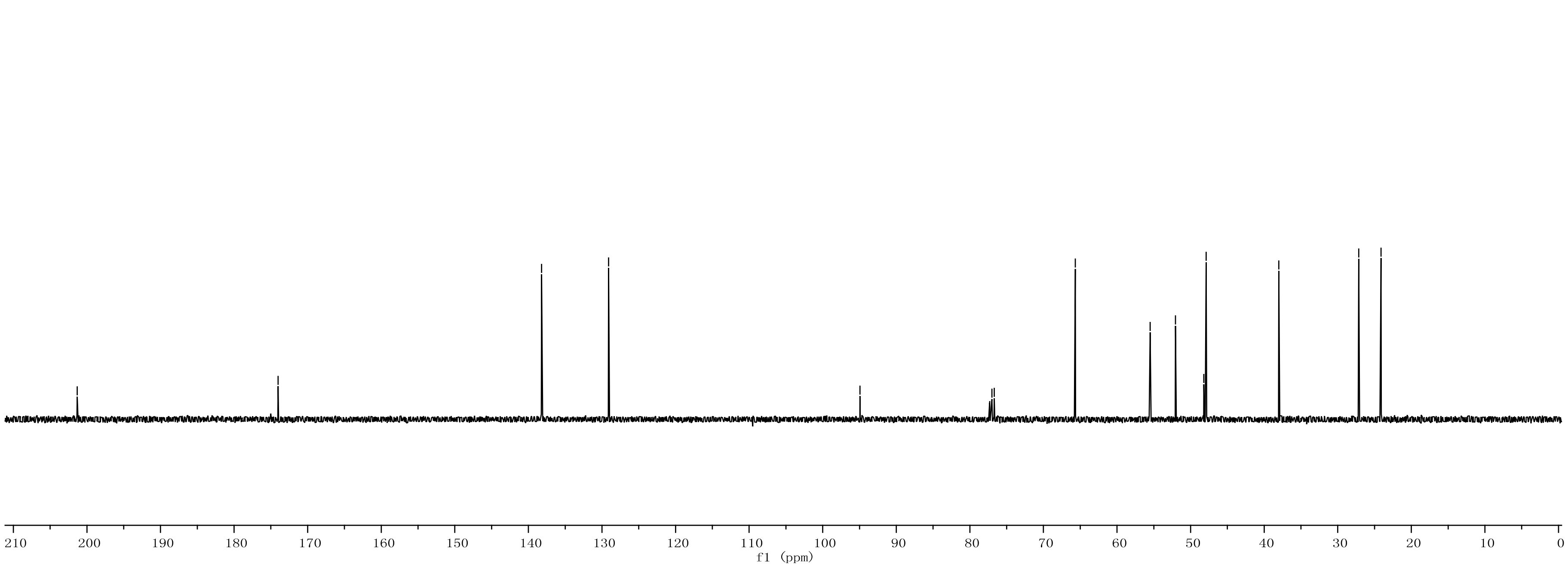

Supplementary Figure 10. ^{13}C NMR spectrum of 22

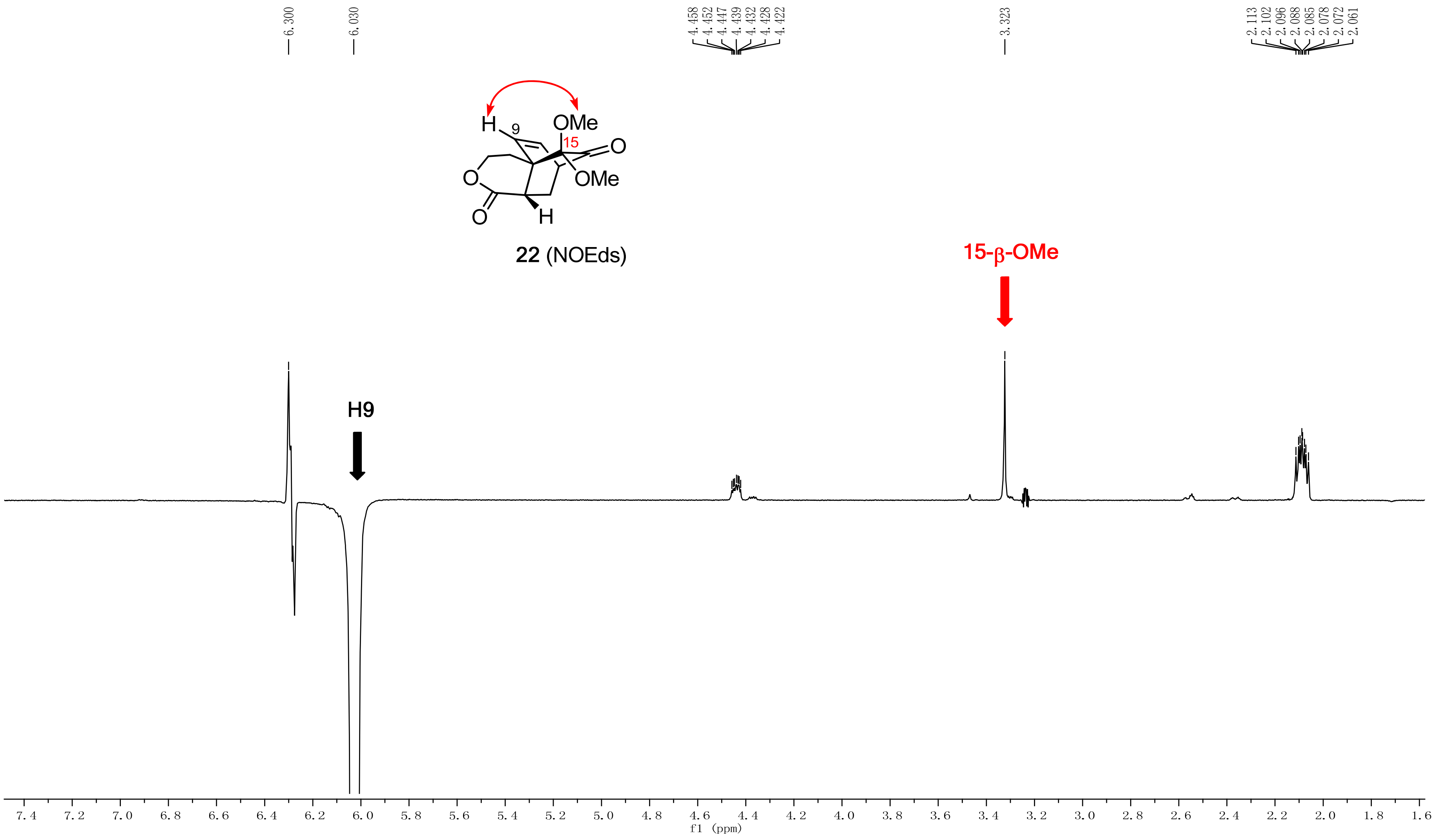

Supplementary Figure 11. NOEds of 22

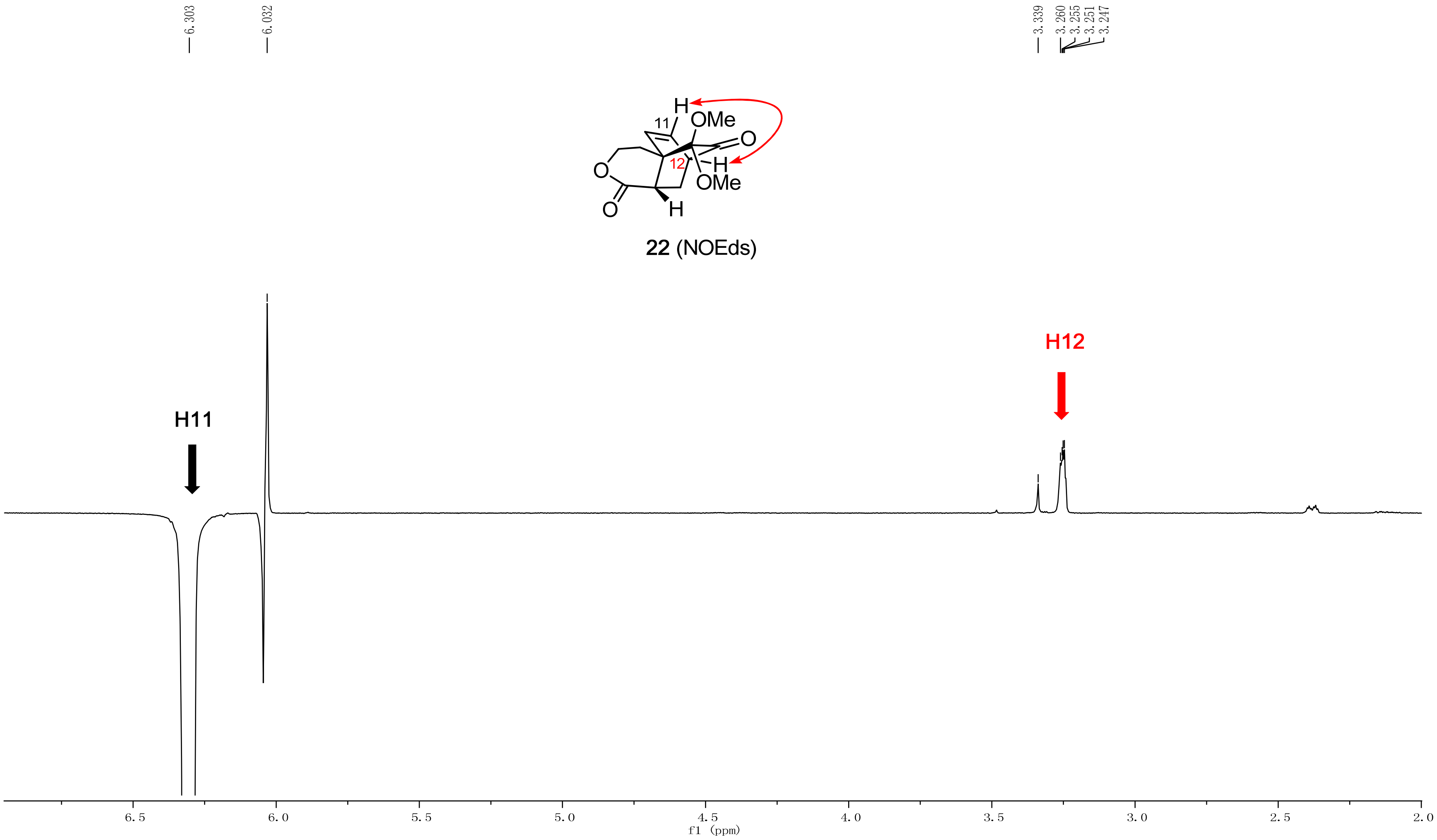

Supplementary Figure 12. NOEds of 22

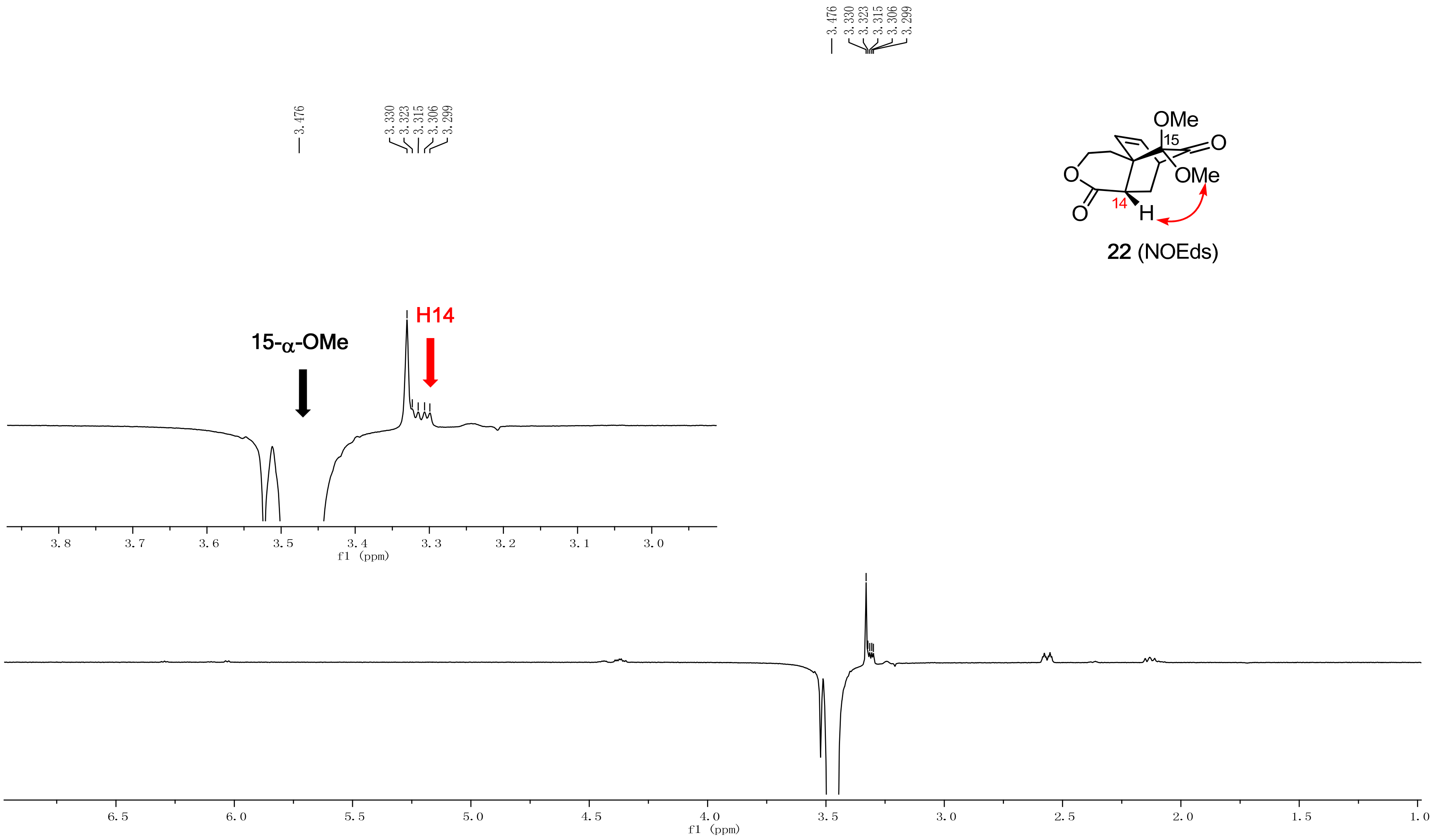

22 (NOEds)

Supplementary Figure 13. NOEds of 22

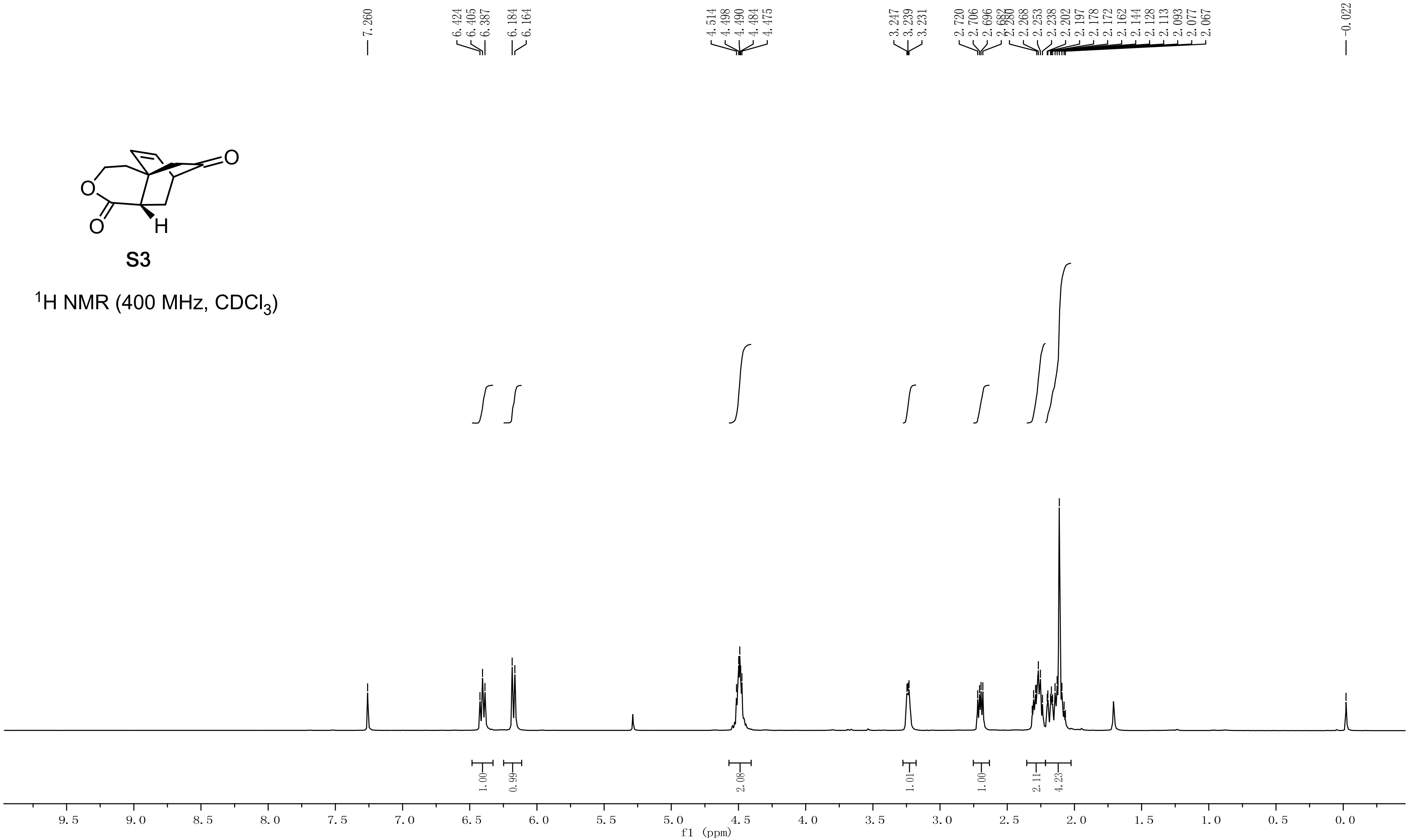

Supplementary Figure 14. ^{1}H NMR spectrum of S3

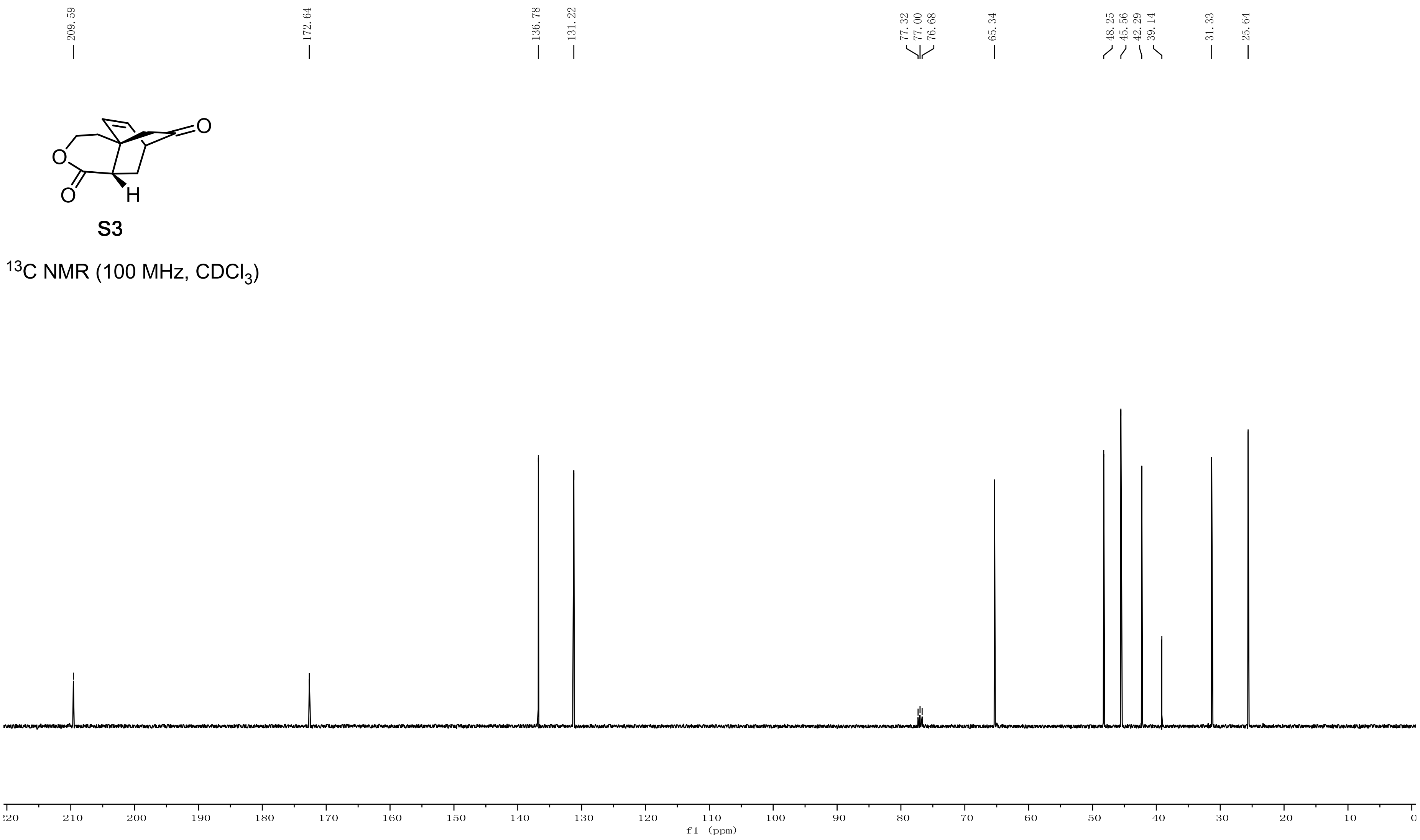

Supplementary Figure 15. ^{13}C NMR spectrum of S3

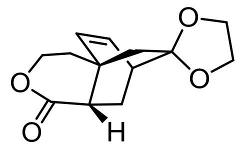

17

^1H NMR (400 MHz, $CDCl_3$)

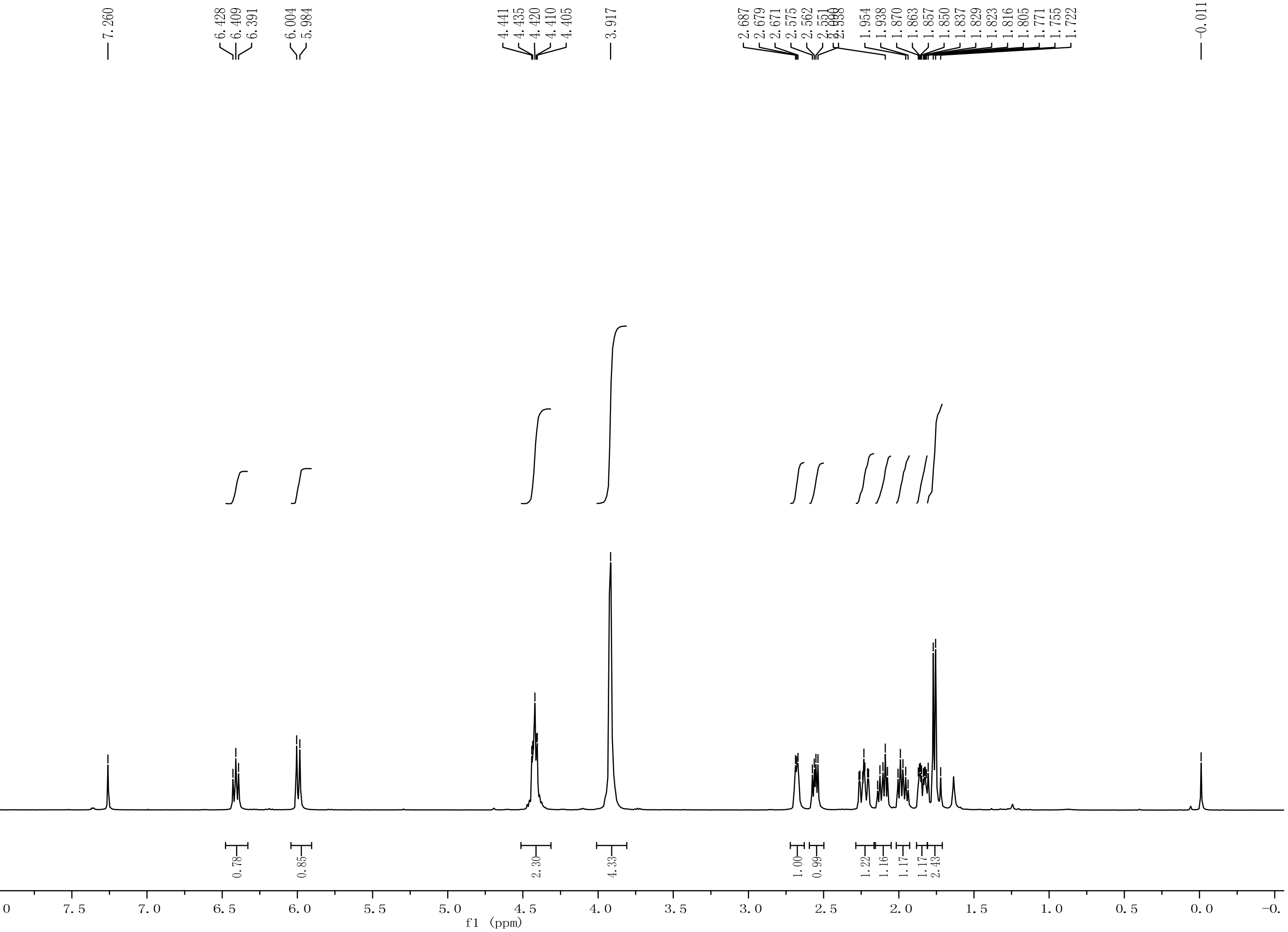

Supplementary Figure 16. ^1H NMR spectrum of 17

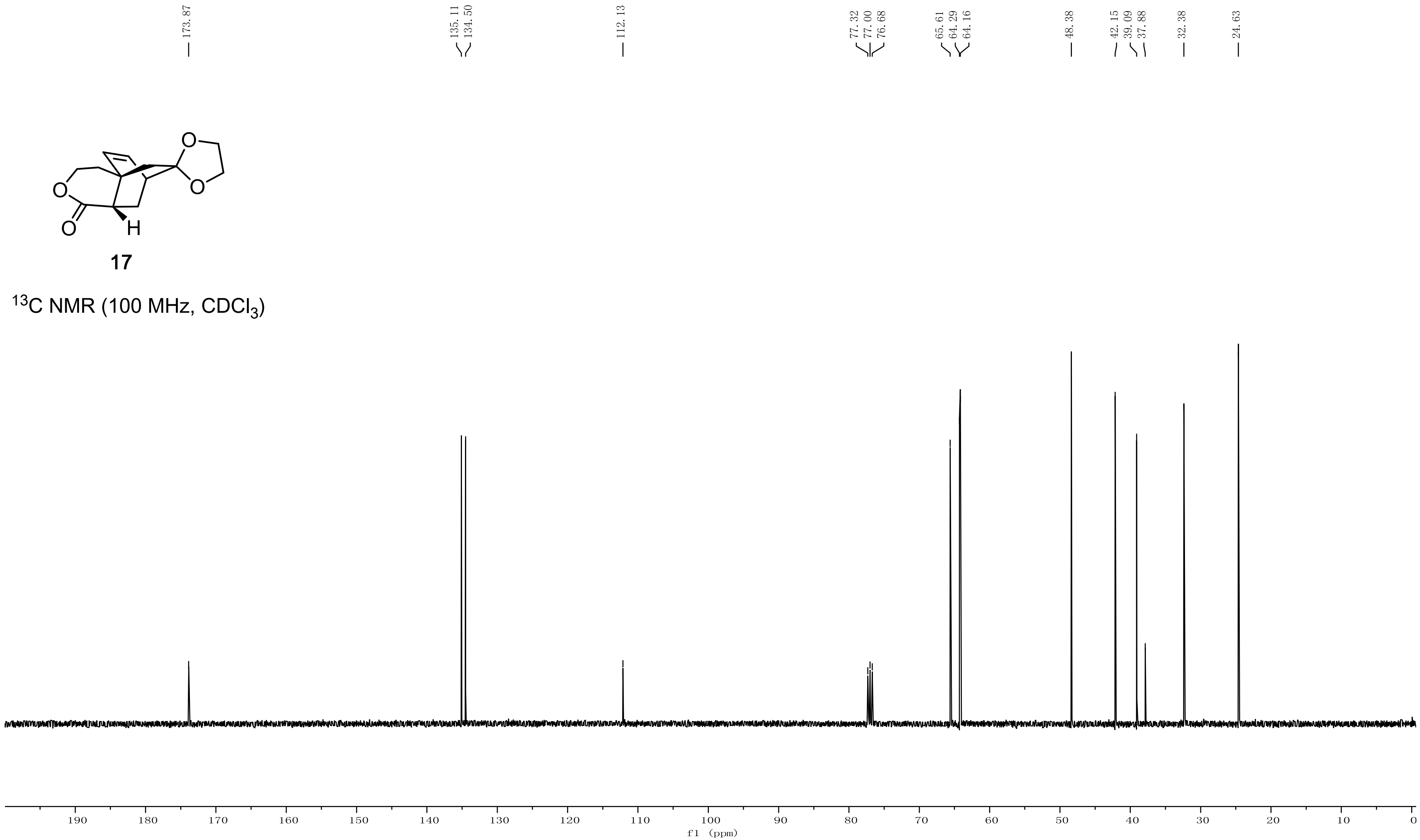

Supplementary Figure 17. ^{13}C NMR spectrum of 17

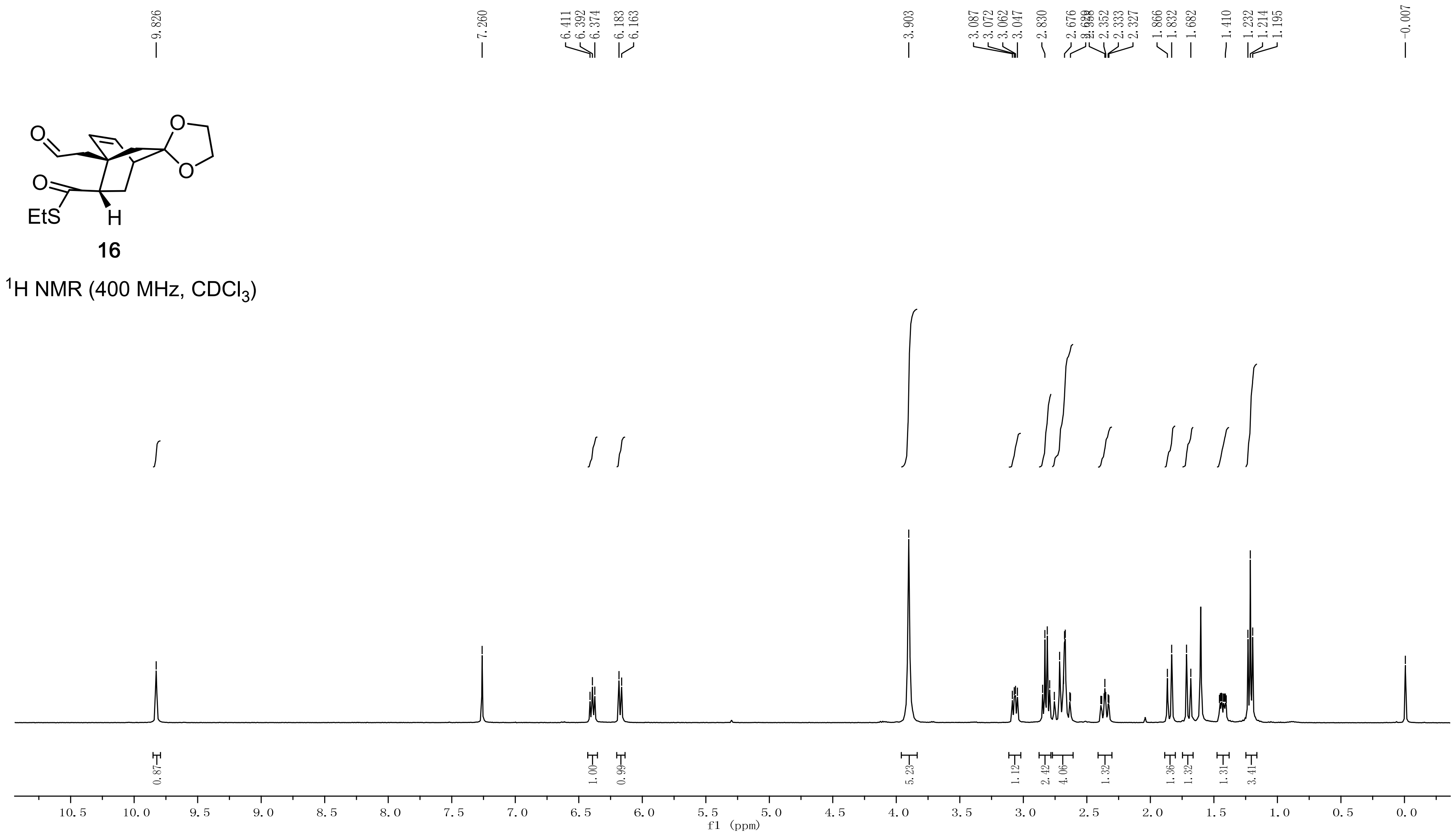

Supplementary Figure 18. ^{1}H NMR spectrum of 16

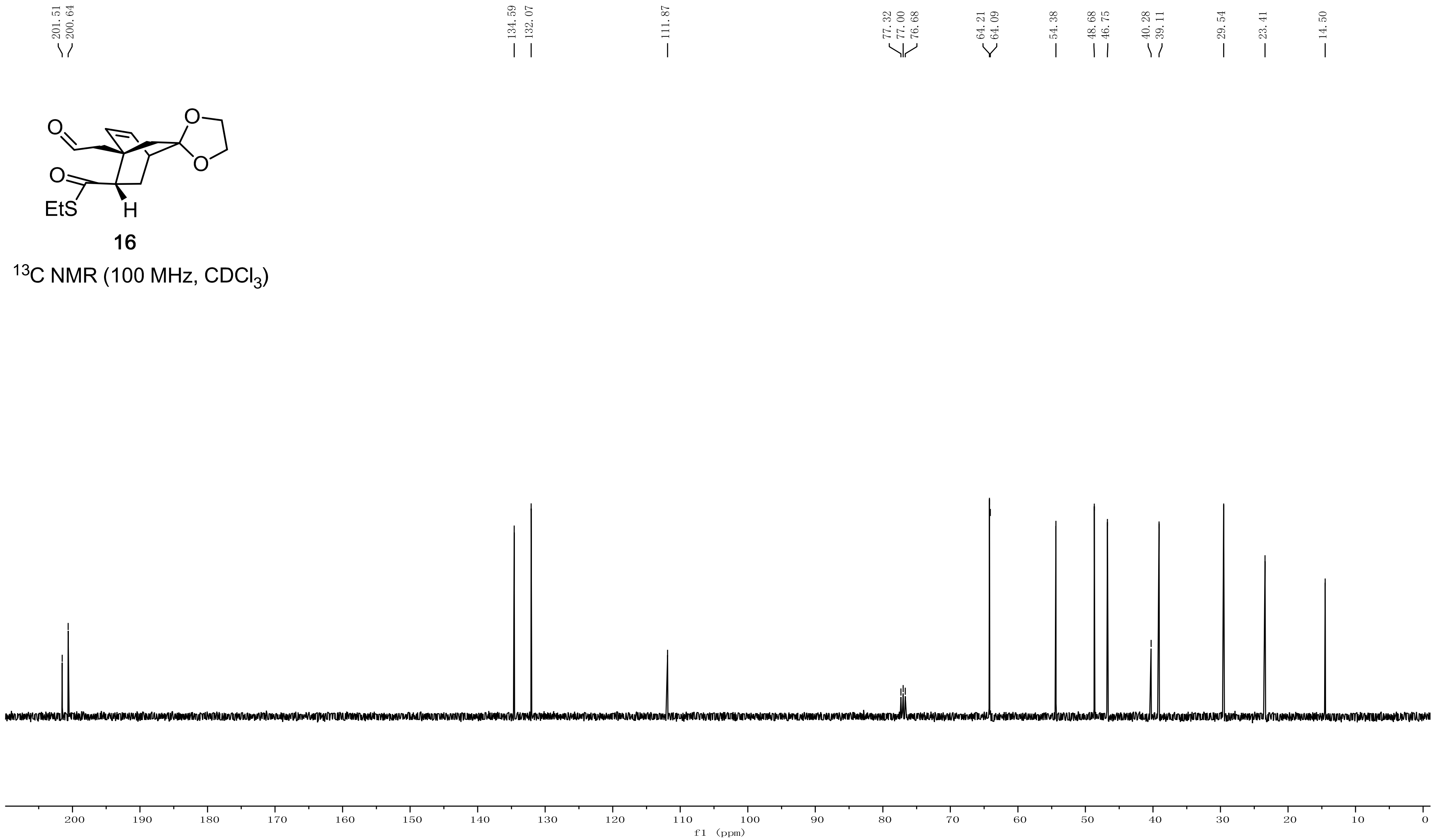

16

^{13}C NMR (100 MHz, $CDCl_3$)

Supplementary Figure 19. ^{13}C NMR spectrum of 16

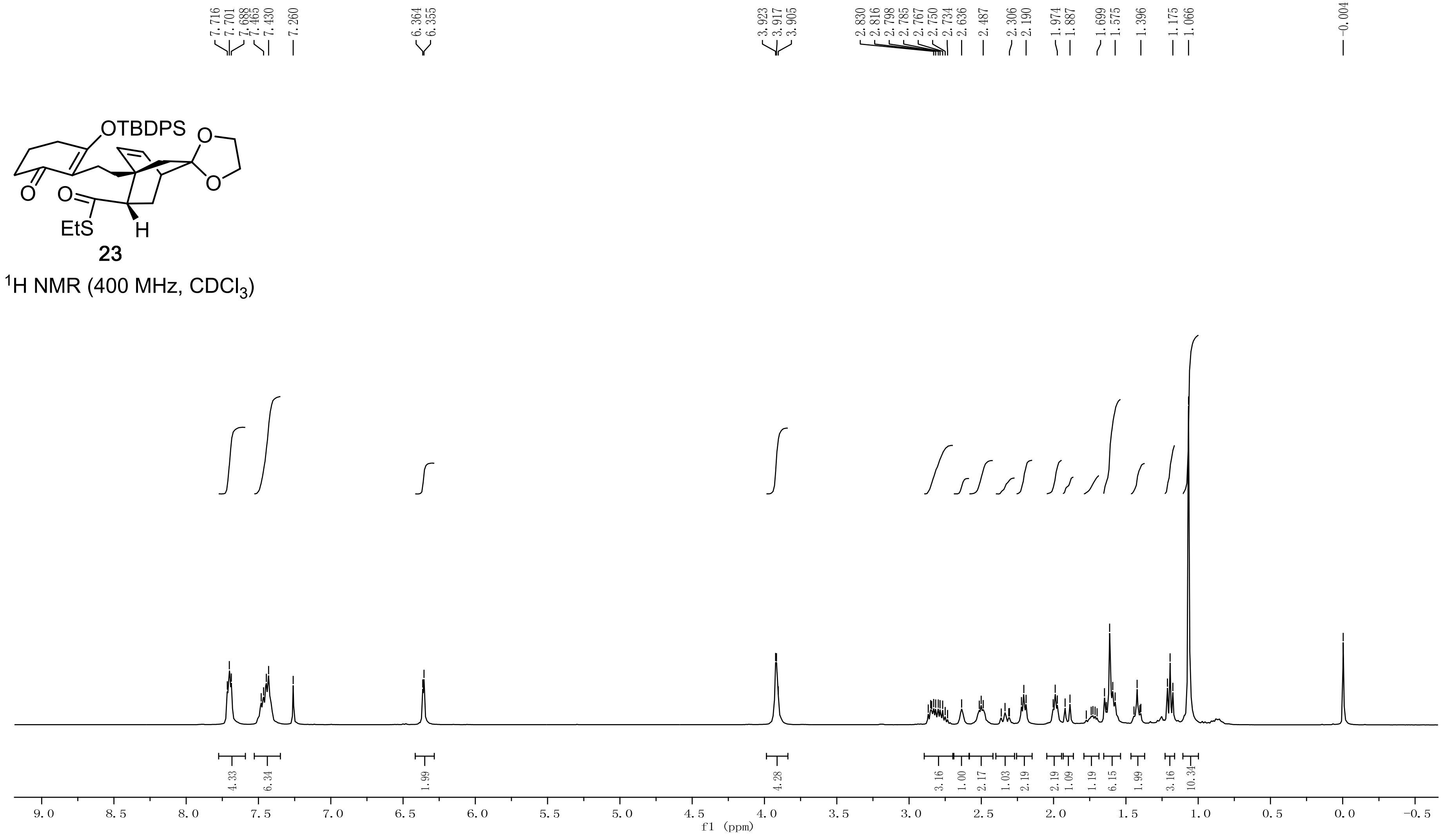

Supplementary Figure 20. ^{1}H NMR spectrum of 23

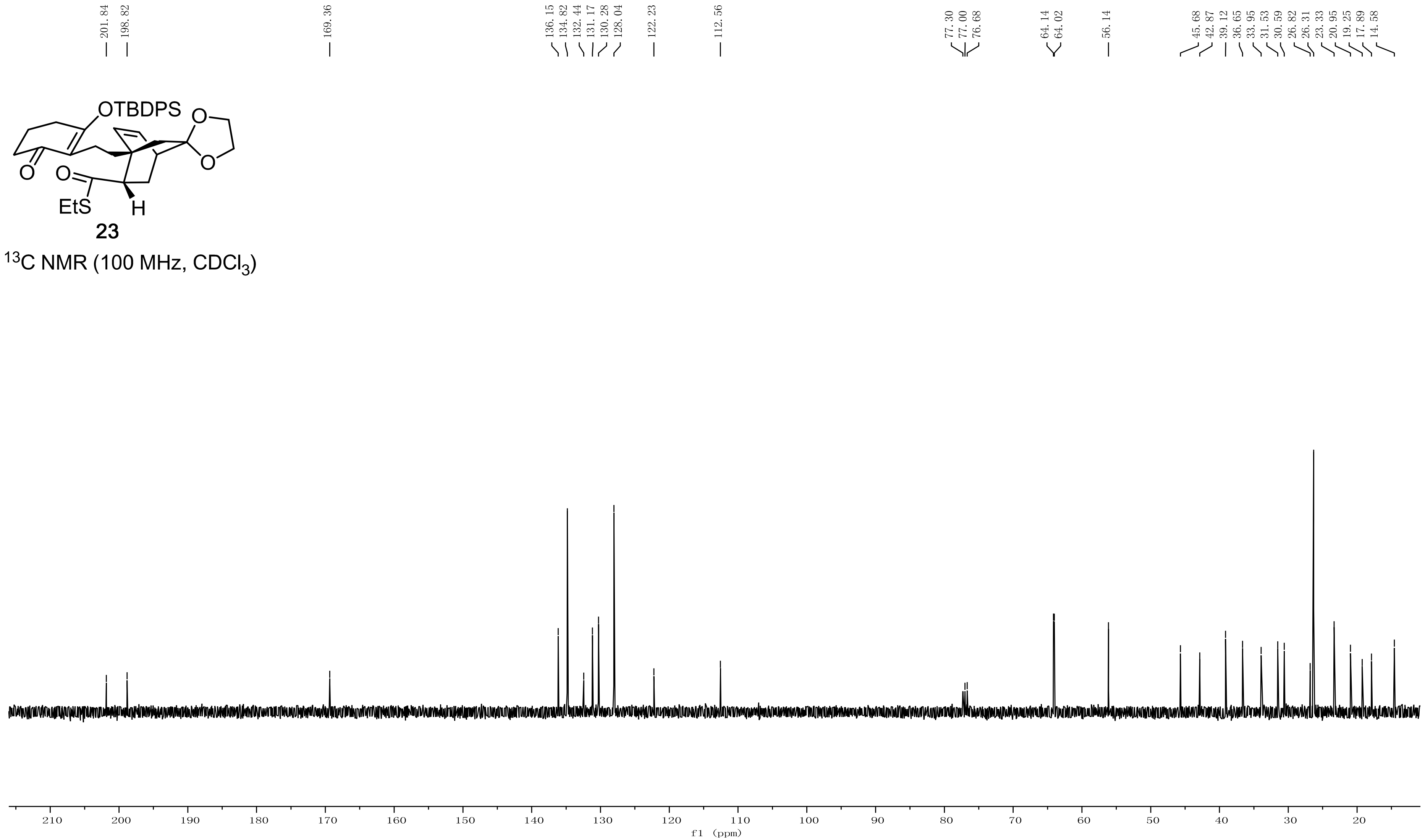

Supplementary Figure 21. ^{13}C NMR spectrum of 23

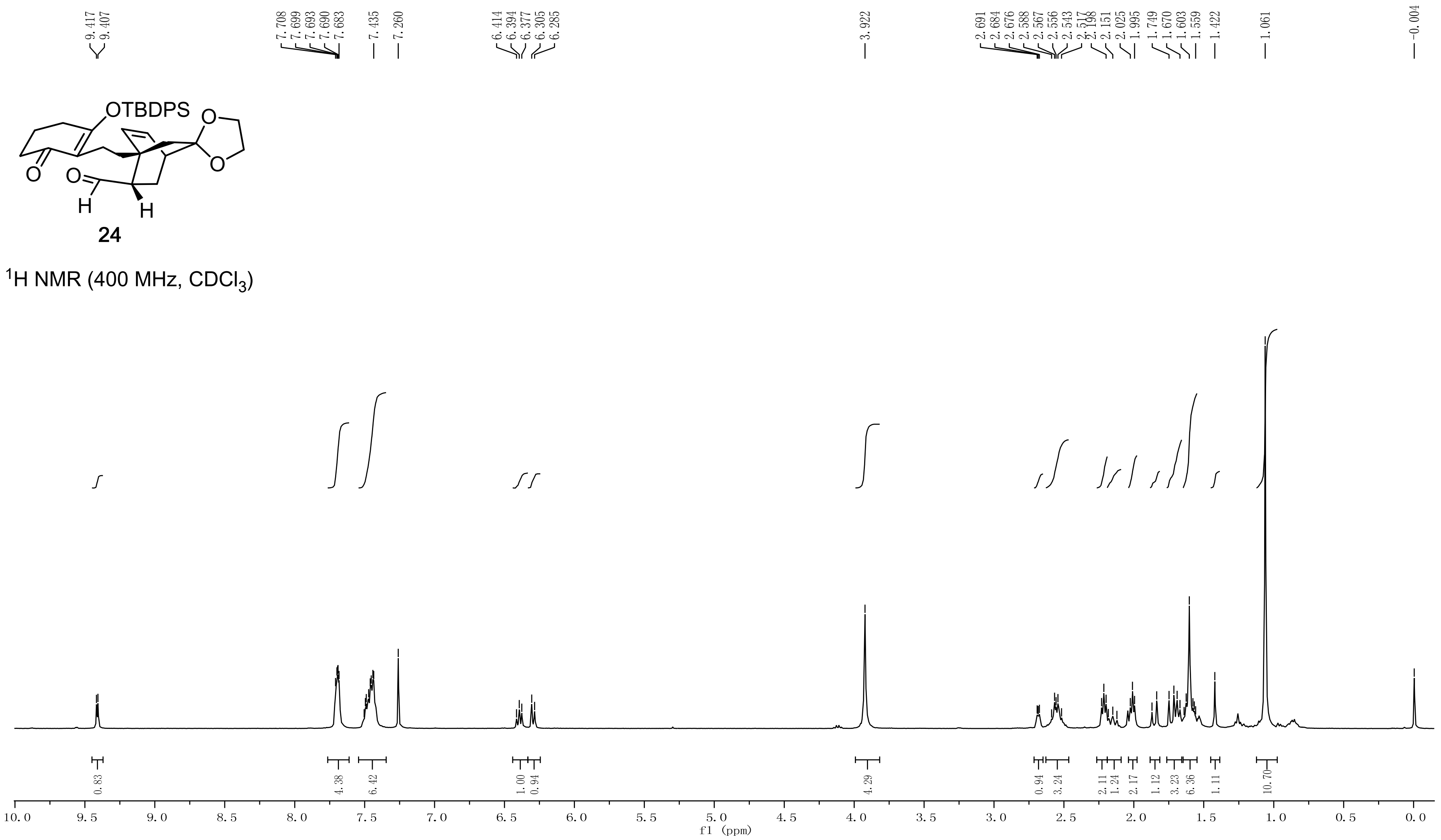

Supplementary Figure 22. ^{1}H NMR spectrum of 24

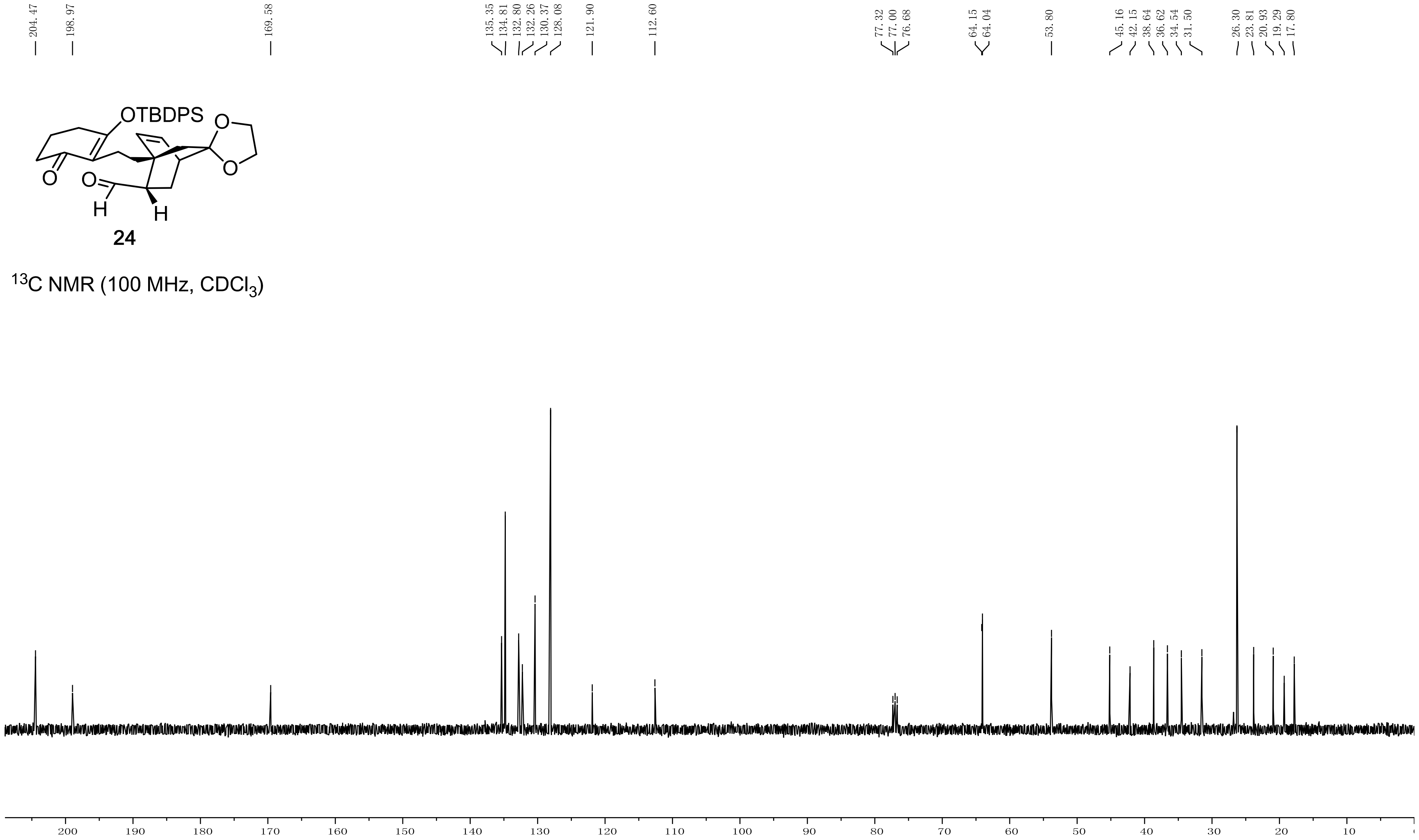

Supplementary Figure 23. ^{13}C NMR spectrum of 24

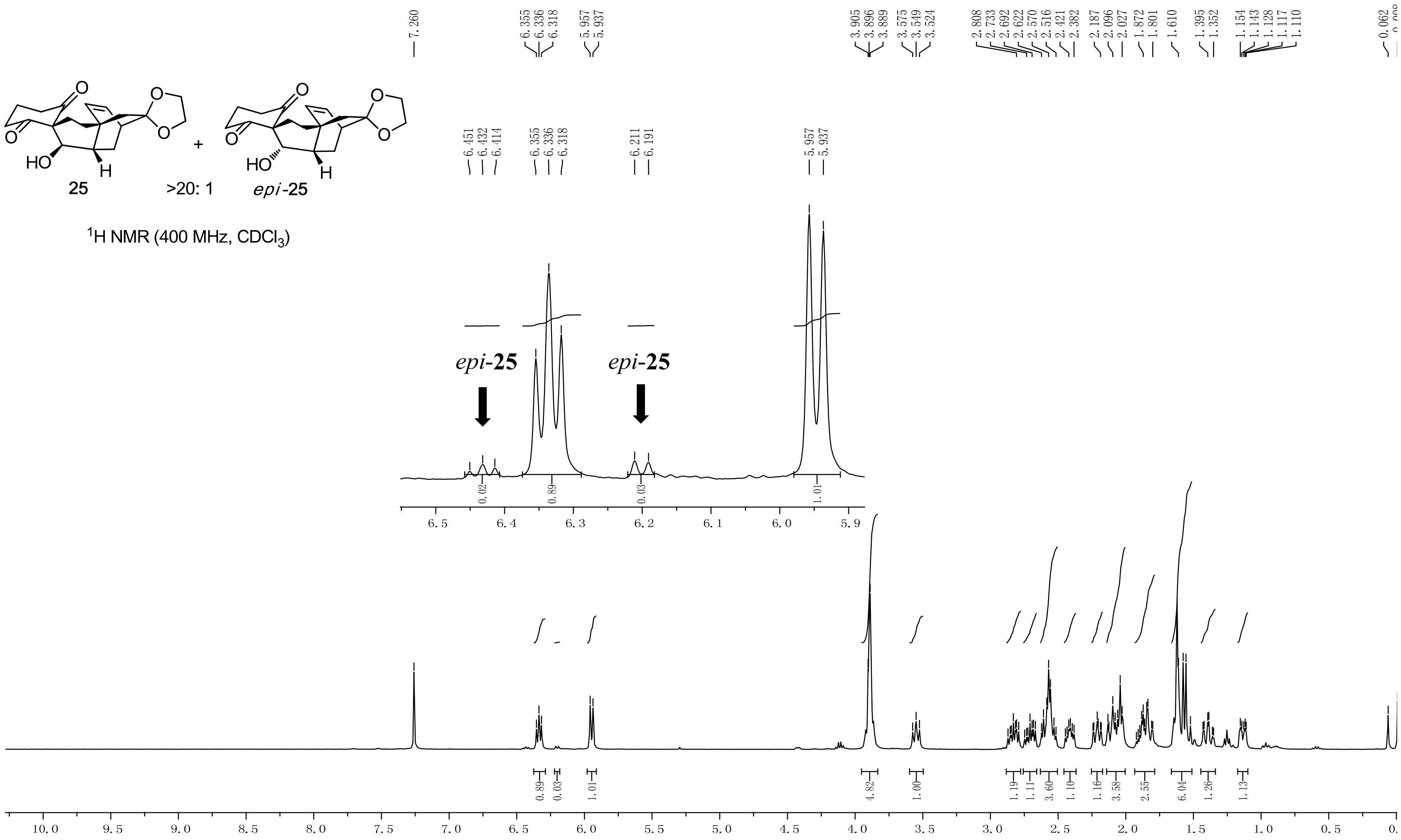

Supplementary Figure 24. ^{1}H NMR spectrum of 25

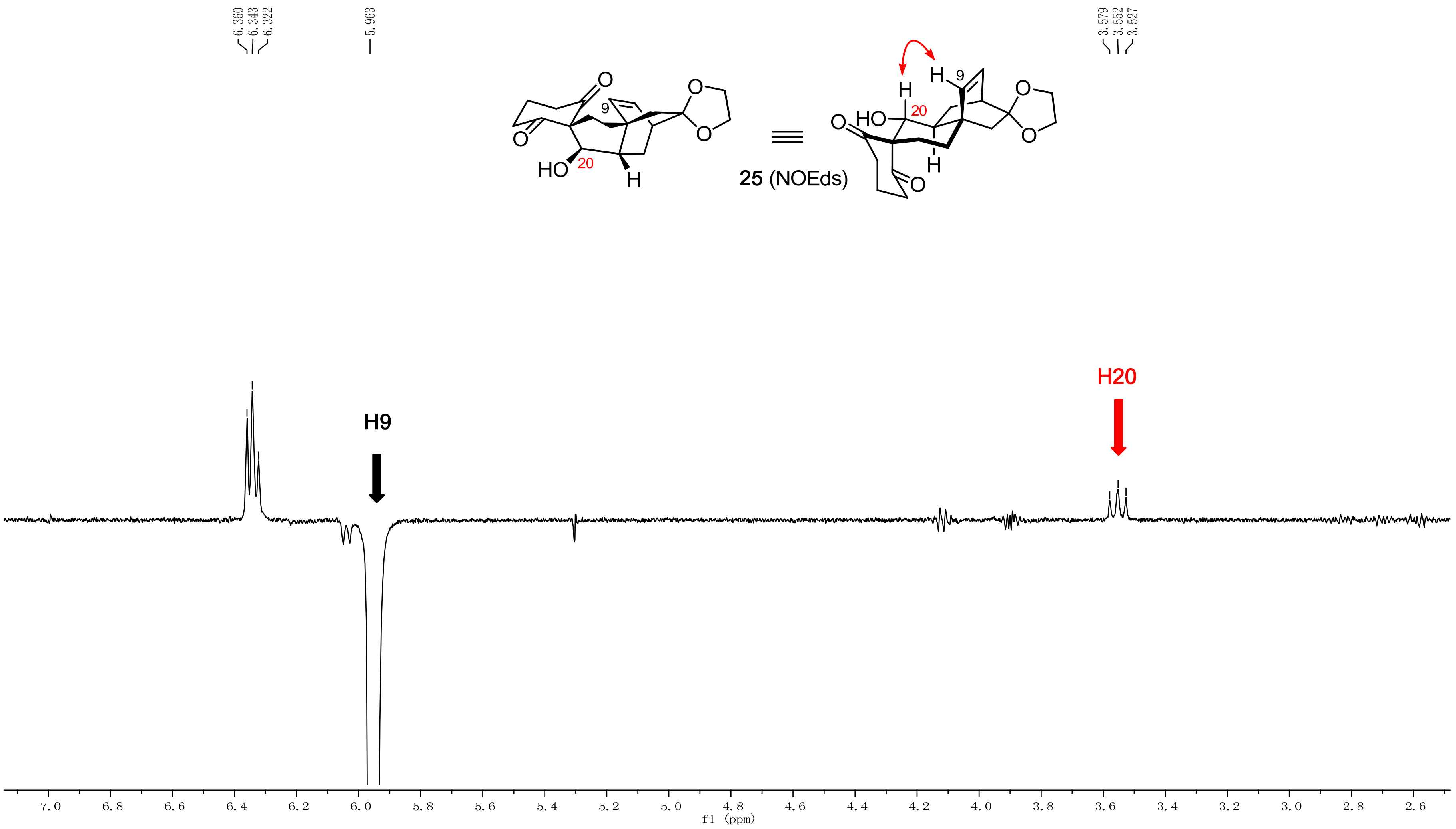

Supplementary Figure 25. NOEds of 25

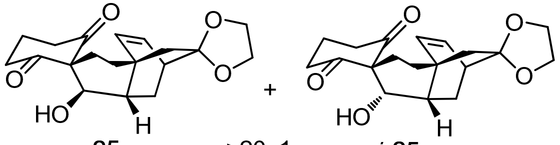

25 >20: 1 *epi*-25

^{13}C NMR (100 MHz, $CDCl_3$)

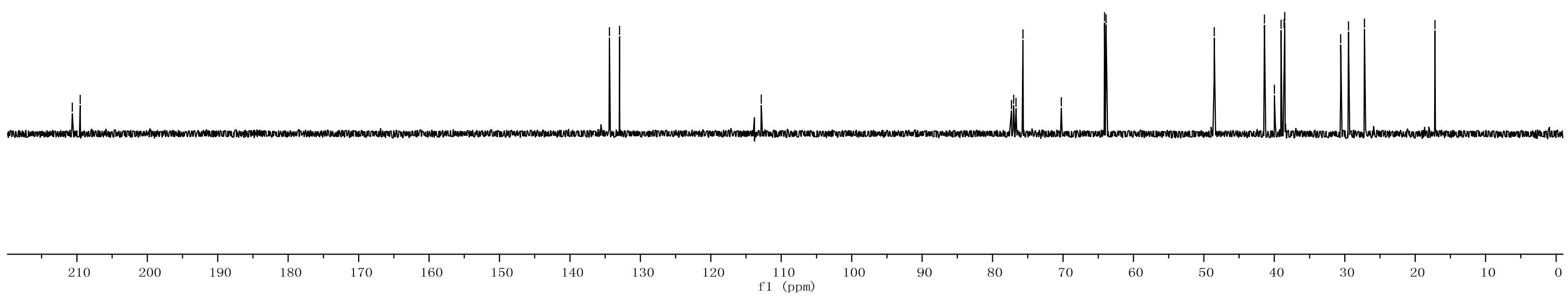

Supplementary Figure 26. ^{13}C NMR spectrum of 25

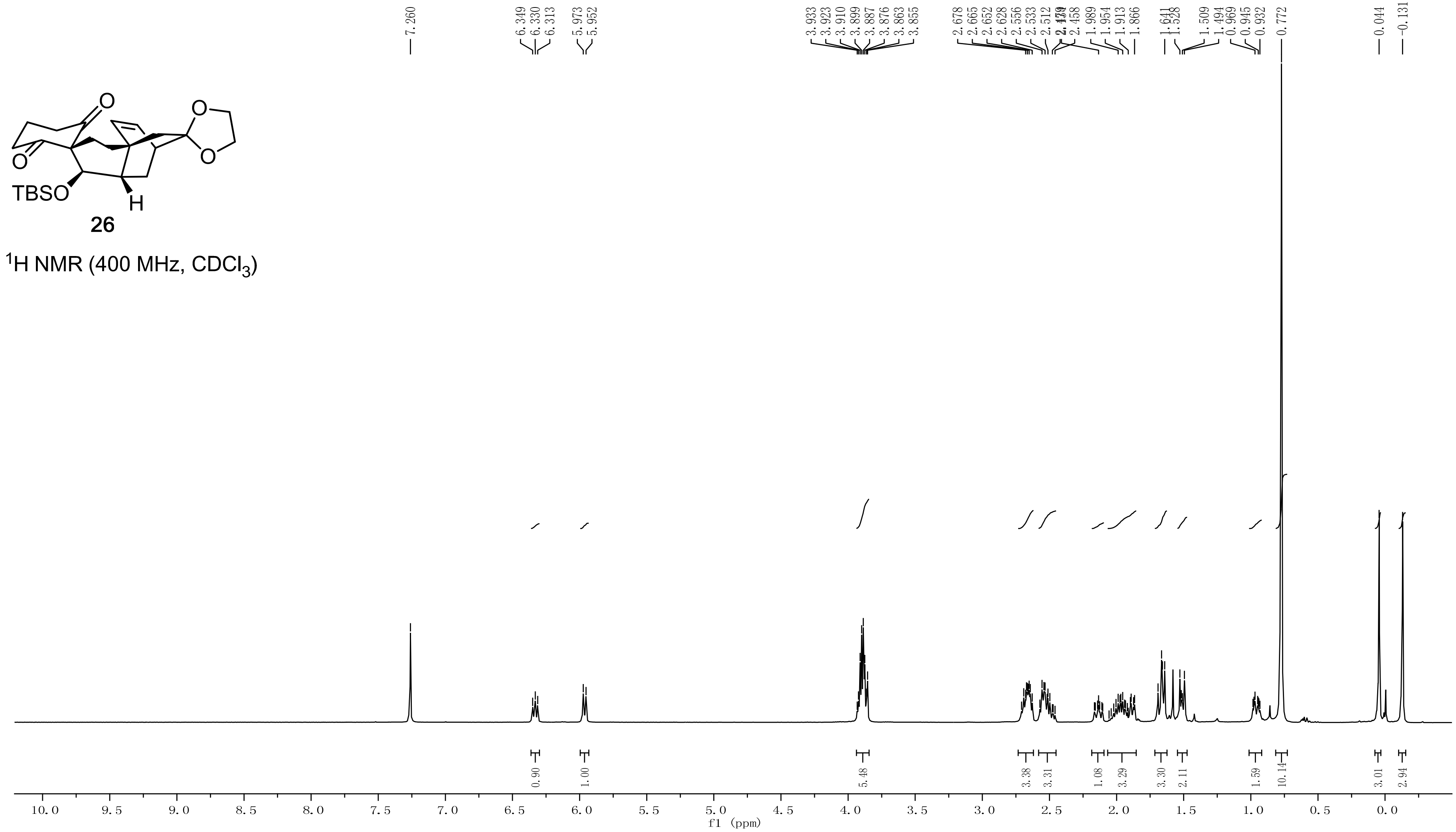

Supplementary Figure 27. ^{1}H NMR spectrum of 26

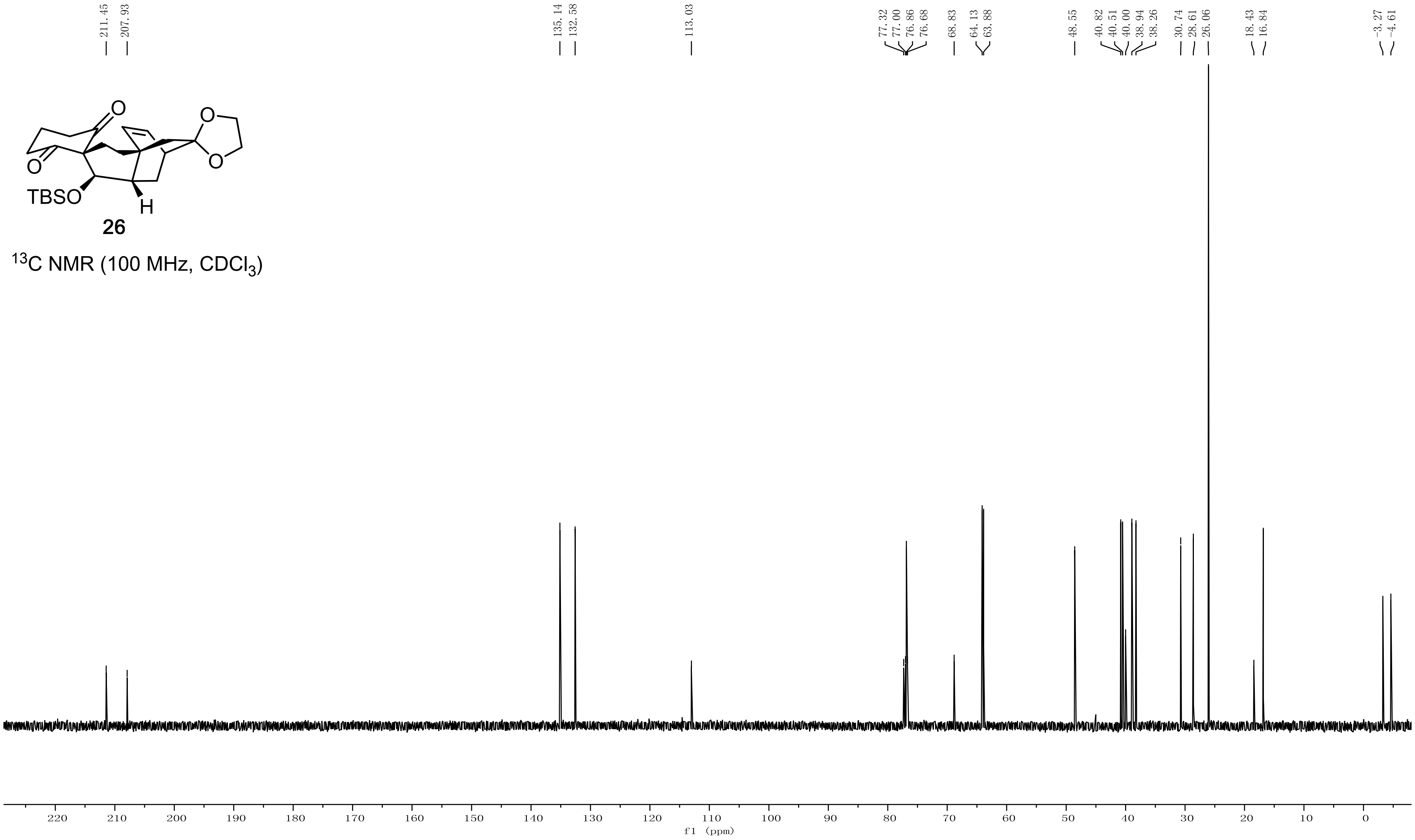

Supplementary Figure 28. ^{13}C NMR spectrum of 26

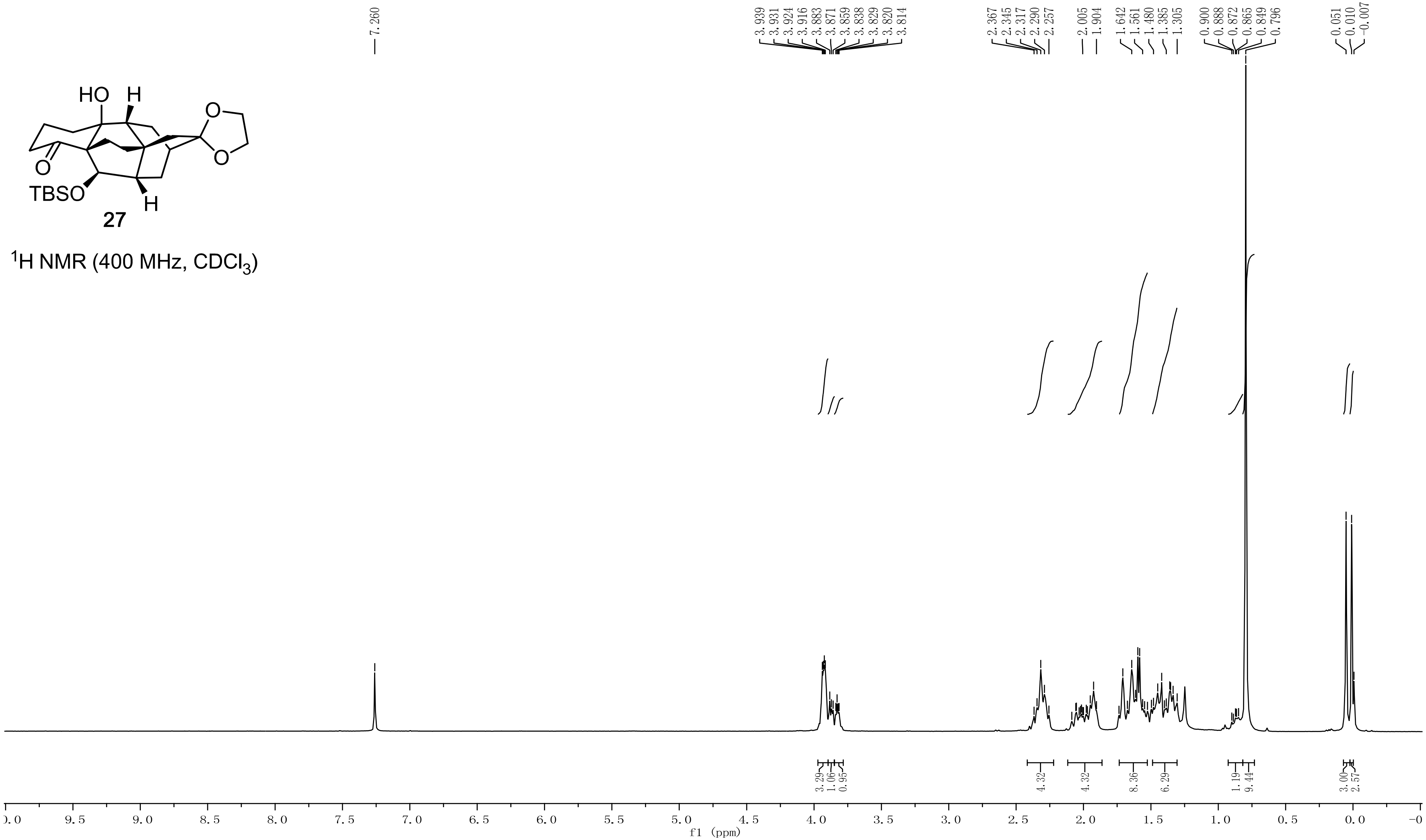

Supplementary Figure 29. ^{1}H NMR spectrum of 27

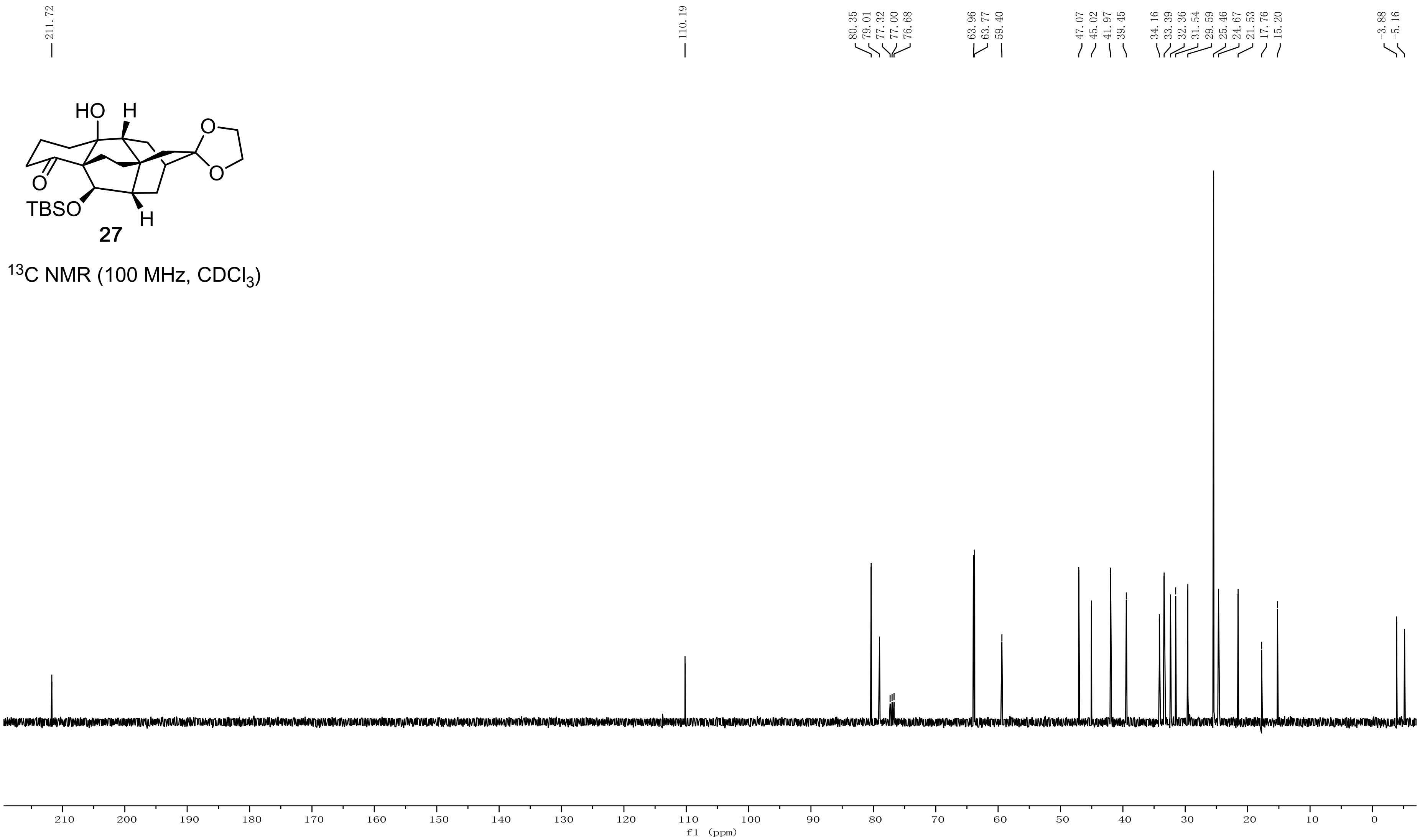

Supplementary Figure 30. ^{13}C NMR spectrum of 27

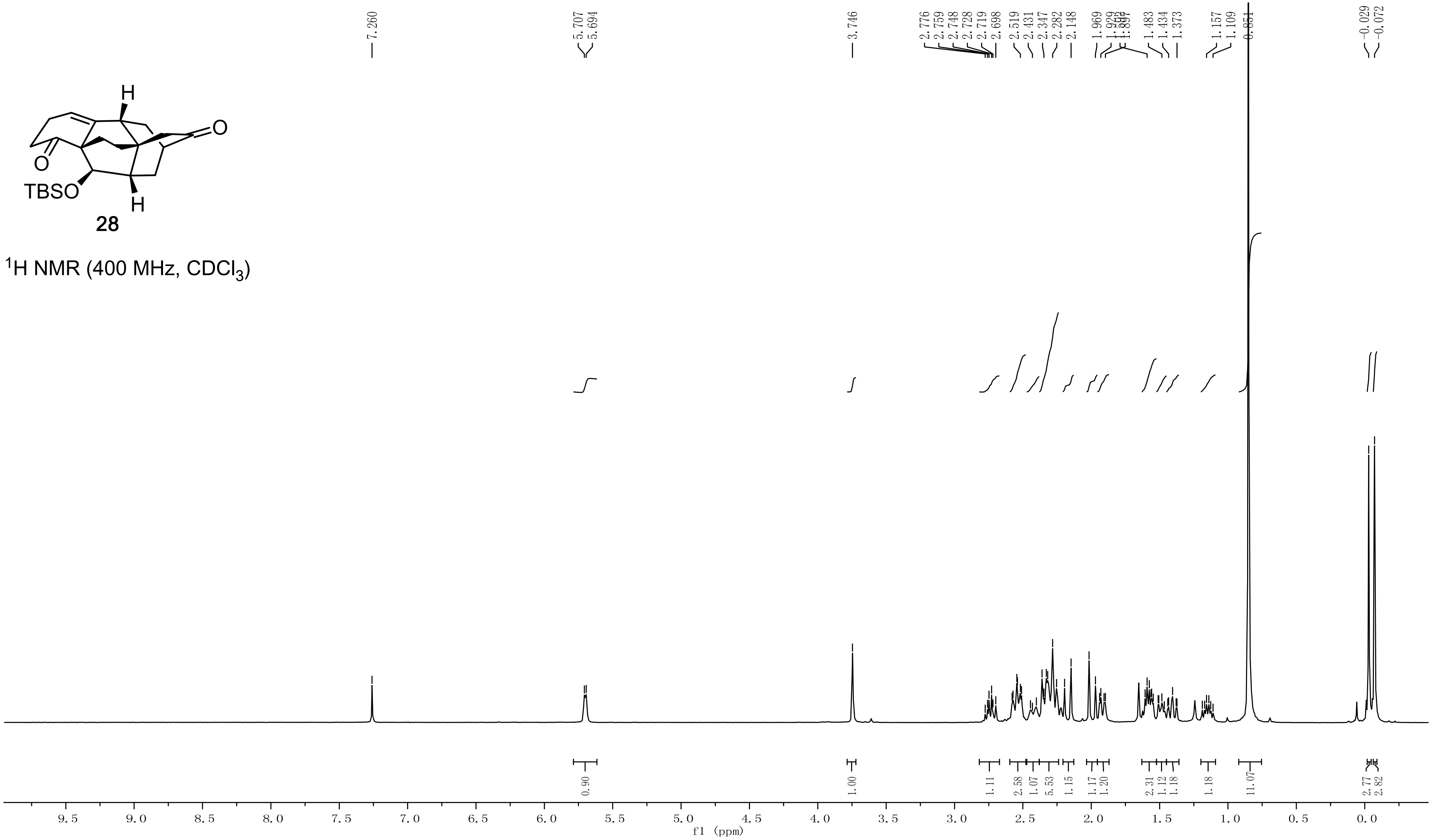

Supplementary Figure 31. ^{1}H NMR spectrum of 28

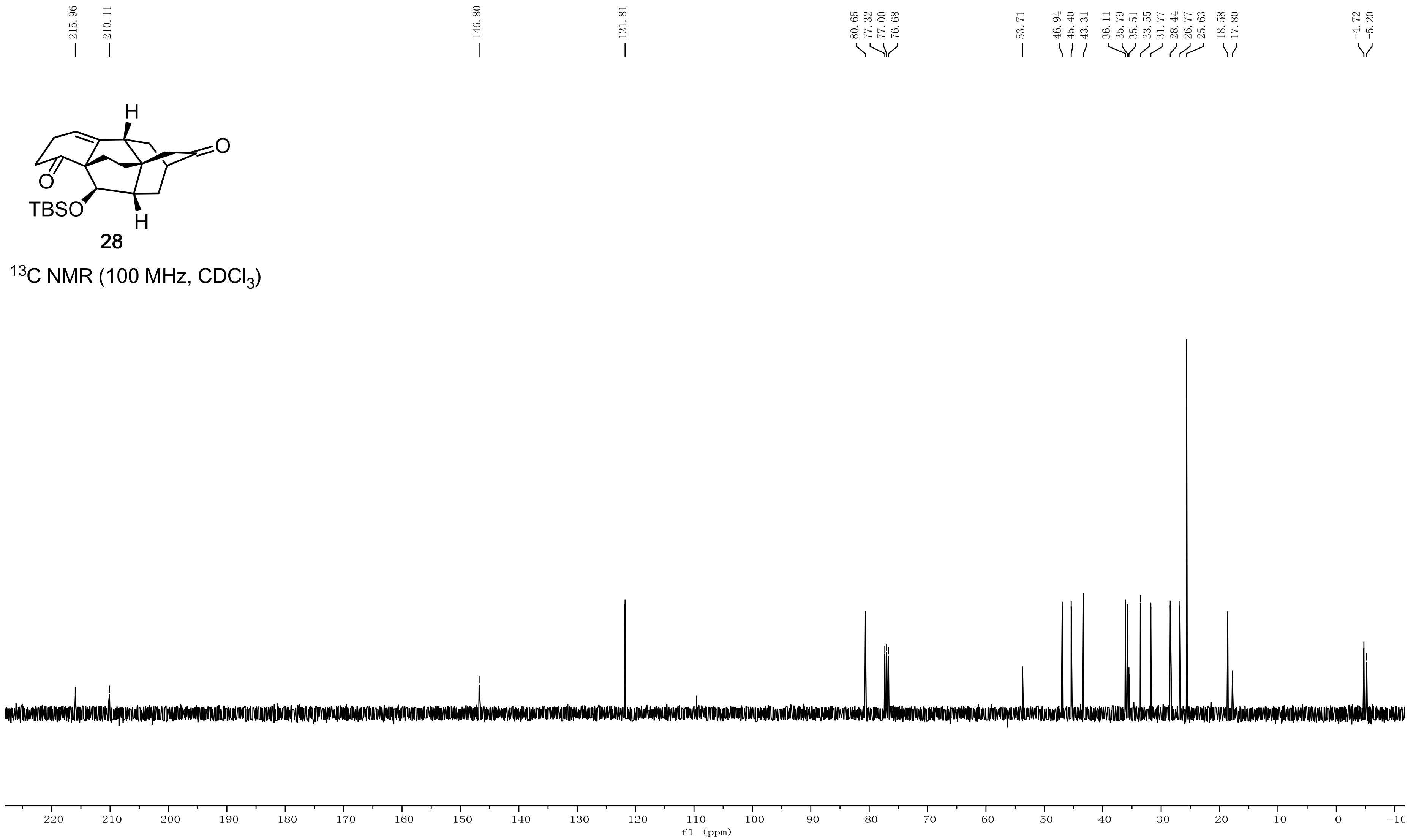

Supplementary Figure 32. ^{13}C NMR spectrum of 28

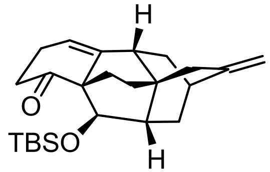
S4

^{1}H NMR (400 MHz, $CDCl_3$)

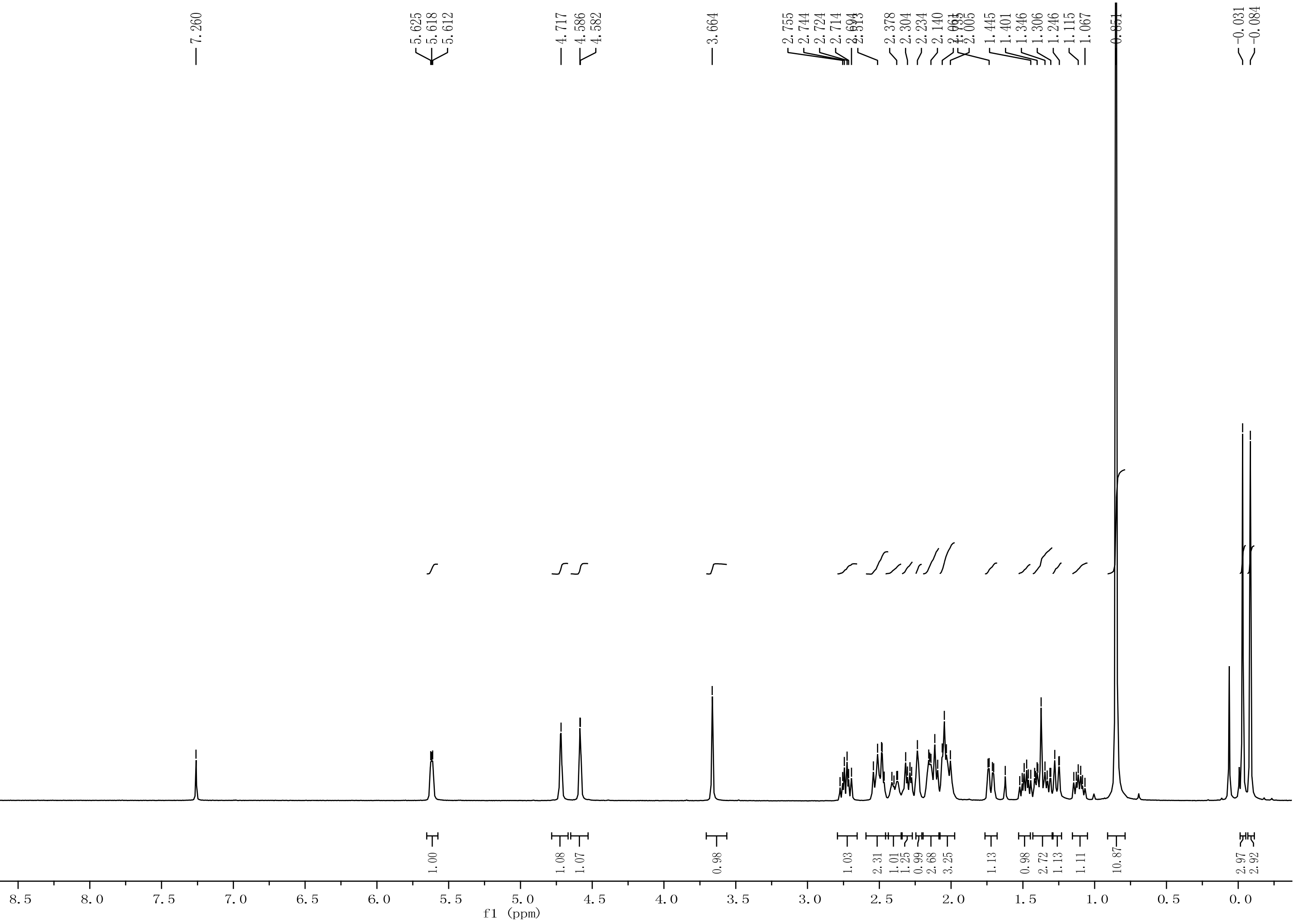

Supplementary Figure 33. ^{1}H NMR spectrum of S4

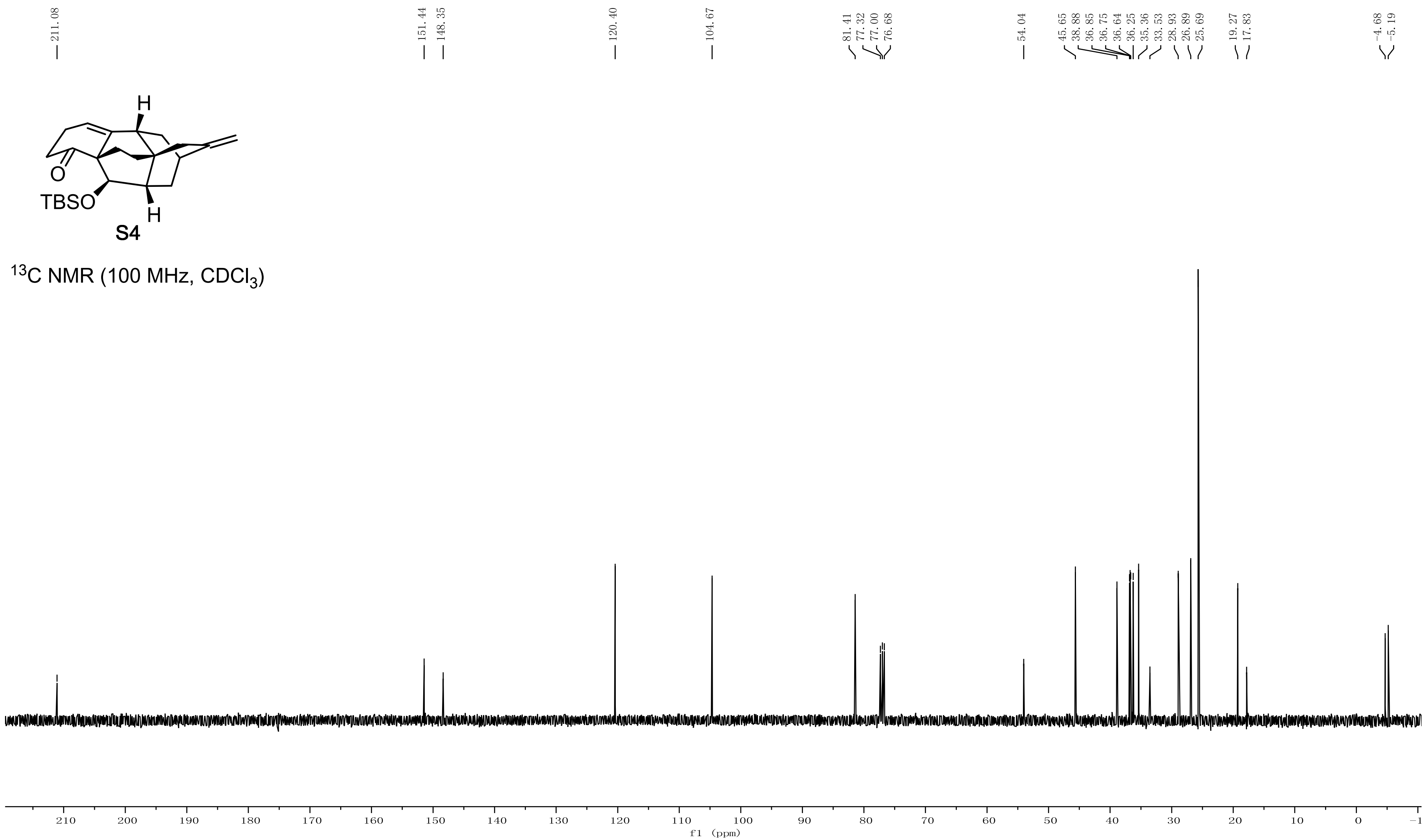

Supplementary Figure 34. ^{13}C NMR spectrum of S4

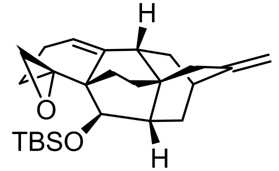

S5

^{1}H NMR (400 MHz, $CDCl_3$)

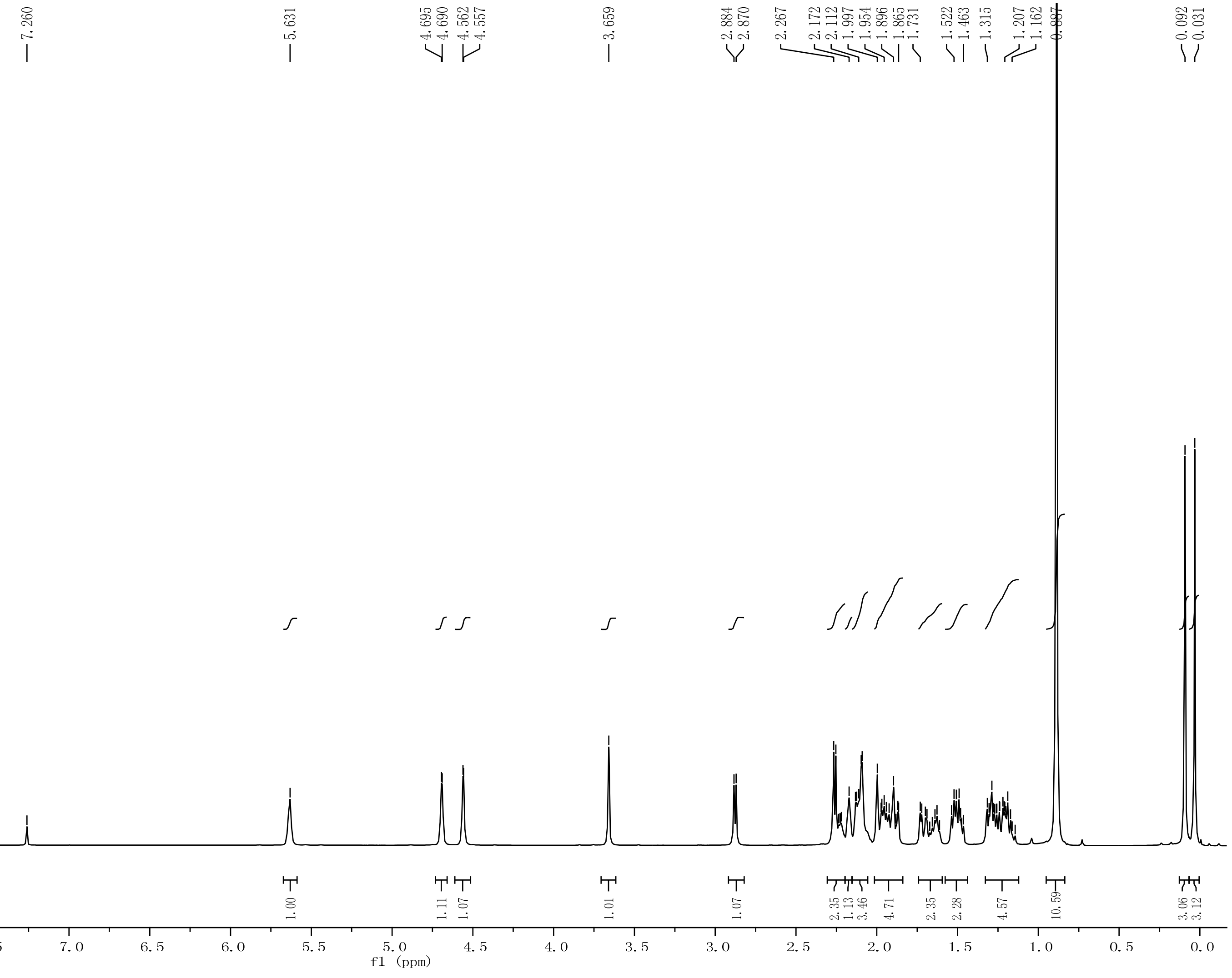

Supplementary Figure 35. ^{1}H NMR spectrum of S5

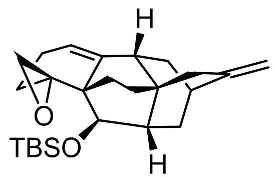

^{13}C NMR (100 MHz, $CDCl_3$)

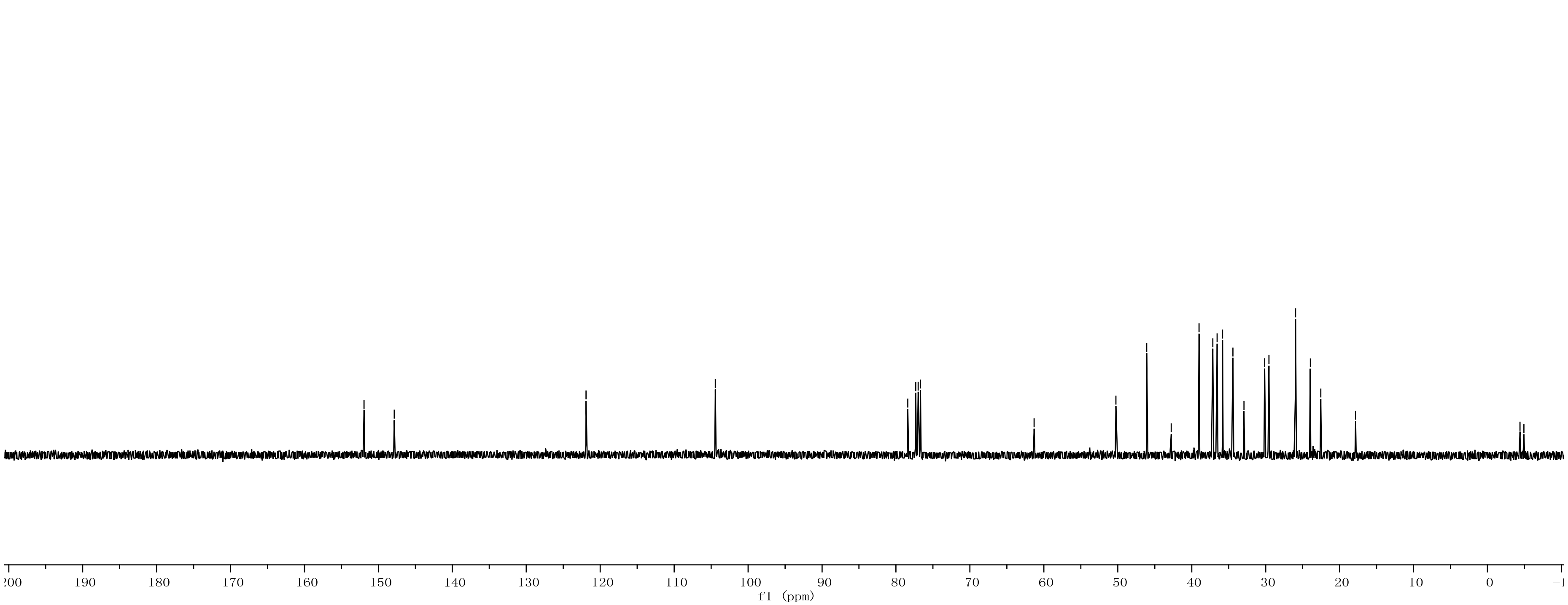

Supplementary Figure 36. ^{13}C NMR spectrum of S5

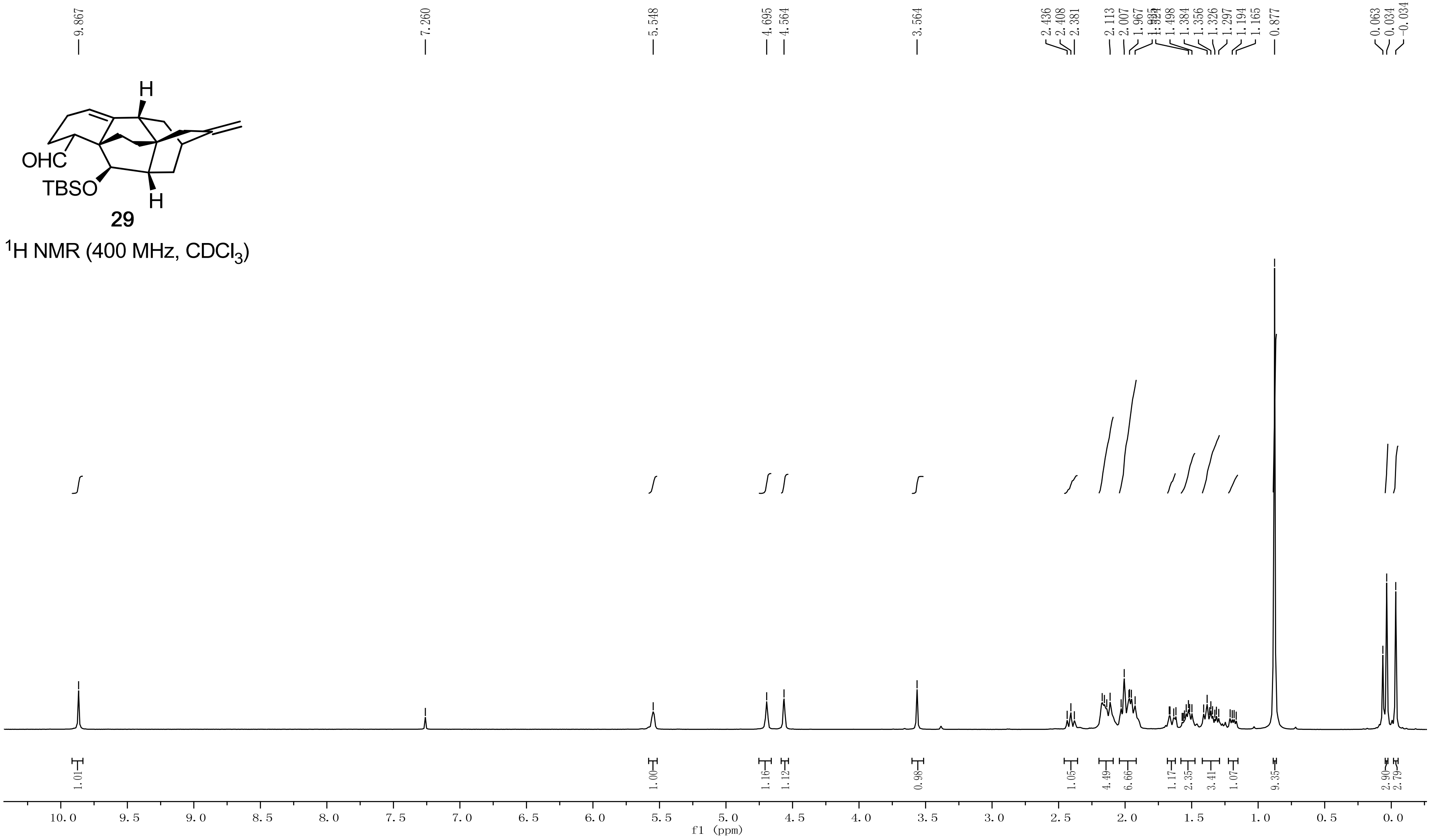

Supplementary Figure 37. ^{1}H NMR spectrum of 29

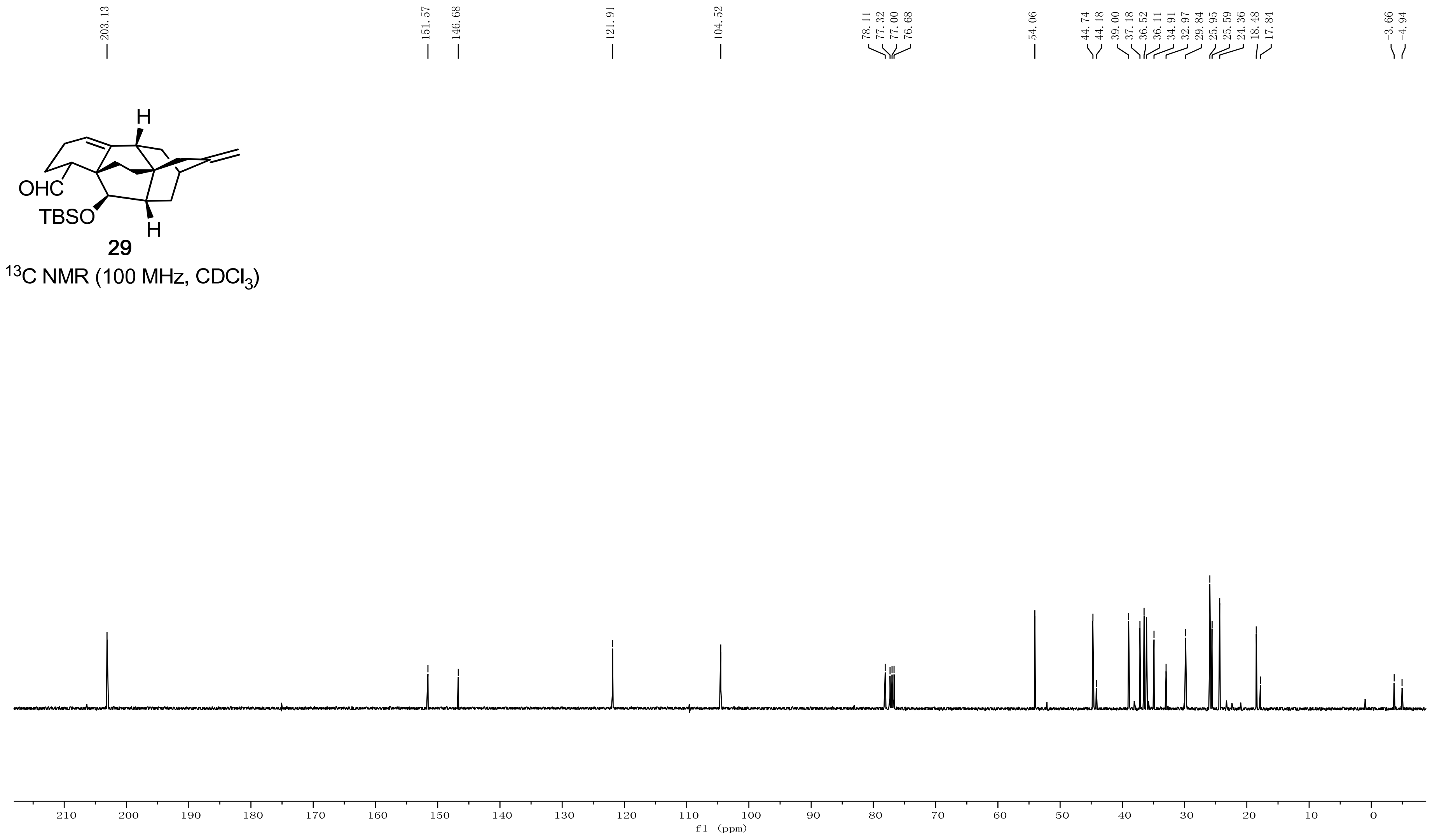

Supplementary Figure 38. ^{13}C NMR spectrum of 29

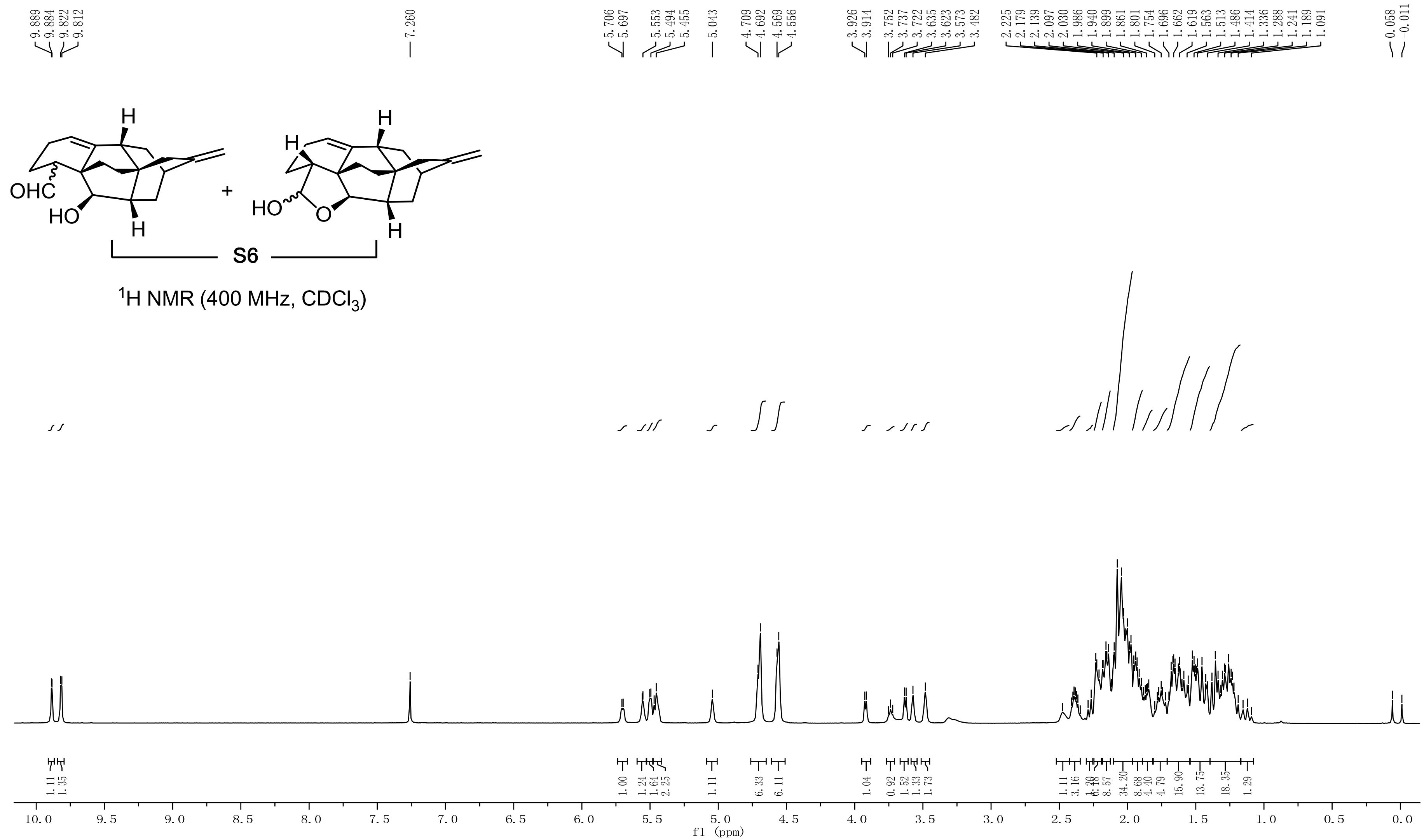

Supplementary Figure 39. ^{1}H NMR spectrum of S6

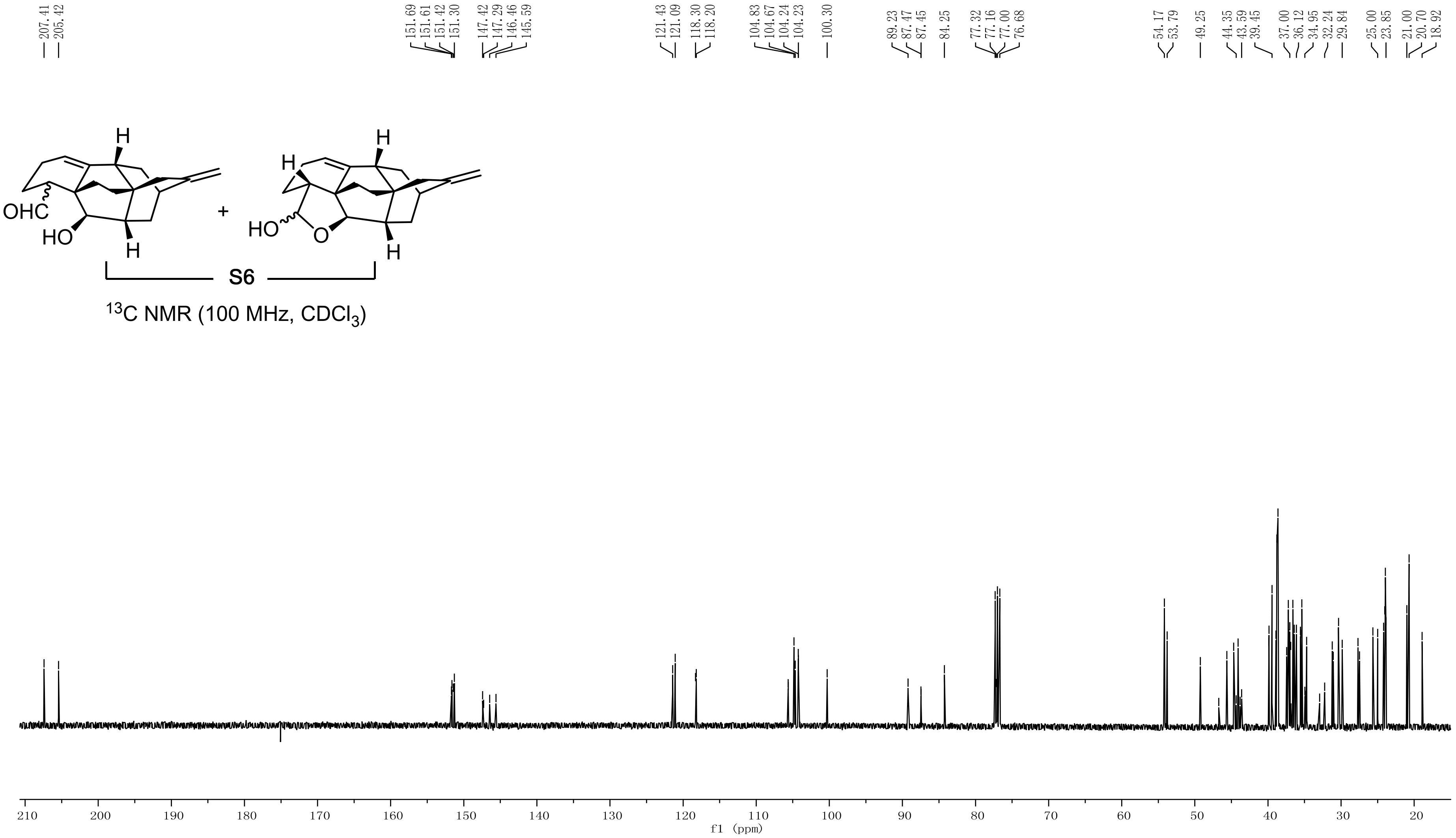

Supplementary Figure 40. ^{13}C NMR spectrum of S6

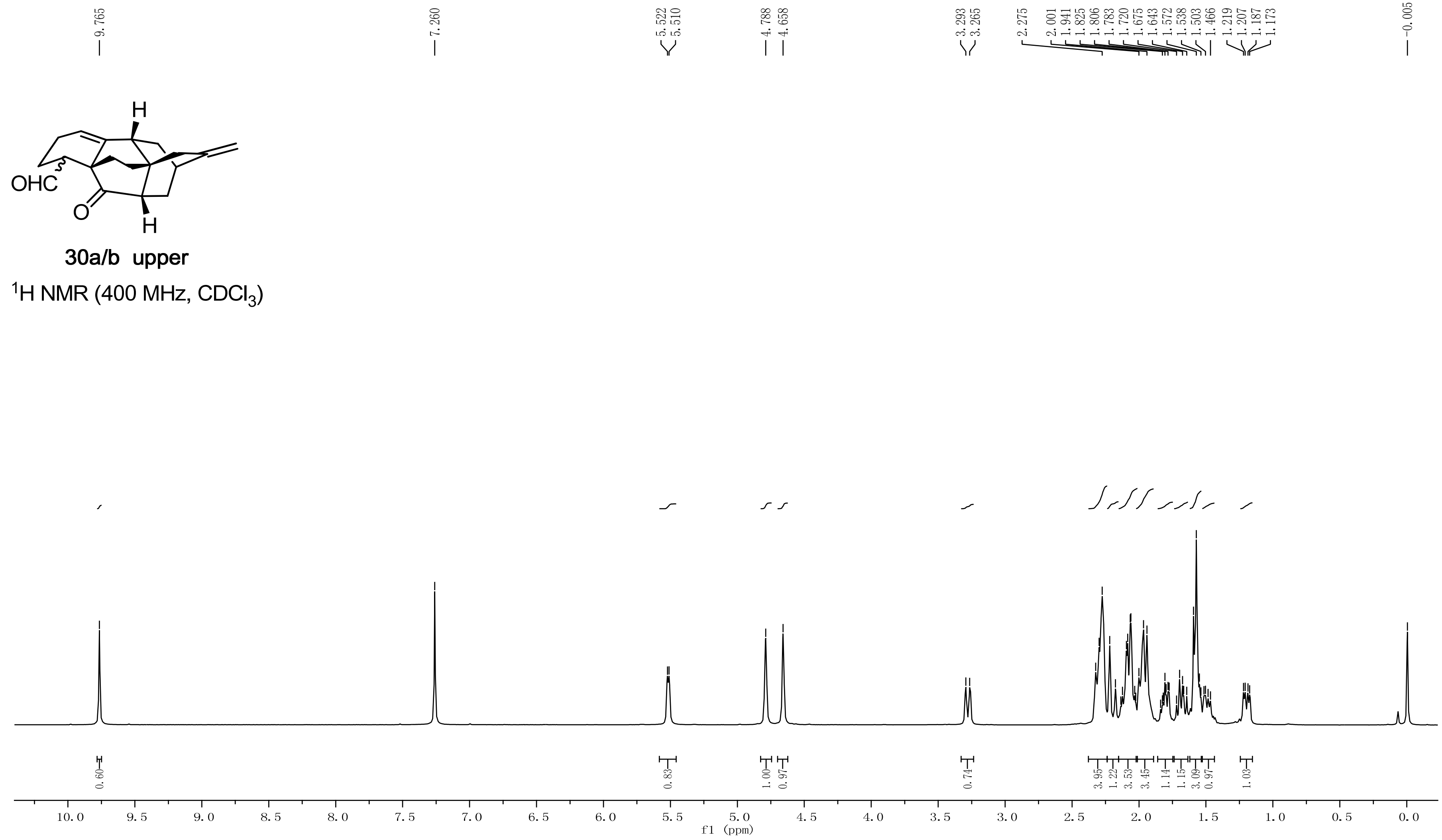

Supplementary Figure 41. ^{1}H NMR spectrum of 30a/b upper

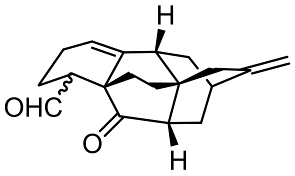

30a/b upper

^{1}H NMR (100 MHz, $CDCl_3$)

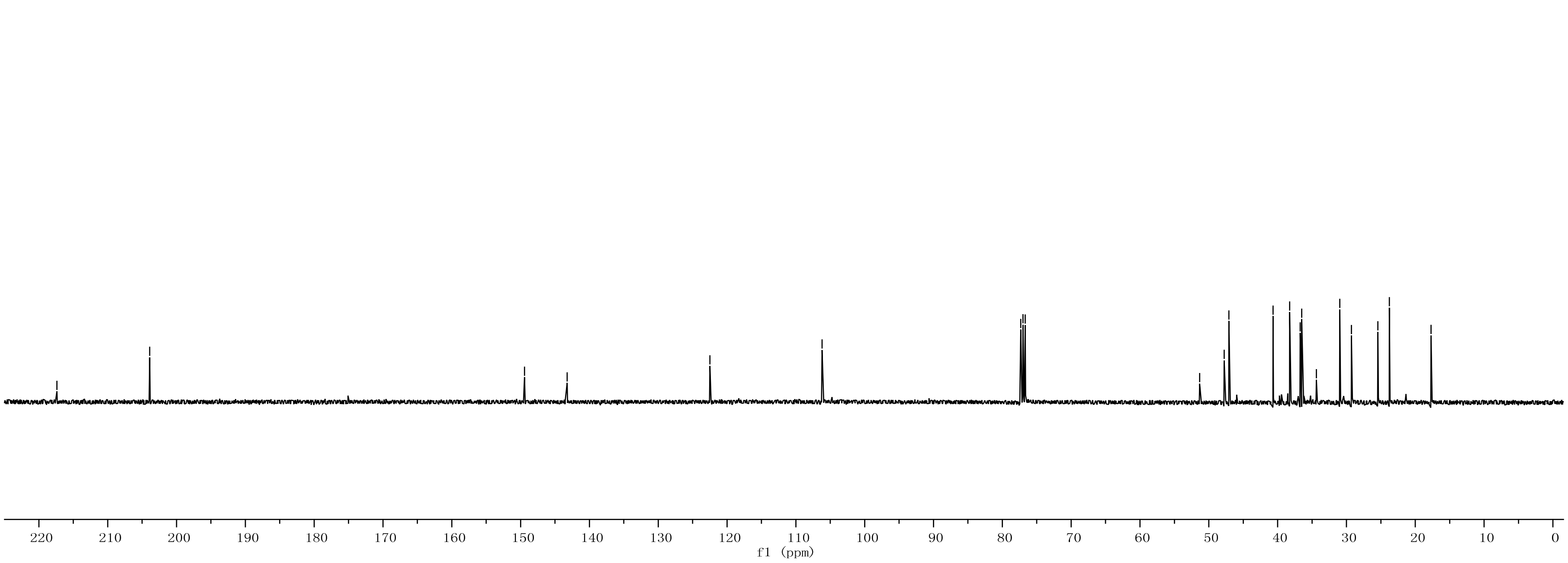

Supplementary Figure 42. ^{13}C NMR spectrum of 30a/b upper

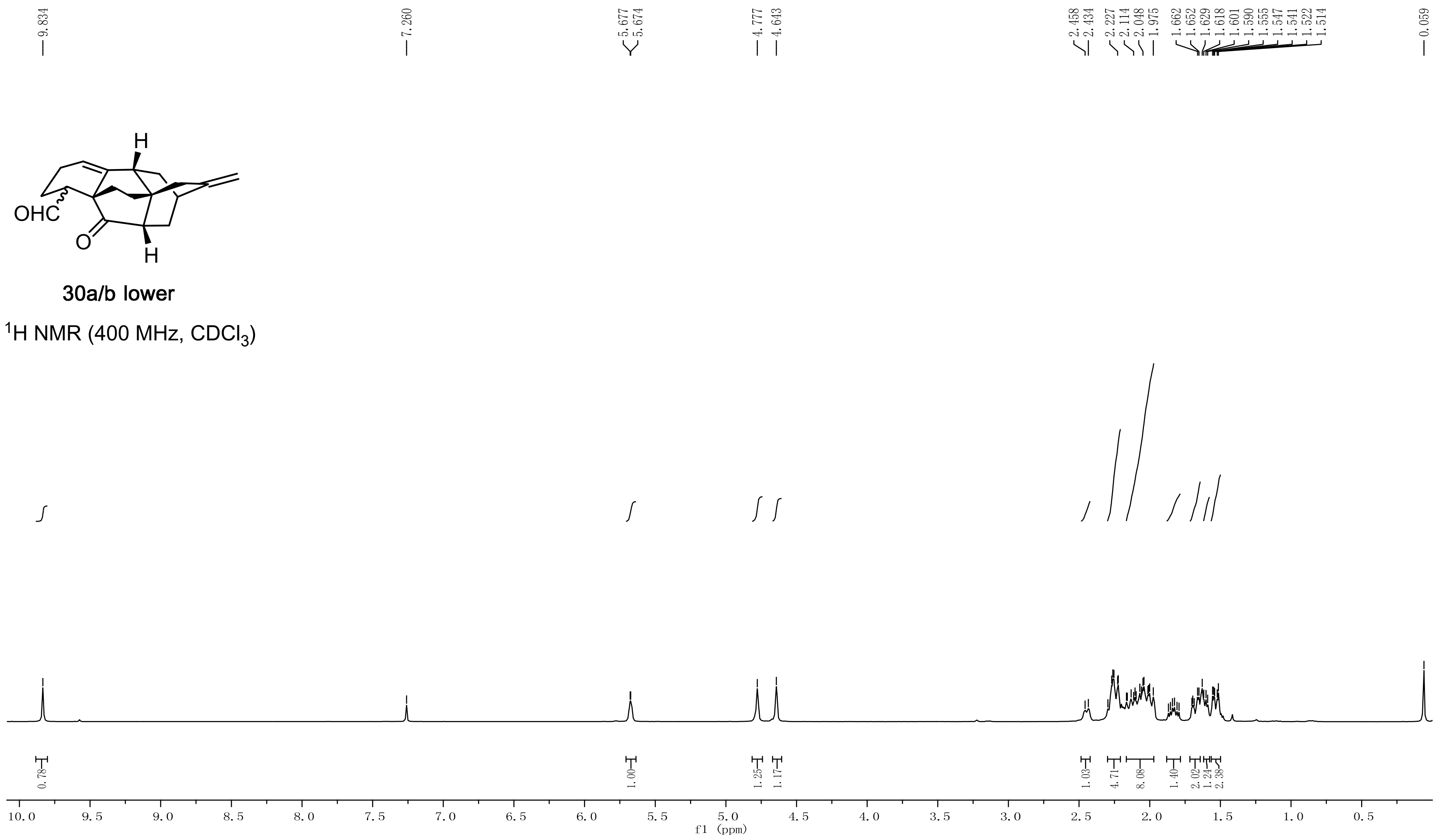

Supplementary Figure 43. ^{1}H NMR spectrum of 30a/b lower

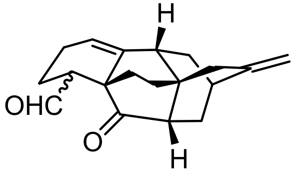

30a/b lower

^{1}H NMR (100 MHz, $CDCl_3$)

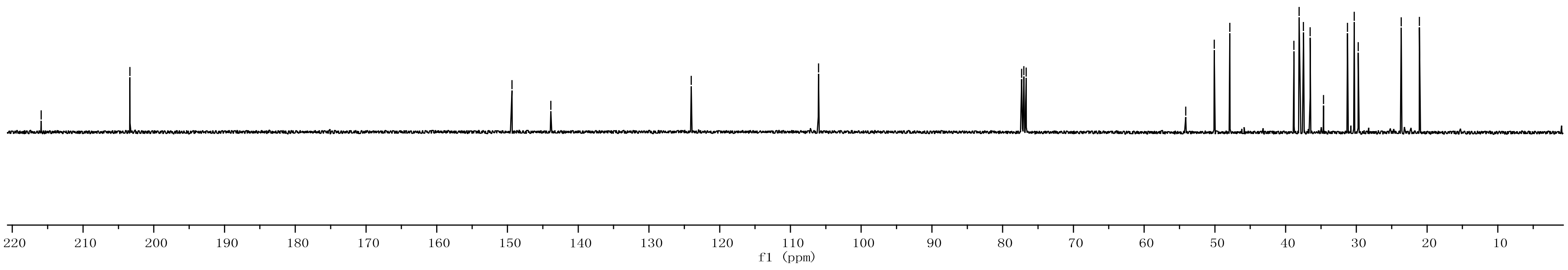

Supplementary Figure 44. ^{13}C NMR spectrum of 30a/b lower

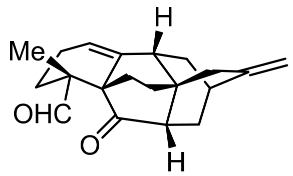

31

^{1}H NMR (400 MHz, $CDCl_3$)

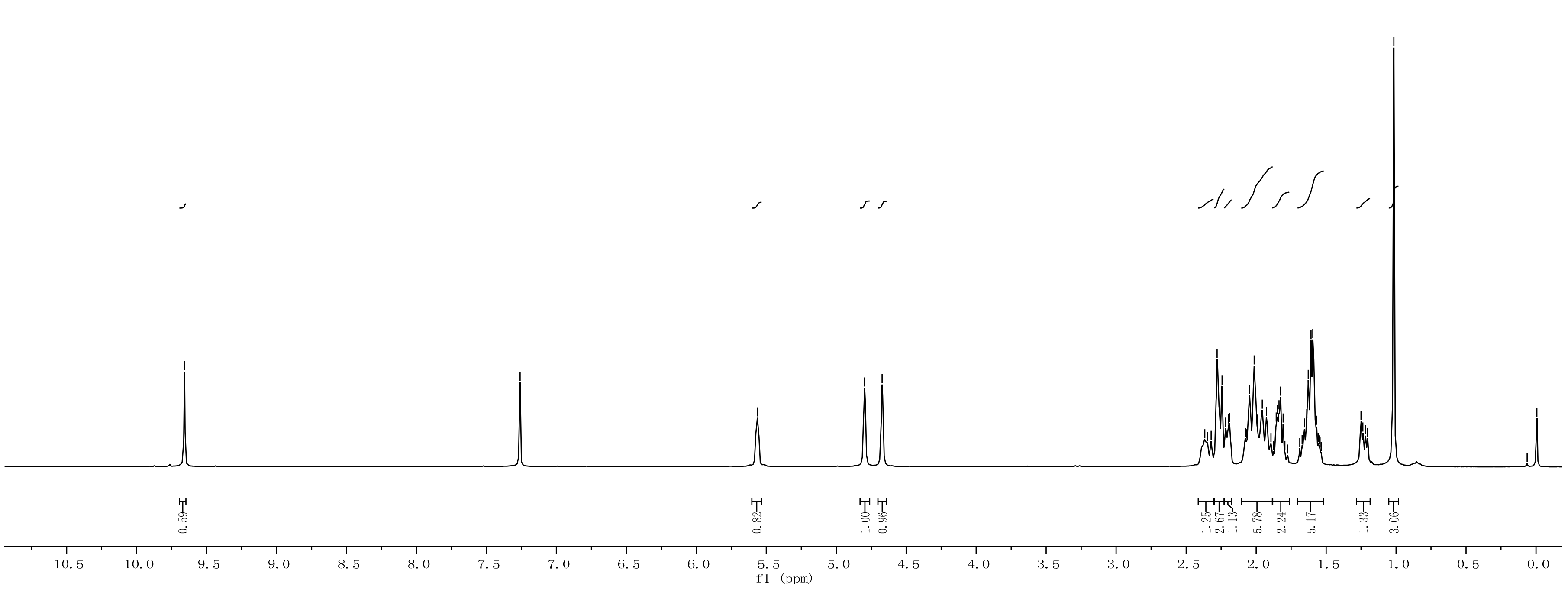

Supplementary Figure 45. ^{1}H NMR spectrum of 31

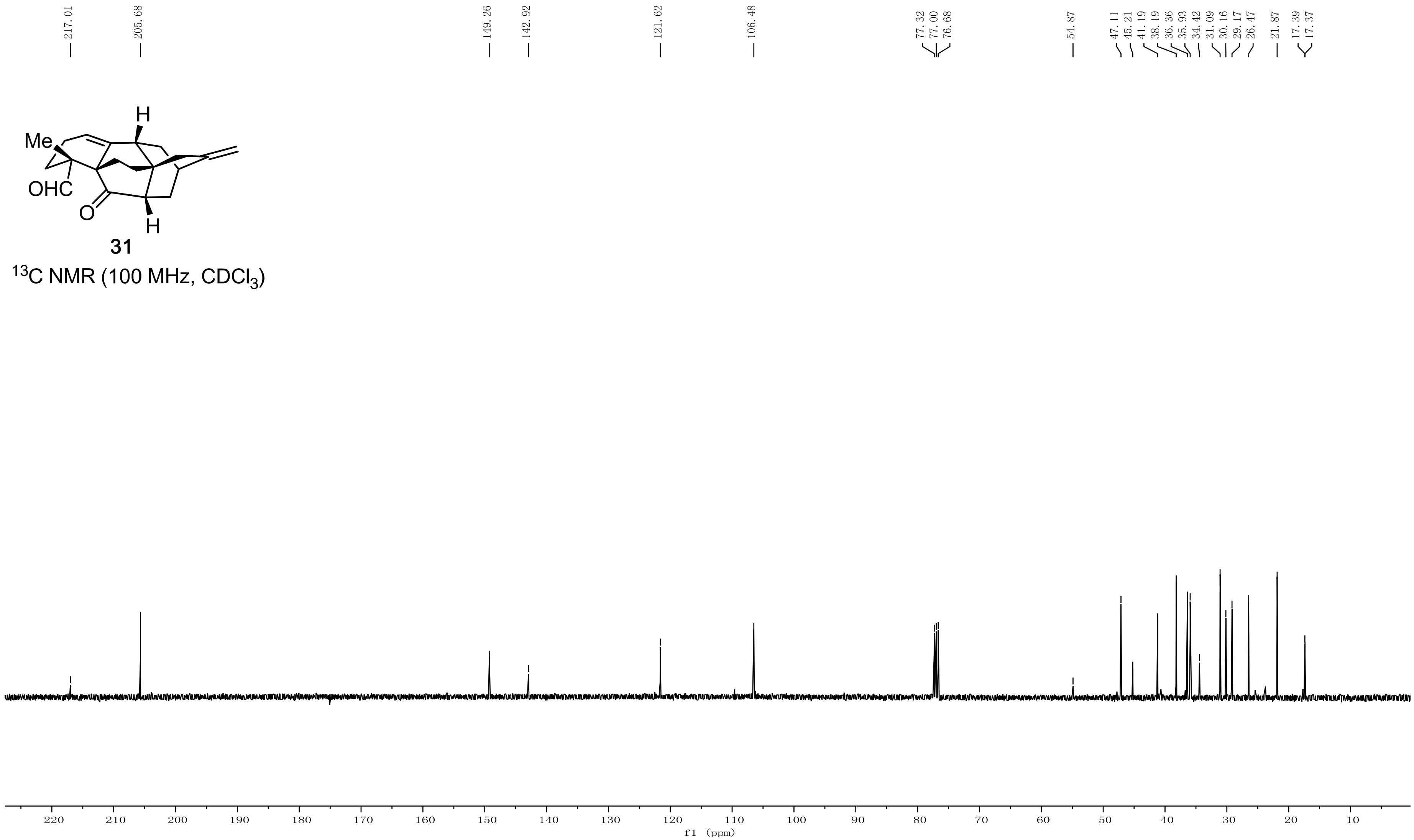

Supplementary Figure 46. ^{13}C NMR spectrum of 31

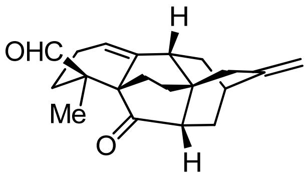

S7

^{1}H NMR (600 MHz, $CDCl_3$)

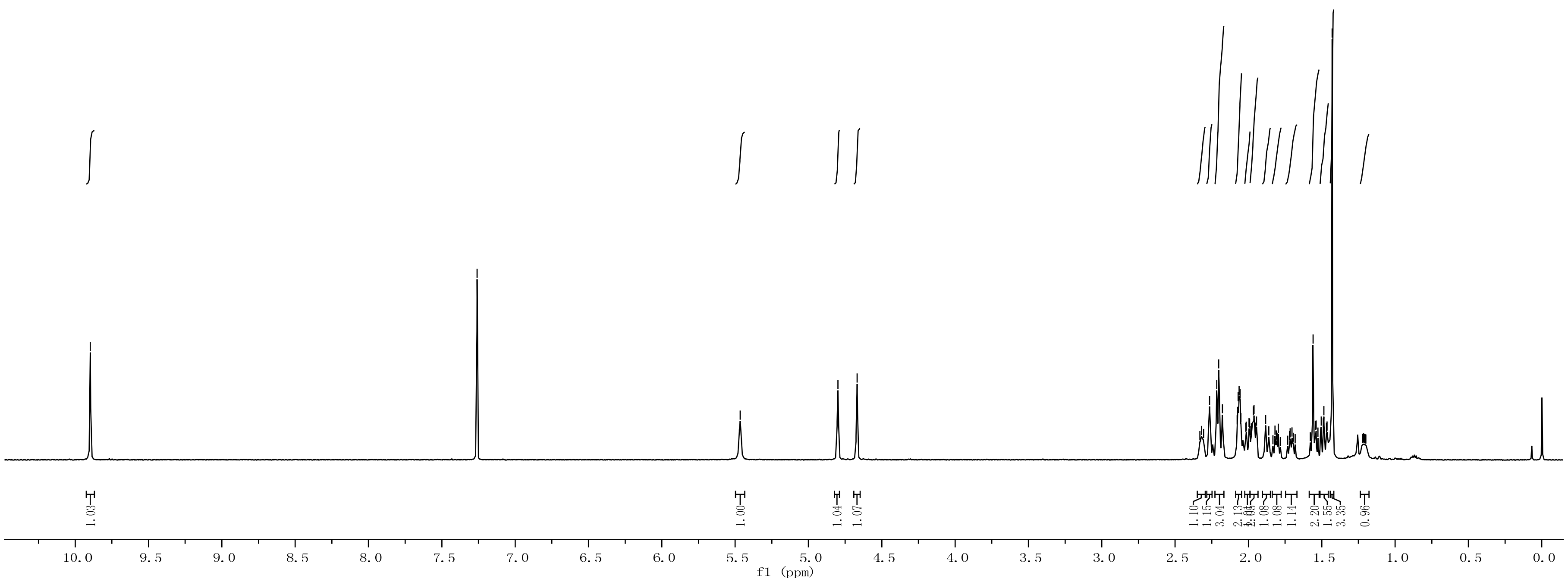

Supplementary Figure 47. ^{1}H NMR spectrum of S7

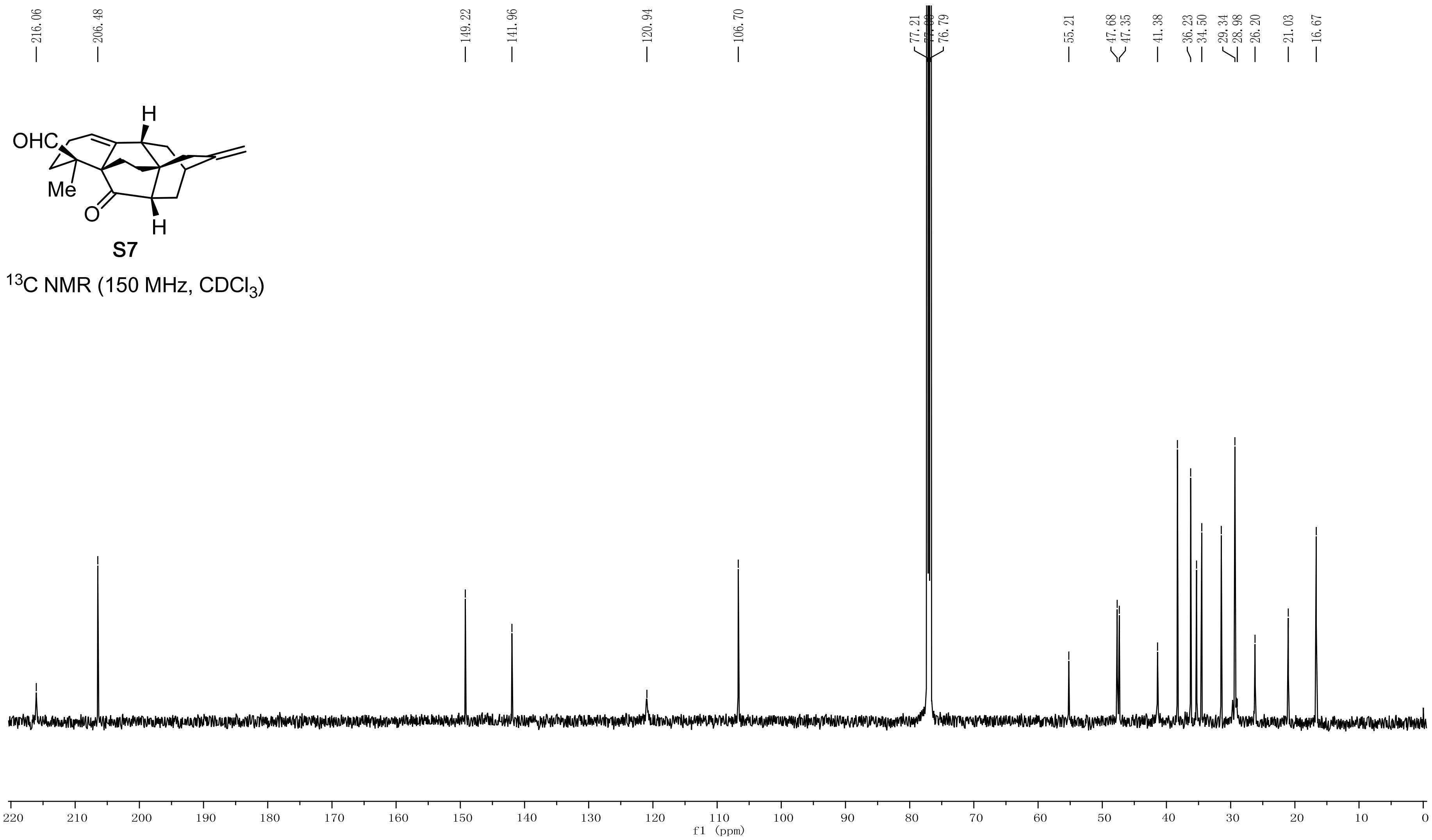

Supplementary Figure 48. ^{13}C NMR spectrum of S7

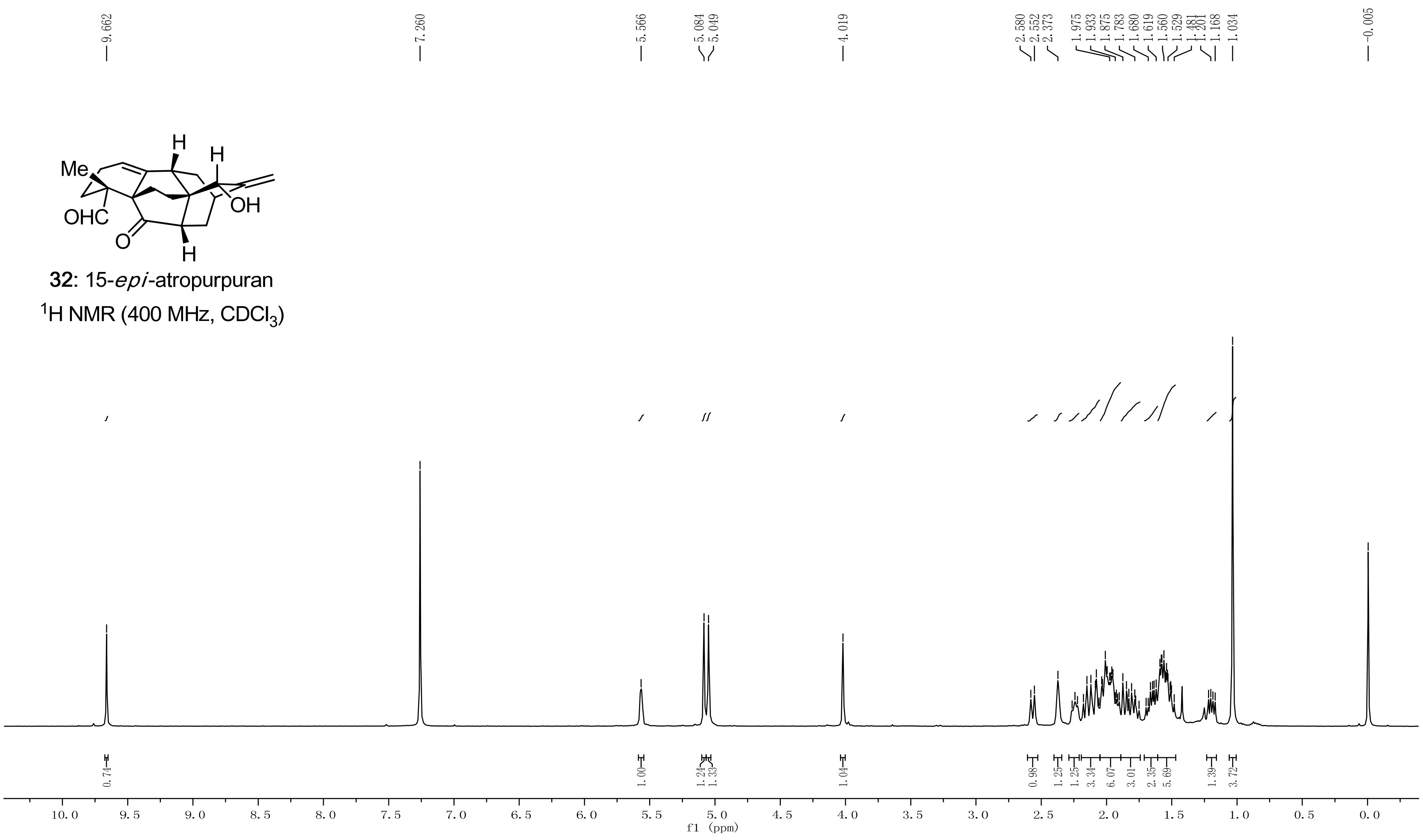

Supplementary Figure 49. ¹H NMR spectrum of 32

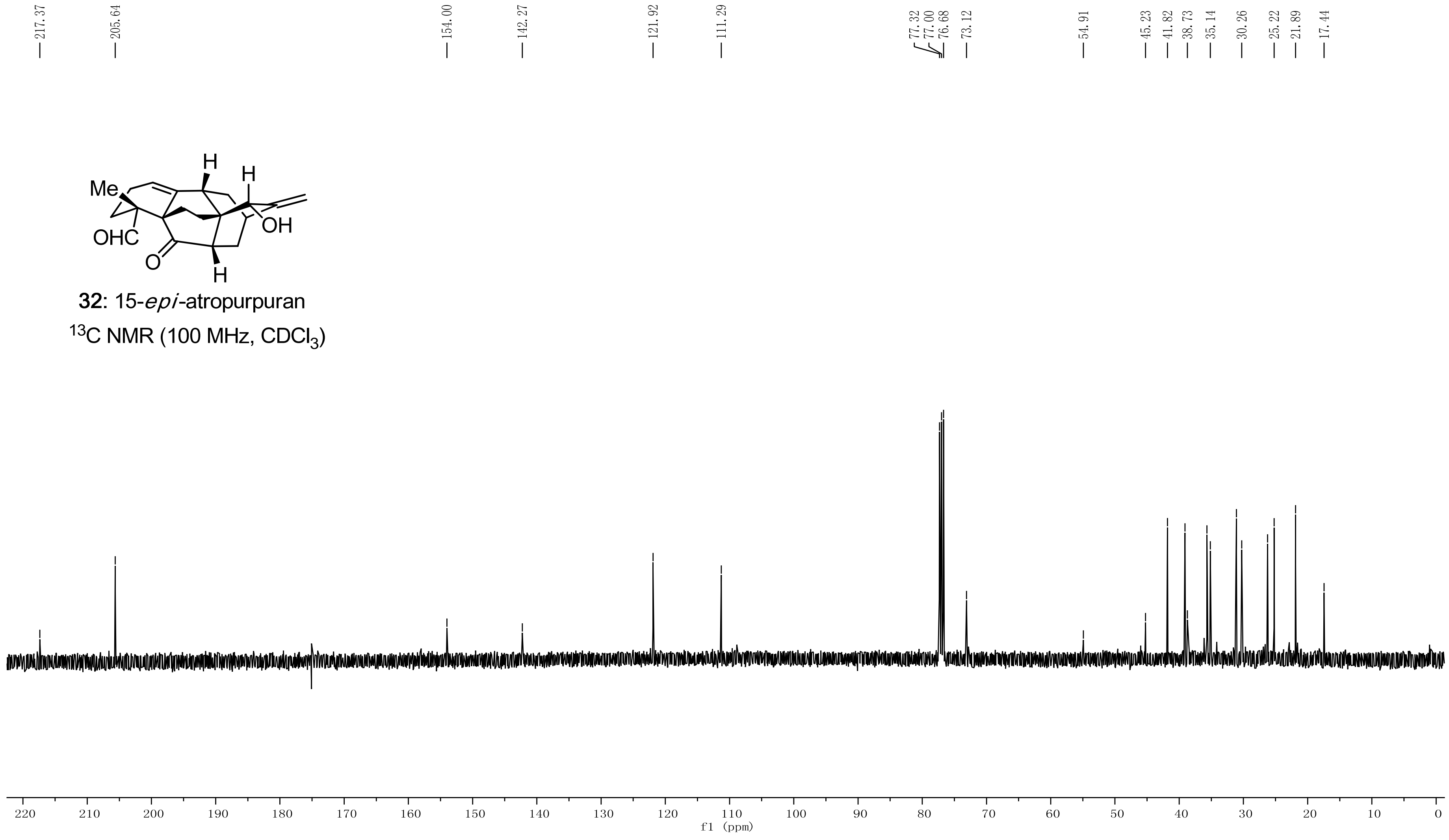

Supplementary Figure 50. ^{13}C NMR spectrum of 32

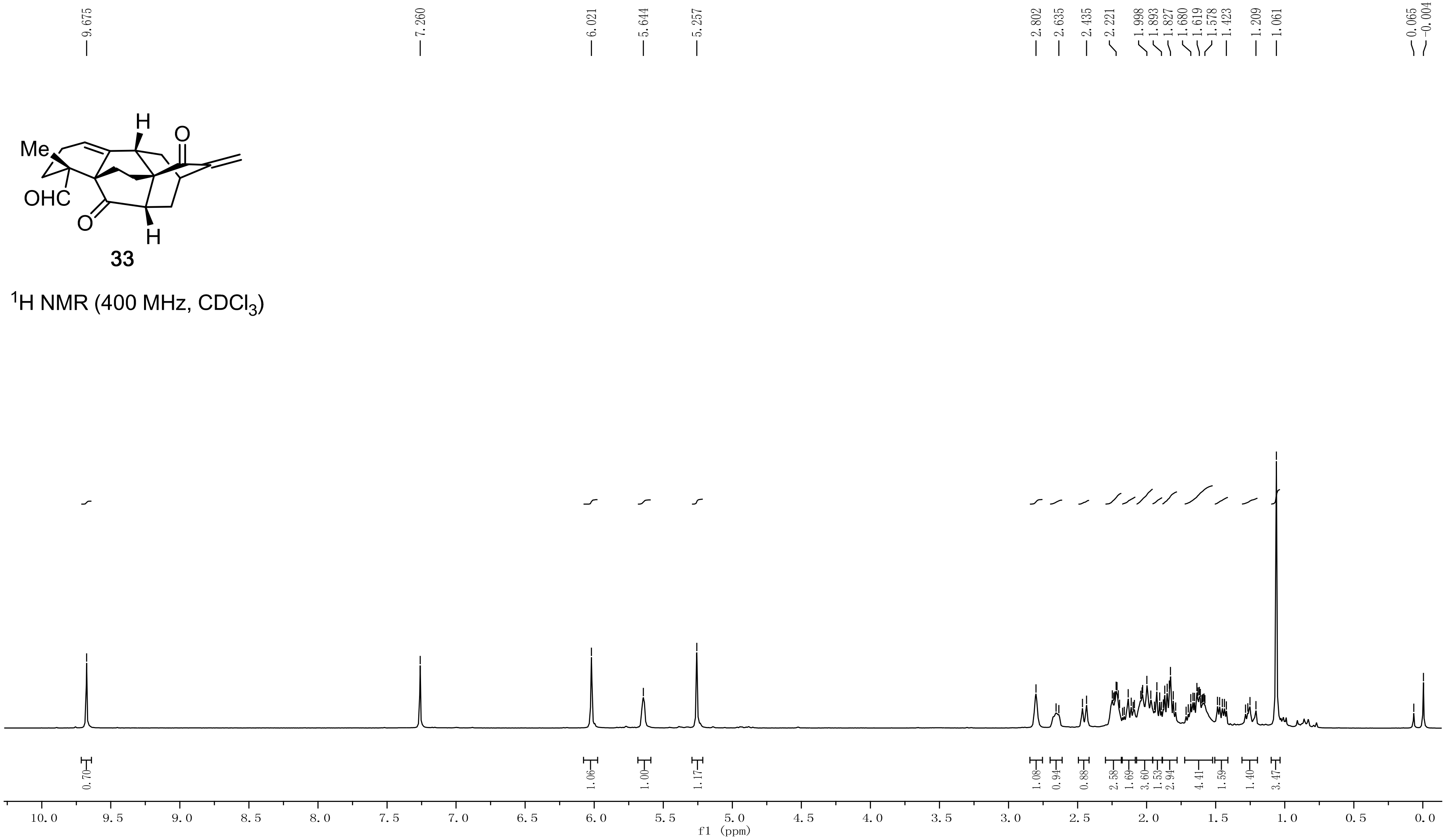

Supplementary Figure 51. ^{1}H NMR spectrum of 33

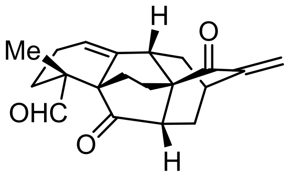

33

^{13}C NMR (100 MHz, $CDCl_3$)

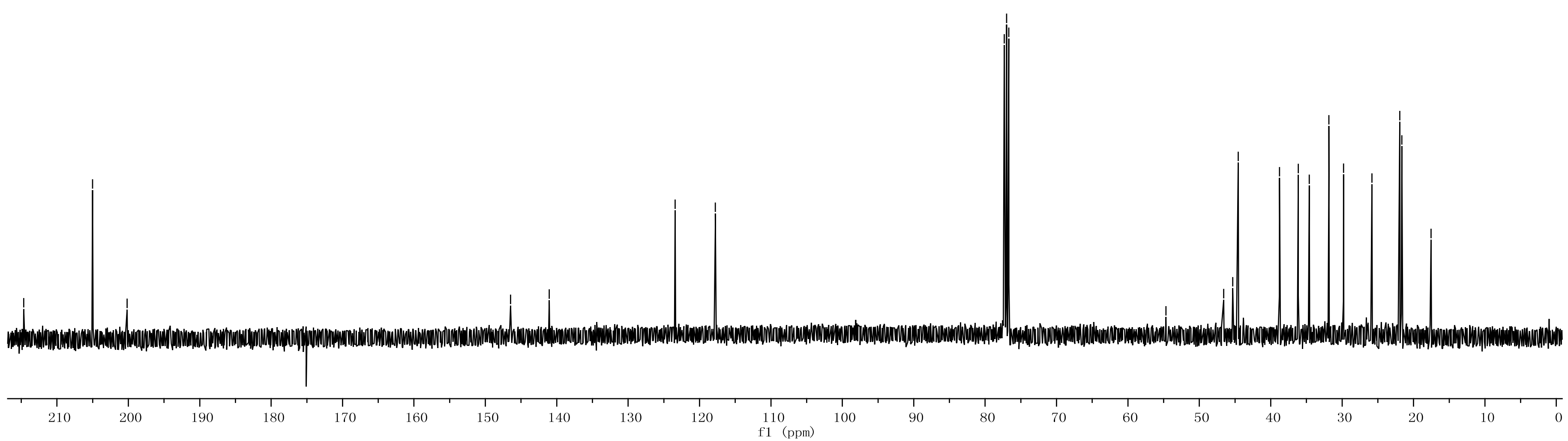

Supplementary Figure 52. ^{13}C NMR spectrum of 33

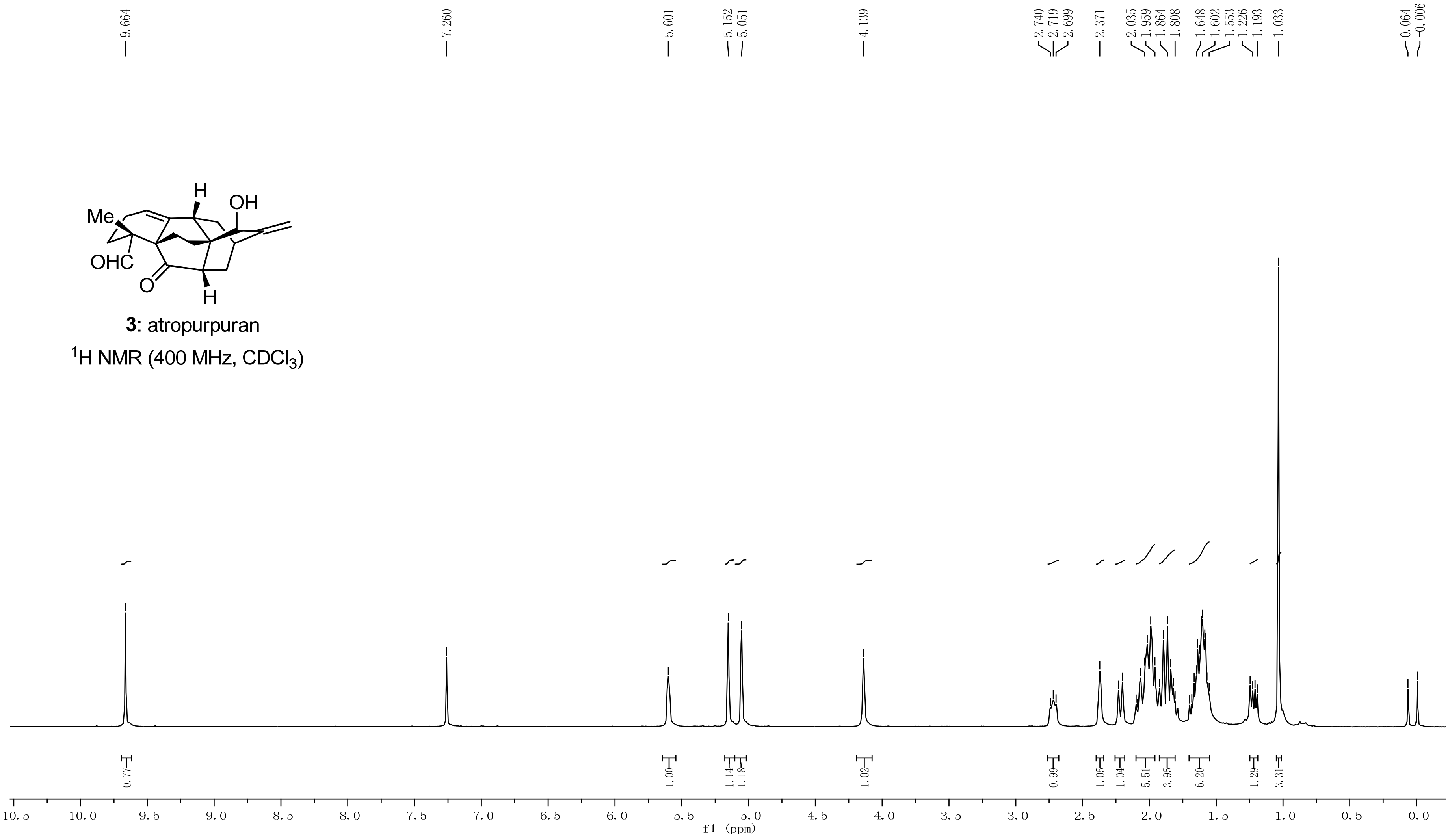

Supplementary Figure 53. ^{1}H NMR spectrum of synthetic atropurpuran 3

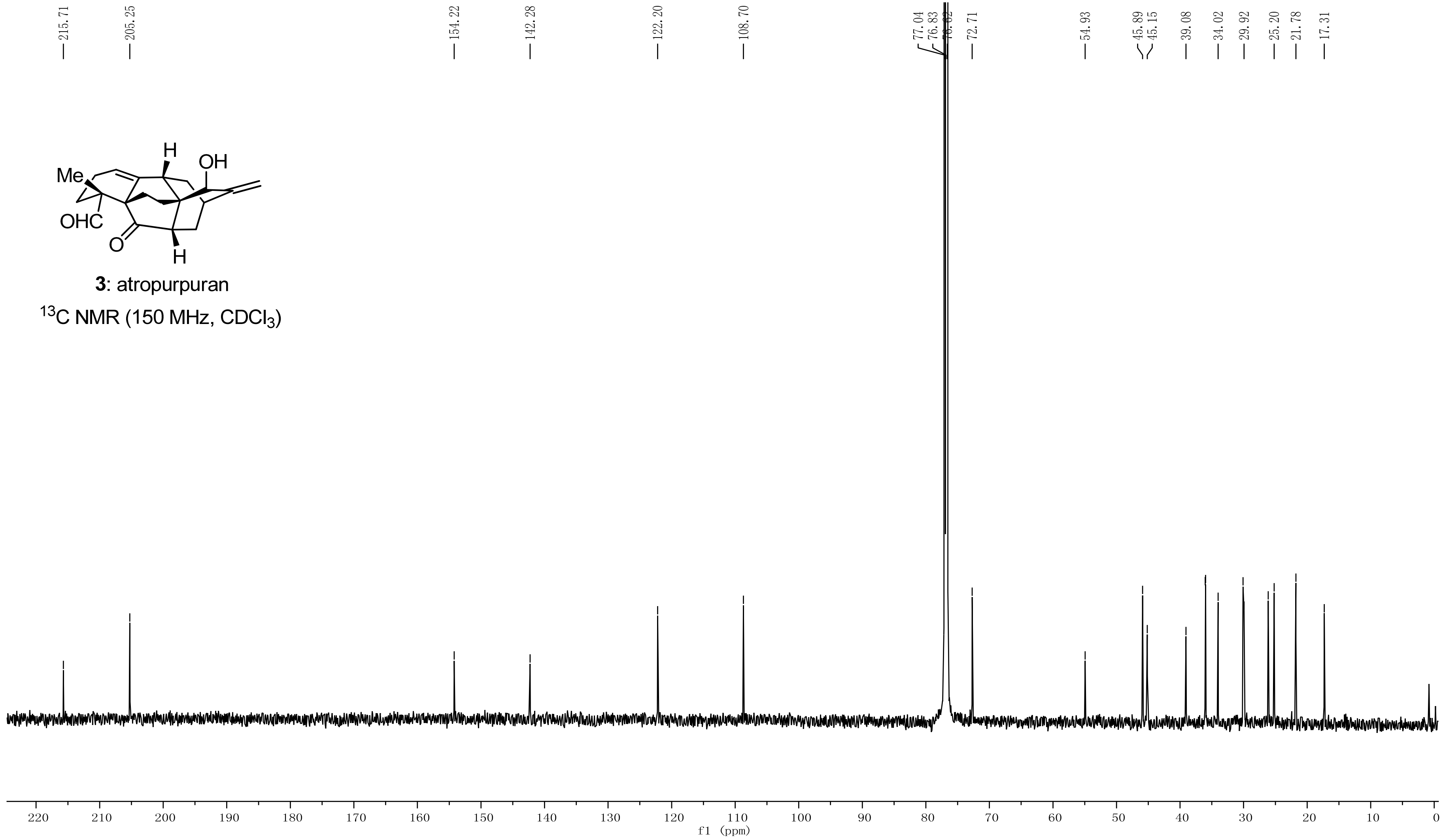

Supplementary Figure 54. ^{13}C NMR spectrum of synthetic atropurpuran 3

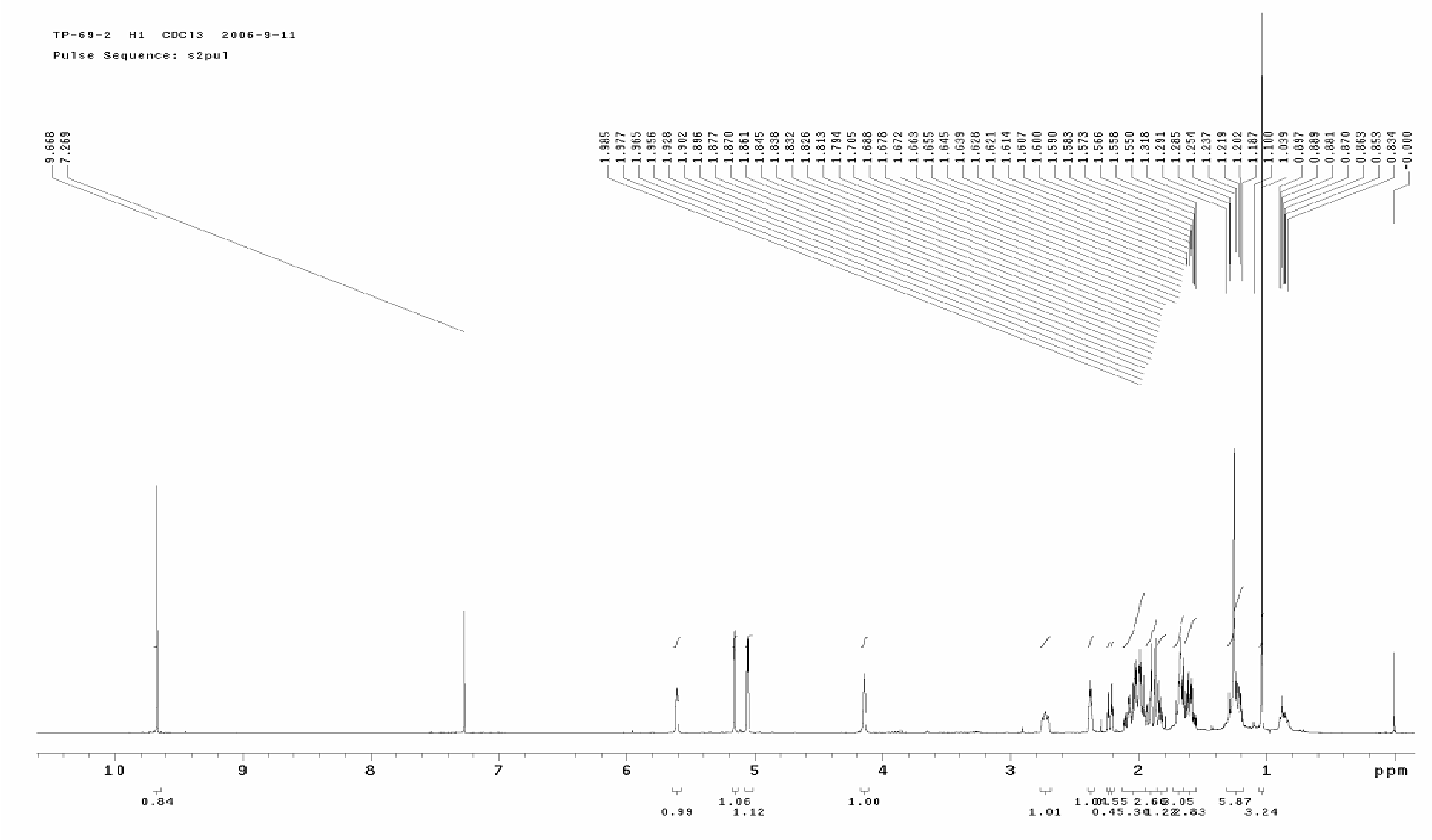

Supplementary Figure 55. ^{1}H NMR spectrum of natural atropurpuran 3

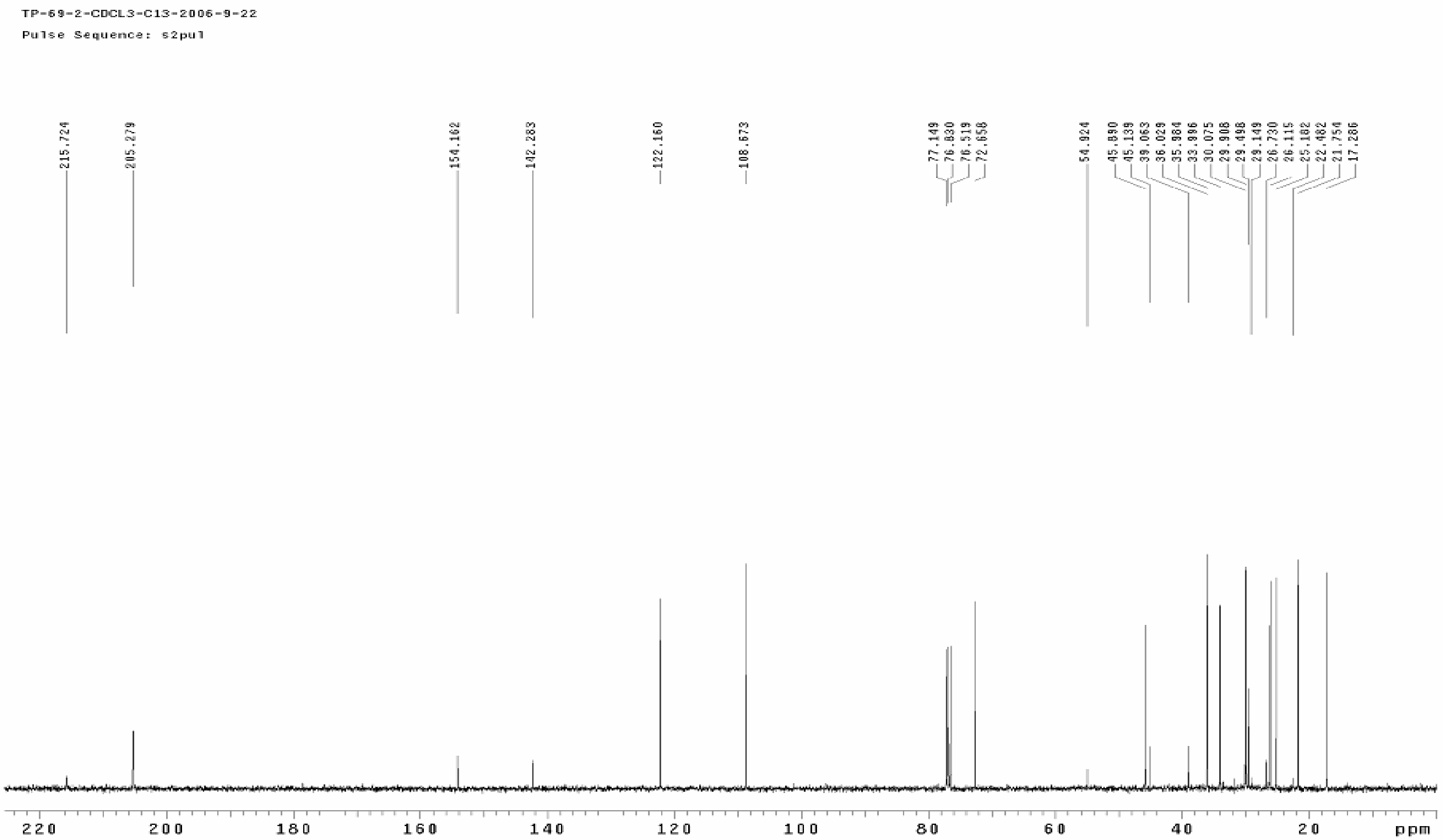

Supplementary Figure 56. ^{13}C NMR spectrum of natural atropurpuran 3

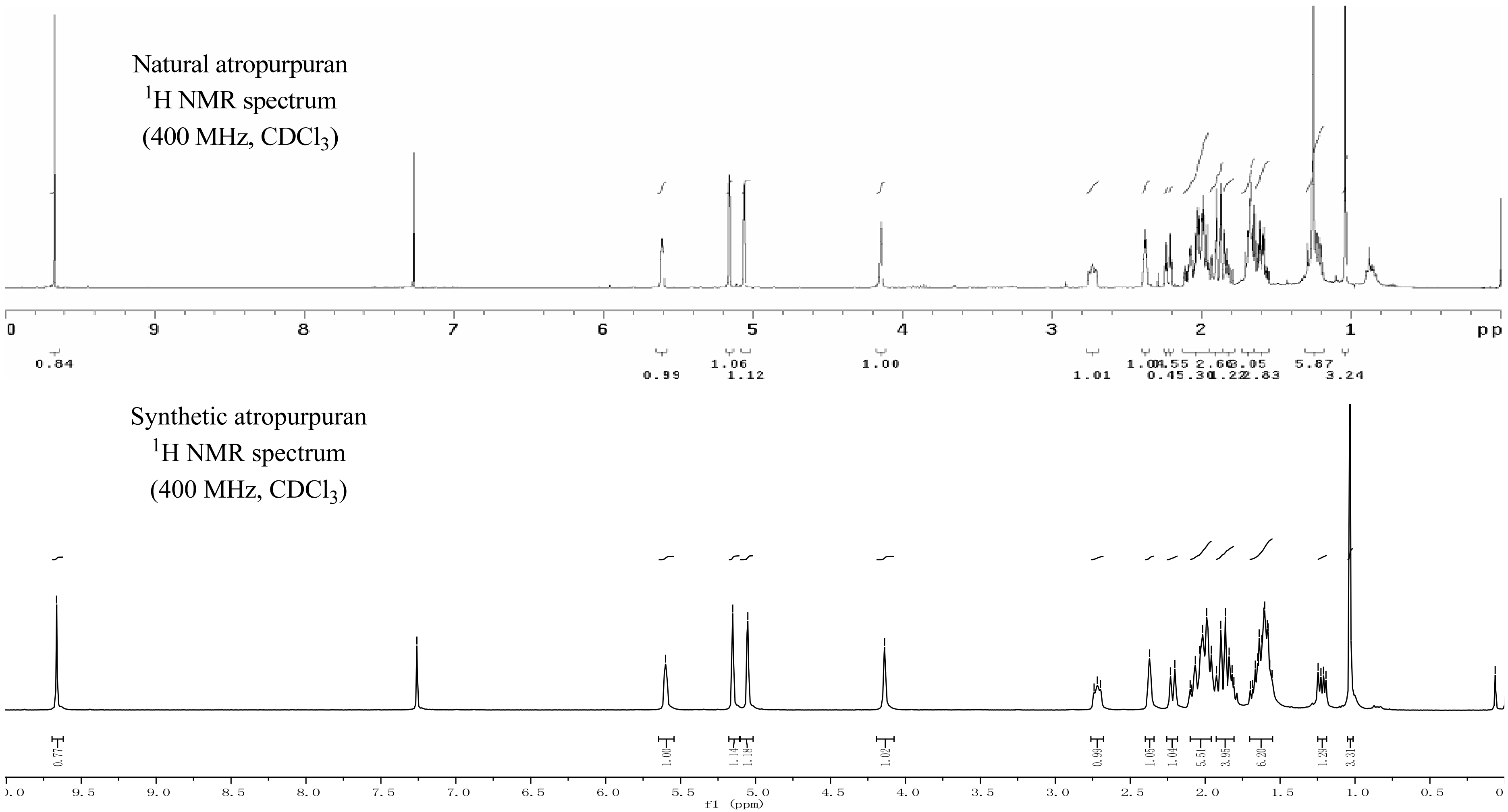

Supplementary Figure 57. Comparison of ^{1}H NMR spectra of the natural and synthetic atropurpuran

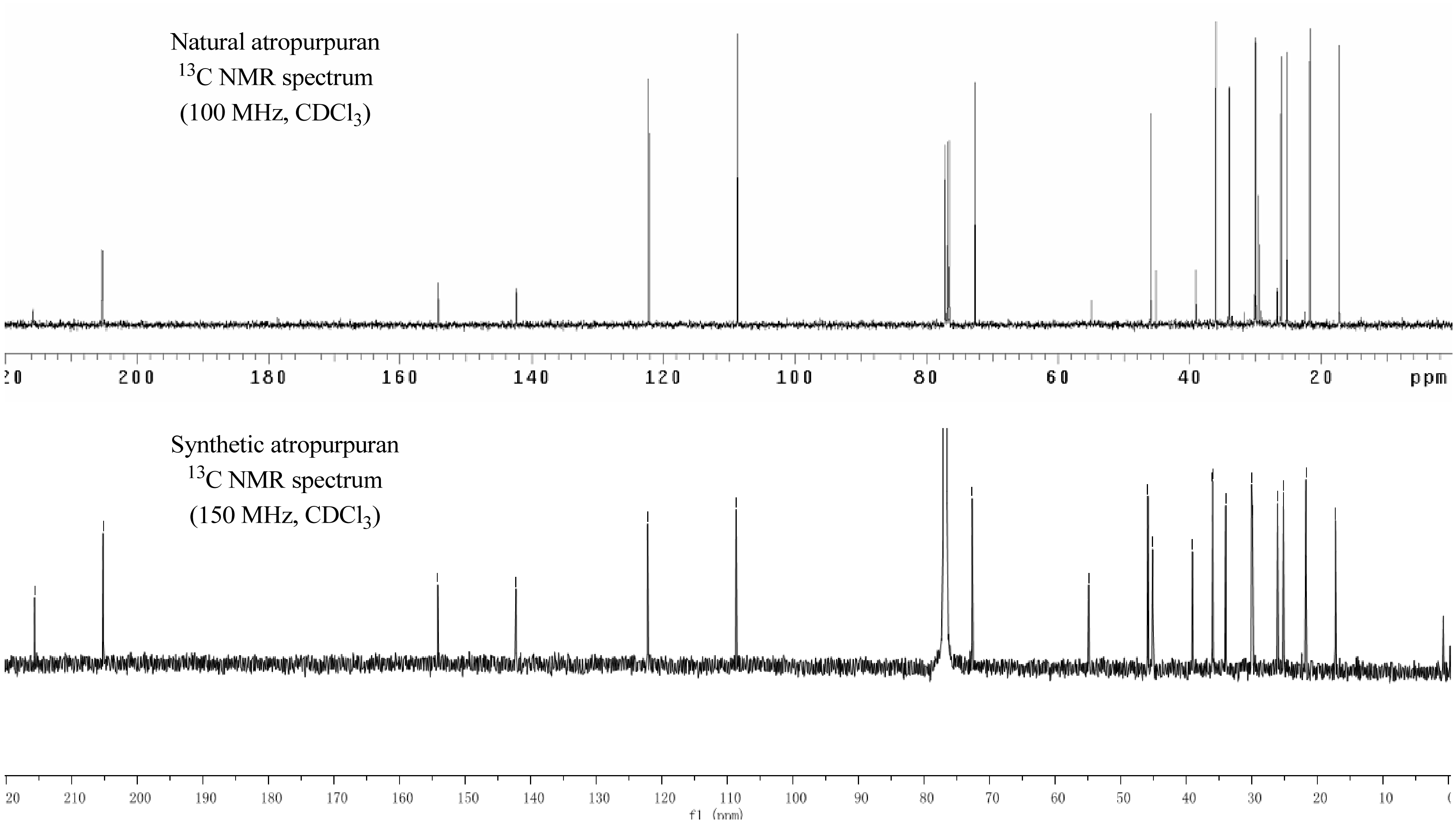

Supplementary Figure 58. Comparison of ^{13}C NMR spectra of the natural and synthetic atropurpuran

Supplementary Tables

Supplementary Table 1. Comparison of ^{1}H NMR ($CDCl_3$) spectroscopic data of the natural and synthetic atropurpuran.

Natural δ_H [ppm, mult, *J* (Hz)] 400 MHz	Synthetic δ_H [ppm, mult, *J* (Hz)] 400 MHz	Err (natural–synthetic) Δδ/ppm
9.67, 1H, s	9.66, 1H, s	0.01
5.61, 1H, t, 4.0	5.60, 1H, s	0.01
5.16, 1H, dd, 6.4, 2.8	5.15, 1H, s	0.01
5.05, 1H, d, 2.4	5.05, 1H, s	0
4.15, 1H, br s	4.14, 1H, s	0.01
2.73, 1H, m	2.74 – 2.70, 1H, m	---
2.37, 1H, m	2.37, 1H, s	0
2.22, 1H, dt, 11.6, 2.4	2.21, 1H, d, 11.6	0.01
2.11, 1H, m	2.10 – 1.96, 5H, m	---
2.08, 1H, m		---
2.04, 1H, m		---
1.98, 1H, m		---
1.95, 1H, m		---
1.90, 1H, m	1.92 – 1.81, 2H, m	---
1.87, 1H, m		---
1.69, 1H, m	1.70 – 1.55, 4H, m	---
1.65, 1H, m		---
1.61, 1H, m		---
1.57, 1H, m		---
1.21, 1H, m	1.23 – 1.19, 1H, m	---
1.04, 3H, s	1.04, 3H, s	0

Supplementary Table 2. Comparison of ^{13}C NMR ($CDCl_3$) spectroscopic data of the natural and synthetic atropurpuran.

Natural δ_C (ppm) 100 MHz	Synthetic δ_C (ppm) 150 MHz	Err (natural–synthetic) Δδ/ppm
215.6	215.7	– 0.1
205.3	205.3	0
154.2	154.2	0
142.3	142.3	0
122.2	122.3	– 0.1
108.7	108.7	0
72.7	72.7	0

54.9	54.9	0
45.9	45.9	0
45.1	45.2	– 0.1
39.1	39.1	0
36.1	36.0	0.1
35.9	36.0	– 0.1
34.0	34.0	0
30.1	30.1	0
30.0	29.9	0.1
26.1	26.1	0
25.2	25.2	0
21.8	21.8	0
17.3	17.3	0

Supplementary Methods

General Experimental

All reactions that require anhydrous conditions were performed in flame-dried glassware under Ar atmosphere and all reagents were purchased from commercial suppliers. Solvent purification was conducted according to Purification of Laboratory Chemicals (Peerrin, D. D.; Armarego, W. L. and Perrins,D. R., Pergamon Press: Oxford, 1980). The products were purified by flash column chromatography on silica gel (200 – 300 meshes) from the Anhui Liangchen Silicon Material Company (China).

Reactions were monitored by thin layer chromatography (TLC) supplied by Yantai Chemicals (China). Visualization was accomplished with UV light, exposure to iodine, stained with ethanolic solution of phosphomolybdic acid or basic solution of $KMnO_4$. ^1H NMR and ^{13}C NMR spectra were recorded on Varian INOVA-400/54 and Agilent DD2-600/54 instruments and calibrated by using residual undeuterated chloroform (δ, ^1H NMR = 7.260, ^{13}C NMR = 77.00). The following abbreviations were used to explain the multiplicities: s = singlet, d = doublet, t = triplet, q = quartet, br = broad, td = triple doublet, dt = double triplet, m = multiplet, and coupling constants (*J*) are reported in Hertz (Hz). Infrared (IR) spectra were recorded on a Perkin Elmer Spectrum Two FT-IR spectrometer. High-resolution mass spectra (HRMS) were recorded on Bruker Apex IV FTMS or Thermo Scientific LTQ Orbitrap XL ESI mass spectrometers.

Experimental Procedures and Characterization Data

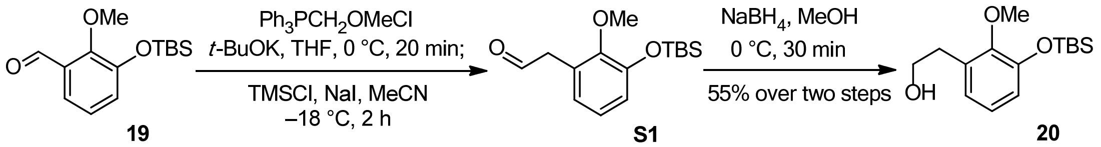

To a suspension of Ph_3PCH_2OMeCl (121.08 g, 353.0 mmol, 4.0 eq.) in THF (400 mL) was added *t*-BuOK (39.66 g, 353.0 mmol, 4.0 eq.) under argon atmosphere at 0 °C. The resulting mixture was stirred for 30 min before a solution of aldehyde **19** (23.57 g, 88.4 mmol, 1.0 eq.) in THF (20 mL) was added. After 20 min, the reaction was quenched with saturated NH_4Cl solution (100 mL), extracted with Et_2O (3 × 100 mL); and the organic layers were washed by water and brine, dried over anhydrous $MgSO_4$, filtered and concentrated in vacuo to give the crude product. The residue was subjected to the conditions of NaI (39.88 g, 265 mmol, 3.0 eq.) and TMSCl (22.9 mL, 265 mmol, 3.0 eq.) in dry MeCN (400 mL) under argon atmosphere at –18 °C for 2 h. Saturated Na_2SO_3 solution (100 mL) was added to quench the reaction. The mixture was moved to room temperature and extracted with Et_2O (3 × 100 mL). The combined organic phase was dried over anhydrous $MgSO_4$, filtered through a silica gel pad, and evaporated to afford crude aldehyde **S1**. Immediately, without further purification, to a solution of the resultant residue in MeOH (400 mL) was added $NaBH_4$ (10.16 g, 265 mmol, 3.0 eq.) by portions under ice-water bath. The reaction was stirred at the same temperature for 30 min and quenched with saturated NH_4Cl solution (100 mL). After removal of MeOH under reduced pressure, the resultant mixture was diluted with EtOAc (200 mL) and water (100 mL), poured into a separatory funnel, partitioned, and the aqueous layer was extracted with EtOAc (3 × 100 mL). The combined organic phase was washed by water and brine, dried and concentrated. Purification of the residue via silica gel chromatography (petroleum ether / ethyl acetate = 4:1) furnished alcohol **20** (13.75 g, 55% over two

steps) as colorless oil.

Compound S1: TLC (petroleum ether / ethyl acetate, 5:1 v/v): $R_f = 0.45$; ^{1}H NMR (400 MHz, $CDCl_3$): δ 9.71 (s, 1H), 6.95 (t, *J* = 7.6 Hz, 1H), 6.83 (d, *J* = 8.0 Hz, 1H), 6.77 (d, *J* = 7.6 Hz, 1H), 3.76 (s, 3H), 3.66 (s, 2H), 1.02 (s, 10H), 0.20 (s, 6H); ^{13}C NMR (100 MHz, $CDCl_3$): δ 199.8, 149.8, 149.0, 126.8, 124.2, 123.7, 120.9, 60.1, 45.4, 25.7, 18.2, –4.6; IR (neat): ν_{max} = 2959, 2931, 2858, 1727, 1587, 1473, 1286, 1254, 1007, 839, 783 cm^{-1}; HRMS (*m/z*): $[M+Na]^+$ calcd. for $C_{15}H_{24}O_3SiNa$, 303.1392; found, 303.1382.

Compound 20: TLC (petroleum ether / ethyl acetate, 4:1 v/v): $R_f = 0.31$; ^{1}H NMR (400 MHz, $CDCl_3$): δ 6.91 (t, *J* = 7.6 Hz, 1H), 6.79 (d, *J* = 7.2 Hz, 1H), 6.76 (d, *J* = 8.0 Hz, 1H), 3.83 (t, *J* = 6.4 Hz, 2H), 3.80 (s, 3H) 2.89 (t, *J* = 6.0 Hz, 2H), 1.98 (s, 1H), 1.01 (s, 9H), 0.19 (s, 6H); ^{13}C NMR (100 MHz, $CDCl_3$): δ 149.6, 148.8, 132.9, 124.0, 123.2, 119.7, 63.4, 60.2, 33.8, 25.7, 18.2, –4.6; IR (neat): ν_{max} = 3344, 2955, 2930, 2858, 1585, 1471, 1285, 1252, 1046, 993, 837, 781 cm^{-1}; HRMS (*m/z*): $[M+Na]^+$ calcd. for $C_{15}H_{26}O_3SiNa$, 305.1549; found, 305.1539.

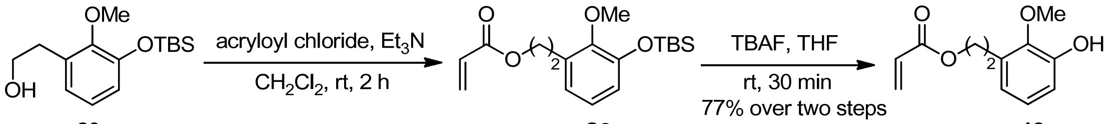

A round bottom flask was charged with alcohol **20** (15.55 g, 55.0 mmol, 1.0 eq.) in dry CH_2Cl_2 (300 mL), followed by sequential dropwise addition of Et_3N (38.1 mL, 275 mol, 5.0 eq.) and acryloyl chloride (18.1 mL, 220 mmol, 4.0 eq.) under argon atmosphere at room temperature. The mixture was stirred for 2 h and then quenched with saturated $NaHCO_3$ solution (150 mL) and extracted with CH_2Cl_2 (3 × 100 mL). The combined organic layers were washed with brine, dried, filtered via a pad of silica gel, and concentrated under reduced vacuum. To a solution of the crude **S2** in THF (300 mL) was added TBAF·3H_2O (52.03 g, 165 mmol, 3.0 eq.). The reaction was stirred for 30 min at room temperature before being quenched with saturated NH_4Cl solution (150 mL) and extracted with EtOAc (3 × 100 mL). The combined organic extracts were washed with water and brine, dried over anhydrous $MgSO_4$ and evaporated to dryness. Purification by flash column chromatography (petroleum ether / ethyl acetate = 5:1) gave phenol **18** (9.412 g, 77% over two steps) as a white foam.

Compound S2: TLC (petroleum ether / ethyl acetate, 5:1 v/v): $R_f = 0.73$; ^{1}H NMR (400 MHz, $CDCl_3$): δ 6.89 (t, *J* = 7.6 Hz, 1H), 6.80 (d, *J* = 6.8 Hz, 1H), 6.76 (d, *J* = 8.0 Hz, 1H), 6.39 (d, *J* = 17.2 Hz, 1H), 6.11 (dd, *J* = 17.2, 10.4 Hz, 1H), 5.81 (d, *J* = 10.4 Hz, 1H), 4.35 (t, *J* = 7.2 Hz, 2H), 3.80 (s, 3H), 2.99 (t, *J* = 7.2 Hz, 2H), 1.01 (s, 9H), 0.19 (s, 6H); ^{13}C NMR (100 MHz, $CDCl_3$): δ 166.0, 149.8, 148.7, 131.7, 130.5, 128.5, 123.7, 123.1, 119.9, 64.5, 60.2, 29.4, 25.7, 18.2, –4.6; IR (neat): ν_{max} = 2956, 2931, 2858, 1726, 2586, 1472, 1407, 1282, 1186, 999, 839, 782 cm^{-1}; HRMS (*m/z*): $[M+Na]^+$ calcd. for $C_{17}H_{26}O_4Na$, 359.1655; found, 359.1643.

Compound 18: TLC (petroleum ether / ethyl acetate, 4:1 v/v): $R_f = 0.32$; ^{1}H NMR (400 MHz, $CDCl_3$): δ 6.96 (t, *J* = 7.6 Hz, 1H), 6.86 (d, *J* = 7.2 Hz, 1H), 6.76 (d, *J* = 7.6 Hz, 1H), 6.40 (d, *J* = 17.2 Hz, 1H), 6.11 (dd, *J* = 17.2, 10.4 Hz, 1H), 5.82 (d, *J* = 10.4 Hz, 1H), 5.61 (s, 1H), 4.39 (t, *J* = 7.2 Hz, 2H), 3.82 (s, 3H), 3.02 (t, *J* = 7.2 Hz, 2H); ^{13}C NMR (100 MHz, $CDCl_3$): δ 166.2, 149.1, 145.7, 130.9, 130.7, 128.3, 124.8, 121.7, 114.5, 64.2, 61.2, 28.9; IR (neat): ν_{max} = 3406, 2953, 1712, 1590, 1473, 1408, 1290, 1190, 1060, 986, 811, 751 cm^{-1}; HRMS (*m/z*): $[M+Na]^+$ calcd. for $C_{12}H_{14}O_4Na$, 245.0790; found,

245.0781.

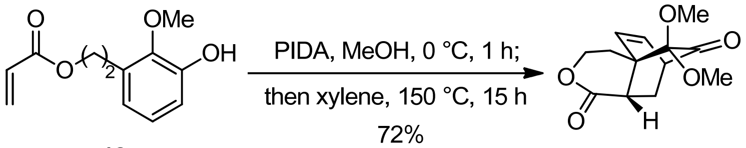

18 **22**

To a solution of phenol **18** (9.405 g, 42.3 mmol, 1.0 eq.) in MeOH (300 mL) was added iodobenzene diacetate (20.316 g, 63.5 mmol, 1.50 eq.) at 0 °C, and the resulting mixture was stirred at the same temperature for 1 h. After removal of methanol, a mixture of the crude residue and BHT (74.62 g, 339 mmol, 8.0 eq.) in xylene (600 mL) was heated at 150 ºC for 15 h. Upon cooling to room temperature, the mixture was concentrated under reduced pressure, and the obtained residue was subjected to flash column chromatography on silica gel (petroleum ether / ethyl acetate = 3:1) to afford Diels-Alder reaction product **22** (7.646 g, 72% overall) as a white solid.

Compound 22: mp: 135 – 136 °C; TLC (petroleum ether / ethyl acetate, 1:1 v/v): $R_f = 0.57$; ^{1}H NMR (400 MHz, $CDCl_3$): δ 6.28 (t, J = 7.2 Hz, 1H), 6.02 (d, J = 8.0 Hz, 1H), 4.47 – 4.41 (m, 1H), 4.39 – 4.32 (m, 1H) 3.46 (s, 3H), 3.32 – 3.29 (m, 4H), 3.24 (t, J = 3.2 Hz, 1H), 2.56 (dt, J = 14.4, 4.4 Hz, 1H), 2.36 (dt, J = 13.6, 4.0 Hz, 1H), 2.15 – 2.04 (m, 2H); ^{13}C NMR (100 MHz, $CDCl_3$): δ 201.3, 174.0, 138.2, 129.1, 94.9, 65.7, 55.5, 52.1, 48.2, 47.9, 38.0, 27.1, 24.1; IR (neat): ν_{max} = 2940, 2839, 1725, 1454, 1395, 1156, 1097, 924, 776, 695 cm^{-1}; HRMS (*m/z*): $[M+Na]^+$ calcd. for $C_{13}H_{16}O_5Na$, 275.0895; found, 275.0886.

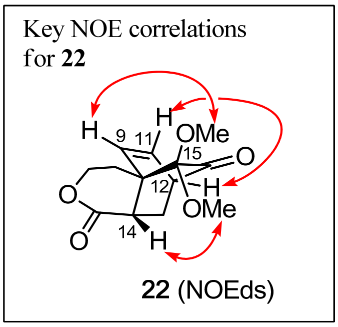

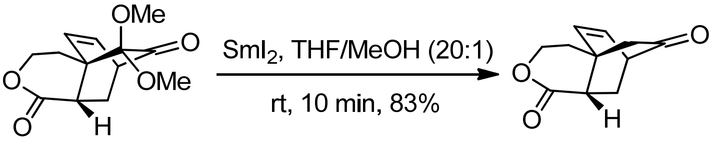

22 **S3**

To a solution of **22** (5.005 g, 19.8 mmol, 1.0 eq.) in THF/MeOH (20:1, 210 mL) was added SmI_2 (0.1 M in THF, 1.59 L, 159 mmol, 8.0 eq.) slowly via cannula at room temperature. The resulting mixture was stirred for 10 min before it was quenched with saturated sodium potassium tartrate solution (600 mL) and kept stirring for 2 h. The organic layer was separated and the aqueous layer was extracted with EtOAc (3 × 300 mL). The combined organic extracts were washed with brine, dried over anhydrous $MgSO_4$ and concentrated in vacuo. Flash column chromatography (petroleum ether / ethyl acetate = 1:1) of the crude product provided ketone **S3** (3.164 g, 83%) as a white solid.

Compound S3: mp: 83 – 84 °C; TLC (petroleum ether / ethyl acetate, 1:1 v/v): $R_f = 0.20$; ^{1}H NMR (400 MHz, $CDCl_3$): δ 6.41 (t, J = 7.2 Hz, 1H), 6.17 (d, J = 8.0 Hz, 1H), 4.51 – 4.48 (m, 2H), 3.25 – 3.23 (t, J = 3.2, 1H), 2.70 (dd, J = 9.6, 5.6 Hz, 1H), 2.31 – 2.24 (m, 2H), 2.20 – 2.07 (m, 4H); ^{13}C NMR (100 MHz, $CDCl_3$): δ 209.6, 172.6, 136.8, 131.2, 65.3, 48.2, 45.6, 42.3, 39.1, 31.3, 25.6; IR (neat): ν_{max} = 2955, 1718, 1481, 1264, 1121, 1050, 739, 687 cm^{-1}; HRMS (*m/z*): $[M+Na]^+$ calcd. for $C_{11}H_{12}O_2Na$, 215.0684; found, 215.0676.

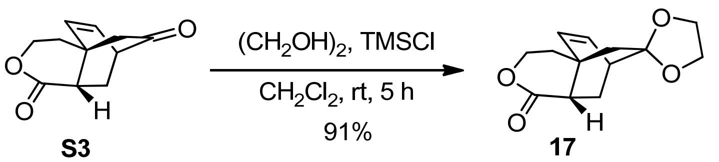

To a solution of ketone **S3** (4.404 g, 22.9 mmol, 1.0 eq.) in dry CH_2Cl_2 (200 mL) were injected ethylene glycol (12.9 mL, 229 mmol, 10.0 eq.) and TMSCl (11.9 mL, 138 mmol, 6.0 eq.) at room temperature. The mixture was stirred for 5 h prior to being quenched with solid Na_2CO_3 (7.706 g) and then saturated $NaHCO_3$ solution (100 mL). The aqueous layer was extracted with CH_2Cl_2 (3 × 50 mL), and the combined organic layers were washed with brine, dried and concentrated. Flash column chromatography of the residue on silica gel (petroleum ether / ethyl acetate = 2:1) was carried out to give lactone **17** (4.923 g, 91%) as a white solid.

Compound 17: mp: 91 – 92 °C; TLC (petroleum ether / ethyl acetate, 1:1 v/v): R_f = 0.32; ^{1}H NMR (400 MHz, $CDCl_3$): δ 6.41 (t, *J* = 7.2 Hz, 1H), 5.99 (d, *J* = 8.0 Hz, 1H), 4.44 – 4.41 (m, 2H), 3.92 (s, 4H), 2.68 (t, *J* = 3.2, 1H), 2.56 (dd, *J* = 9.6, 5.2 Hz, 1H), 2.27 – 2.20 (m, 1H), 2.14 – 2.08 (m, 1H), 2.01 – 1.94 (m, 1H), 1.84 (ddd, *J* = 13.2, 5.2, 2.8 Hz, 1H), 1.76 (dd, *J* = 19.6, 13.2 Hz, 2H); ^{13}C NMR (100 MHz, $CDCl_3$): δ 173.9, 135.1, 134.5, 112.1, 65.6, 64.3, 64.2, 48.4, 42.1, 39.1, 37.9, 32.4, 24.6; IR (neat): ν_{max} = 2958, 2924, 2886, 1736, 1479, 1343, 1143, 1107, 1068, 1016, 721 cm^{-1}; HRMS (*m/z*): $[M+Na]^+$ calcd. for $C_{13}H_{16}O_4Na$, 259.0946; found, 259.0938.

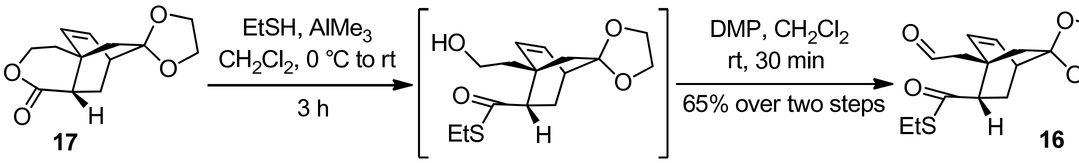

In a flame-dried round-bottomed flask under argon atmosphere, trimethylaluminum (2.2 M in toluene, 23.5 mL, 51.9 mmol, 2.5 eq.) was added dropwise to a solution of ethanethiol (3.83 mL, 51.9 mmol, 2.5 eq.) in CH_2Cl_2 (200 mL) under ice-water bath. The mixture was stirred at the same temperature for 20 min, before a solution of lactone **17** (4.904 g, 20.8 mL, 1.0 eq.)/CH_2Cl_2 (20 mL) was added via cannula. The reaction was kept stirring at room temperature for 3 h, cooled to 0 °C, and quenched by slow addition of saturated sodium potassium tartrate solution (80 mL). After stirring for additional 30 min, the separated aqueous layer was extracted with CH_2Cl_2 (3 × 50 mL), and the combined organic layers were washed by water and brine, and dried over anhydrous $MgSO_4$. After filtration through a silica gel pad and evaporation of the solvent, the crude alcohol was dissolved in CH_2Cl_2 (200 mL). To this solution was added Dess-Martin periodinane (13.2 g, 31.1 mmol, 1.5 eq.) at room temperature. The resulting mixture was stirred at the same temperature for 30 min before it was quenched by saturated $NaHCO_3$ solution (100 mL) and saturated $Na_2S_2O_3$ solution (100 mL), and extracted with CH_2Cl_2 (3 × 80 mL). The combined organic phase was washed with brine, dried and evaporated. Subjection of the residue to flash column chromatography on silica gel (petroleum ether / ethyl acetate = 6:1) furnished aldehyde **16** (4.02 g, 65% over two steps) as colorless oil, along with recovered lactone **17** (0.502 g, 10% recycling yield).

Compound 16: TLC (petroleum ether / ethyl acetate, 2:1 v/v): R_f = 0.50; ^{1}H NMR (400 MHz, $CDCl_3$): δ 9.83 (s, 1H), 6.39 (t, *J* = 7.6 Hz, 1H), 6.17 (d, *J* = 8.0 Hz, 1H), 3.90 (s, 4H), 3.07 (dd, *J* = 9.6, 6.0 Hz, 1H), 2.82 (dd, *J* = 14.8, 7.6 Hz, 2H), 2.76 – 2.63 (m, 4H), 2.39 – 2.33 (m, 1H), 1.85 (d, *J* = 13.6 Hz,

1H), 1.70 (d, *J* = 13.6 Hz, 1H), 1.43 (ddd, *J* = 12.8, 6.0, 2.8 Hz, 1H), 1.21 (t, *J* = 7.2 Hz, 3H); ^{13}C NMR (100 MHz, $CDCl_3$): δ 201.5, 200.6, 134.6, 132.1, 111.9, 64.2, 64.1, 54.4, 48.7, 46.7, 40.3, 39.1, 29.5, 23.4, 14.5; IR (neat): ν_{max} = 2963, 2931, 2882, 2731, 1720, 1681, 1450, 1341, 1104, 1017, 979, 872, 707 cm^{-1}; HRMS (*m/z*): $[M+Na]^+$ calcd. for $C_{15}H_{20}O_4SNa$, 319.0980; found, 319.0970.

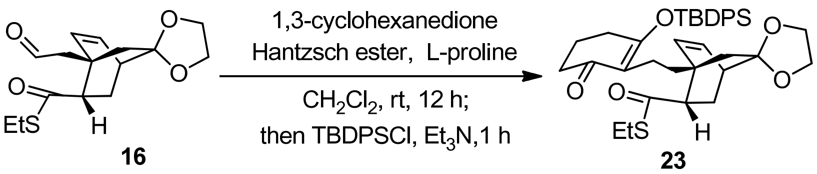

To a stirred solution of **16** (4.012 g, 13.5 mmol, 1.0 eq.) in CH_2Cl_2 (200 mL) was added 1,3-cyclohexanedione (3.044 g, 27.1 mmol, 2.0 eq.), Hantzsch ester (6.87 g, 27.1 mmol, 2.0 eq.) and L-proline (0.31 g, 2.71 mmol, 0.2 eq.) at room temperature. The reaction was stirred at room temperature for 12 h, followed by addition of Et_3N (5.62 mL, 40.6 mmol, 3.0 eq.) and *tert*-butylchlorodiphenylsilane (8.78 mL, 33.9 mmol, 2.5 eq.). The resulting mixture was stirred for an additional hour and quenched with saturated Na_2SO_3 solution (100 mL). After extraction with CH_2Cl_2 (3 × 50 mL) of the separated aqueous layer, the combined organic phase was washed by water and brine, dried over anhydrous $MgSO_4$ and concentrated under vacuum. The crude product was purified through column chromatography (petroleum ether / ethyl acetate = 20:1 to 8:1) to give thioester **23** (7.035 g, 82 % overall) as colorless oil.

Compound 23: TLC (petroleum ether / ethyl acetate, 5:1 v/v): R_f = 0.46; ^{1}H NMR (400 MHz, $CDCl_3$): δ 7.70 (t, *J* = 5.2 Hz, 4H), 7.48 – 7.43 (m, 6H), 6.36 – 6.35 (m, 2H), 3.92 – 3.91 (m, 4H), 2.87 – 2.73 (m, 3H), 2.64 (s, 1H), 2.51 – 2.49 (m, 2H), 2.34 (t, *J* = 10.4 Hz, 1H), 2.21 (t, *J* = 6.4 Hz, 2H), 1.99 (t, *J* = 5.6 Hz, 2H), 1.90 (d, *J* = 13.6 Hz, 1H), 1.77 – 1.70 (m, 1H), 1.65 – 1.58 (m, 3H), 1.44 – 1.40 (m, 2H), 1.19 (t, *J* = 7.2 Hz, 3H), 1.07 (s, 9H); ^{13}C NMR (100 MHz, $CDCl_3$): δ 201.8, 198.8, 169.4, 136.1, 134.8, 132.4, 131.2, 130.3, 128.0, 122.2, 112.6, 64.1, 64.0, 56.1, 45.7, 42.9, 39.1, 36.7, 33.9, 31.5, 30.6, 26.8, 26.3, 23.3, 21.0, 19.2, 17.9, 14.6; IR (neat): ν_{max} = 2956, 2938, 2858, 1691, 1654, 1613, 1428, 1365, 1237, 1102, 996, 702, 502 cm^{-1}; HRMS (*m/z*): $[M+Na]^+$ calcd. for $C_{37}H_{46}O_5SSiNa$, 653.2733; found, 653.2708.

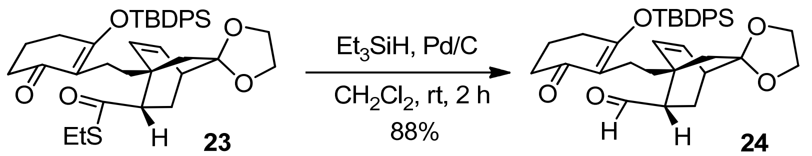

To a stirred solution of the thioester **23** (7.003 g, 11.1 mmol, 1.0 equiv) and Pd/C (10%, 350 mg, 5% mol) in dry CH_2Cl_2 (22 mL) was slowly added Et_3SiH (5.30 mL, 33.3 mmol, 3.0 eq.), and the mixture was stirred at room temperature for 2 h. After filtration, the collected filtrate was concentrated to a residue, which was purified by flash chromatography on silica gel (petroleum ether / ethyl acetate = 5:1). The desired product **24** (5.572 g, 88%) was obtained as colorless oil.

Compound 24: TLC (petroleum ether / ethyl acetate, 5:1 v/v): R_f = 0.35; ^{1}H NMR (400 MHz, $CDCl_3$): δ 9.42 (d, *J* = 4.0 Hz, 1H), 7.71 – 7.68 (m, 4H), 7.50 – 7.44 (m, 6H), 6.40 (t, *J* = 6.8 Hz, 1H), 6.29 (d, *J* = 8.0 Hz, 1H), 3.92 (s, 4H), 2.68 (t, *J* = 3.2 Hz, 1H), 2.59 – 2.52 (m, 3H), 2.22 (t, *J* = 6.8 Hz, 2H), 2.18 – 2.12 (m, 1H), 2.00 (t, *J* = 2.0 Hz, 2H), 1.85 (d, *J* = 13.6 Hz, 1H), 1.75 – 1.67 (m, 3H), 1.64 – 1.56 (m,

2H), 1.42 (s, 1H), 1.06 (s, 9H); ^{13}C NMR (100 MHz, $CDCl_3$): δ 204.5, 199.0, 169.6, 135.3, 134.8, 132.8, 132.3, 130.4, 128.1, 121.9, 112.6, 64.2, 64.0, 53.8, 45.2, 42.1, 38.6, 36.6, 34.5, 31.5, 26.3, 23.8, 20.9, 19.3, 17.8; IR (neat): ν_{max} = 3051, 2931, 2862, 1719, 1652, 1615, 1428, 1367, 1237, 1106, 928, 703, 502 cm^{-1}; HRMS (*m/z*): $[M+Na]^+$ calcd. for $C_{35}H_{42}O_5SiNa$, 593.2699; found, 593.2676.

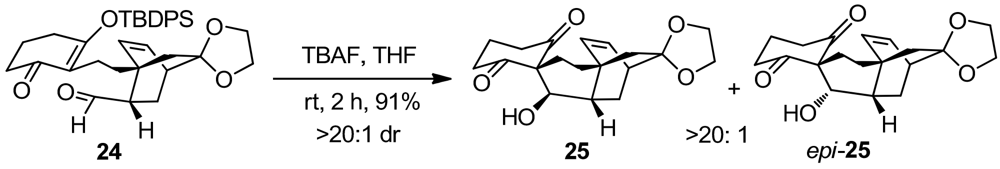

A mixture of aldehyde **24** (5.552 g, 9.74 mmol, 1.0 eq.) and TBAF (1.0 M in THF, 9.74 mL, 9.74 mmol, 1.0 eq.) in THF (200 mL) was stirred at room temperature for 2 h. The reaction was quenched with saturated NH_4Cl solution (100 mL) and extracted with EtOAc (3 × 50 mL). The combined organic phase was washed with water and brine, and dried over anhydrous $MgSO_4$. Evaporation of solvent gave the crude product, which after a flash column chromatography (petroleum ether / ethyl acetate = 2:1) yielded an inseparable mixture of alcohol **25** and *epi*-**25** (2.933 g, 91%, >20: 1 dr) as a white solid.

Compound 25: mp: 110 – 111 °C; TLC (petroleum ether / ethyl acetate, 2:1 v/v): R_f = 0.22; ^{1}H NMR (400 MHz, $CDCl_3$): δ 6.34 (t, J = 7.2 Hz, 1H), 5.95 (d, J = 8.0 Hz, 1H), 3.91 – 3.89 (m, 4H), 3.55 (t, J = 10.0 Hz, 1H), 2.87 – 2.79 (m, 1H), 2.75 – 2.67 (m, 1H), 2.62 – 2.52 (m, 3H), 2.41 (td, J = 10.0, 4.8 Hz, 1H), 2.24 – 2.18 (m, 1H), 2.34 – 2.00 (m, 3H), 1.92 – 1.80 (m, 2H), 1.64 – 1.52 (m, 2H), 1.39 (td, J = 14.0, 3.6 Hz, 1H), 1.15 – 1.11 (m, 1H); ^{13}C NMR (100 MHz, $CDCl_3$): δ 210.6, 209.5, 134.4, 133.0, 112.8, 75.7, 70.2, 64.1, 63.9, 48.5, 41.4, 40.0, 39.0, 38.6, 38.5, 30.6, 29.5, 27.2, 17.2; IR (neat): ν_{max} = 3475, 2946, 2878, 1720, 1687, 1445, 1320, 1094, 1023, 719 cm^{-1}; HRMS (*m/z*): $[M+Na]^+$ calcd. for $C_{19}H_{24}O_5Na$, 355.1508; found, 355.1521.

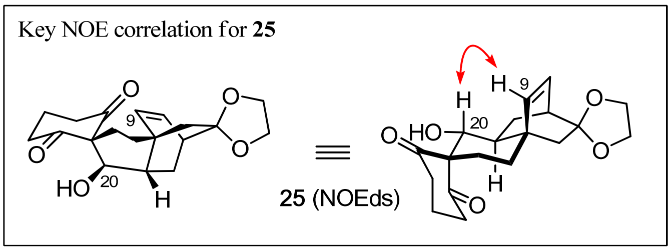

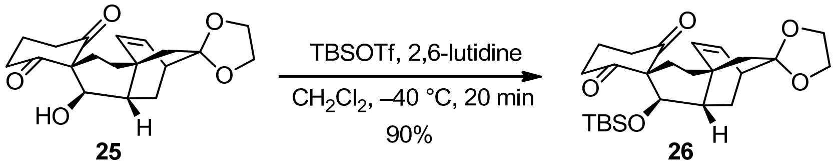

To a solution of alcohol **25** (1.303 g, 3.92 mmol, 1.0 eq.) in dry CH_2Cl_2 (200 mL) was added 2,6-lutidine (16.4 mL, 141 mmol, 36.0 eq.) at –40 °C, and 10 min later, adding TBSOTf (26.7 mL, 117 mmol, 30.0 eq.). The resulting mixture was allowed to keep stirring for another 40 min and quenched with saturated $NaHCO_3$ solution (100 mL) at the same temperature. After being warmed to room temperature, the layers were separated and the upper aqueous phase was extracted with CH_2Cl_2 (3 × 50 mL). The combined organic extracts were washed with brine, dried and evaporated under vacuum. Purification of the residue by flash column chromatography (silica gel, petroleum ether / ethyl acetate = 5:1) gave diketone **26** (1.571 g, 90%) as a white solid.

Compound 26: mp: 114 – 115 °C; TLC (petroleum ether / ethyl acetate, 4:1 v/v): R_f = 0.36; ^{1}H NMR

(400 MHz, $CDCl_3$): δ 6.33 (t, *J* = 7.2 Hz, 1H), 5.96 (d, *J* = 8.0 Hz, 1H), 3.93 – 3.85 (m, 5H), 2.71 – 2.63 (m, 3H), 2.57 – 2.46 (m, 3H), 2.16 – 2.10 (m, 1H), 2.06 – 1.87 (m, 3H), 1.69 – 1.64 (m, 3H), 1.53 – 1.49 (m, 2H), 0.98 – 0.93 (m, 1H), 0.77 (s, 9H), 0.04 (s, 3H), –0.13 (s, 3H); ^{13}C NMR (100 MHz, $CDCl_3$): δ 211.5, 207.9, 135.1, 132.6, 113.0, 76.9, 68.8, 64.1, 63.9, 48.5, 40.8, 40.5, 40.0, 38.9, 38.3, 30.7, 28.6, 26.1, 18.4, 16.8, –3.3, –4.6; IR (neat): ν_{max} = 2952, 2931, 2856, 1723, 1694, 1472, 1252, 1095, 1062, 830, 775, 713 cm^{-1}; HRMS (*m/z*): $[M+Na]^+$ calcd. for $C_{25}H_{38}O_5SiNa$, 469.2386; found, 469.2372.

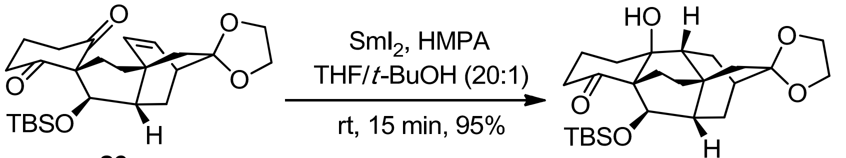

26 **27**

To a degassed mixture of HMPA (10.4 mL, 58.2 mmol, 20.0 eq.) and SmI_2 (0.1 M in THF, 233 mL, 23.3 mmol, 8.0 eq.) was added dropwise a solution of **26** (1.302 g, 2.91 mmol, 1.0 eq.) and *t*-BuOH (3.0 mL) in THF (60 mL), which was also degassed prior to use. The reaction was stirred at room temperature until the color changed from violet to grey (within 15 min). Saturated sodium potassium tartrate solution (100 mL) was then added, and the resulting mixture was extracted with EtOAc (3 × 50 mL). The combined organic layers were washed with brine, dried with anhydrous $MgSO_4$ and filtered. Removal of solvent followed by purification through flash column chromatography on silica gel (petroleum ether / ethyl acetate = 3:1) yielded tertiary alcohol **27** (1.242 g, 95%) as a white solid.

Compound 27: mp : 171 – 172 °C; TLC (petroleum ether / ethyl acetate, 2:1 v/v): R_f = 0.32; ^1H NMR (400 MHz, $CDCl_3$): δ 3.94 – 3.92 (m, 3H), 3.88 – 3.86 (m, 1H) 3.84 – 3.81 (m, 1H), 2.37 – 2.26 (m, 4H), 2.09 – 1.90 (m, 4H), 1.74 – 1.53 (m, 5H), 1.50 – 1.31 (m, 5H), 0.90 – 0.85 (m, 1H), 0.80 (s, 9H), 0.05 (s, 3H), 0.01 (s, 3H); ^{13}C NMR (100 MHz, $CDCl_3$): δ 211.7, 110.2, 80.3, 79.0, 64.0, 63.8, 59.4, 47.1, 45.0, 42.0, 39.4, 34.2, 33.4, 32.4, 31.5, 29.6, 25.5, 24.7, 21.5, 17.8, 15.2, –3.9, –5.2; IR (neat): ν_{max} = 3475, 2949, 2928, 2856, 1704, 1461, 1250, 1083, 835, 773, 734 cm^{-1}; HRMS (*m/z*): $[M+Na]^+$ calcd. for $C_{25}H_{40}O_5SiNa$, 471.2543; found, 471.2526.

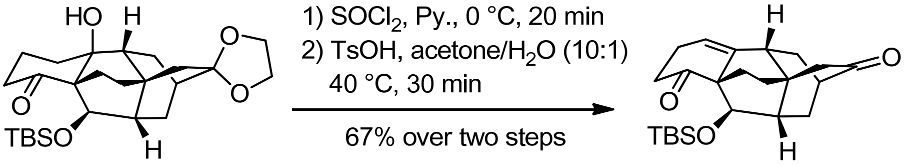

27 **28**

To a solution of tertiary alcohol **27** (1.282 g, 2.86 mmol, 1.0 eq.) in pyridine (100 mL) was slowly injected $SOCl_2$ (3.07 mL, 42.9 mmol, 15.0 eq.) at 0 °C, and the reaction was stirred for 20 min at the same temperature before being quenched with saturated NH_4Cl solution (100 mL). The separated aqueous phase was extracted with EtOAc (3 × 50 mL). After the combined organic extracts were washed with brine, dried over anhydrous $MgSO_4$, and filtered. The solvent was removed under reduced pressure. Without further purification, the resulting residue was dissolved in acetone / H_2O (10:1, 110 mL) and treated with $TsOH{\cdot}H_2O$ (816 mg, 4.29 mmol, 1.5 eq.). The reaction was completed after stirring at 40 °C for 30 min and subjected to evaporation under vacuum. Saturated $NaHCO_3$ solution (50 mL) was then poured into the residue, and the mixture was extracted with EtOAc (3 × 50 mL). The combined organic layers were washed with brine, dried and filtered. After removal of the solvent, the

crude product was subjected to flash column chromatography on silica gel (petroleum ether / ethyl acetate = 10:1) to afford ketone **28** (742 mg, 67%) as colorless oil.

Compound 28: TLC (petroleum ether / ethyl acetate, 5:1 v/v): $R_f = 0.72$; ^{1}H NMR (400 MHz, $CDCl_3$): δ 5.70 (d, J = 5.2 Hz, 1H), 3.75 (s, 1H), 2.78 – 2.70 (m, 1H), 2.58 – 2.51 (m, 2H), 2.45 – 2.40 (m, 1H), 2.36 – 2.25 (m, 5H), 2.17 (d, J = 18.8 Hz, 1H), 1.99 (d, J = 18.8 Hz, 1H), 1.92 (dd, J = 12.8, 3.6 Hz, 1H), 1.61 – 1.55 (m, 2H), 1.51 – 1.47 (m, 1H), 1.44 – 1.37 (m, 1H), 1.19 – 1.11 (m, 1H), 0.85 (s, 9H), –0.03 (s, 3H), –0.07; ^{13}C NMR (100 MHz, $CDCl_3$): δ = 216.0, 210.1, 146.8, 121.8, 80.6, 53.7, 46.9, 45.4, 43.3, 36.1, 35.8, 35.5, 33.5, 31.8, 28.4, 26.8, 25.6, 18.6, 17.8, –4.7, –5.2; IR (neat): ν_{max} = 2929, 2856, 1771, 1461, 1359, 1248, 1081, 1006, 858, 836, 777 cm^{-1}; HRMS (m/z): $[M+H]^+$ calcd. for $C_{23}H_{35}O_3Si$, 387.2355; found, 387.2350.

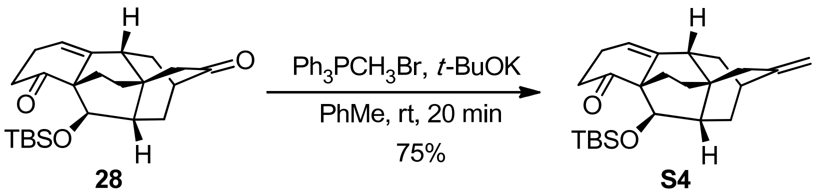

To a suspension of Ph_3PCH_3Br (3.221 g, 9.02 mmol, 4.0 eq.) in dry toluene (100 mL) was added *t*-BuOK (1.0 M in *t*-BuOH, 8.56 mL, 8.56 mmol, 3.8 eq.) at 0 °C. Then the mixture was stirred at room temperature for 30 min before a solution of **28** (870 mg, 2.25 mmol, 1.0 eq.) in toluene (10 mL) was injected. The resulting reaction was completed in additional 20 min and quenched by saturated NH_4Cl solution (100 mL). After extraction with EtOAc (3 × 50 mL), the combined organic phase was washed with brine, dried over anhydrous $MgSO_4$, filtered and concentrated in vacuo to give the crude product. Flash column chromatography over silica gel (petroleum ether / ethyl acetate = 30:1) afforded compound **S4** (650 mg, 75%) as a white foam.

Compound S4: TLC (petroleum ether / ethyl acetate, 10:1 v/v): $R_f = 0.64$; ^{1}H NMR (400 MHz, $CDCl_3$): δ 5.62 (t, J = 2.4 Hz, 1H), 4.72 (s, 1H), 4.59 (s , 1H), 3.66 (s, 1H), 2.76 – 2.69 (m, 1H), 2.54 – 2.47 (m, 2H), 2.41 – 2.37 (m, 1H), 2.32 – 2.28 (m, 1H), 2.23 (s, 1H), 2.16 – 2.09 (m, 2H), 2.06 – 2.00 (m, 3H), 1.72 (dd, J = 12.4, 3.6 Hz, 1H), 1.52 – 1.44 (m, 1H), 1.42 – 1.31 (m, 2H), 1.28 – 1.25 (m, 1H), 1.15 – 1.07 (m, 1H), 0.85 (s, 9H), –0.03 (s, 3H), –0.08 (s, 3H); ^{13}C NMR (100 MHz, $CDCl_3$): δ 211.1, 151.4, 148.3, 120.4, 104.7, 81.4, 54.0, 45.7, 38.9, 36.9, 36.8, 36.6, 36.2, 35.4, 33.5, 28.9, 26.9, 25.7, 19.3, 17.8, –4.7, –5.2; IR (neat): ν_{max} = 3323, 2927, 2855, 1712, 1652, 1461, 1253, 1077, 836, 774 cm^{-1}; HRMS (m/z): $[M+H]^+$ calcd. for $C_{24}H_{37}O_2Si$, 385.2563; found, 385.2557.

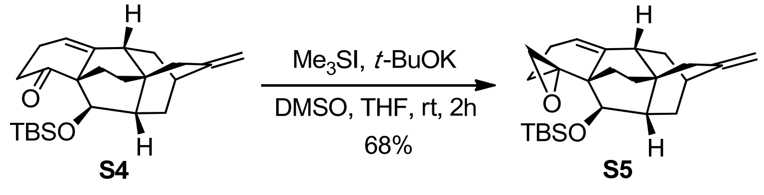

t-BuOK (1.0 M in *t*-BuOH, 10.0 mL, 10.0 mmol, 6.0 eq.) was syringed to a solution of trimethylsulfoxonium iodide (2.72 g, 13.3 mmol, 8.0 eq.) in DMSO (100 mL) under an atmosphere of argon. A solution of **S4** (640 mg, 1.67 mmol, 1.0 eq.) in THF (100 mL) was added in one portion and the mixture was continued to stir for 2 h. The resulting mixture was diluted with EtOAc (80 mL) and water (100 mL). After the aqueous layer was separated and extracted with EtOAc (3 × 30 mL), the

combined organic extracts were washed with brine (three times), dried, filtered and evaporated. Subjection of the crude product to flash column chromatography (silica gel, petroleum ether / ethyl acetate = 60:1) afforded epoxide **S5** (450 mg, 68%) as a white powder.

Compound S5: TLC (petroleum ether / ethyl acetate, 20:1 v/v): R_f = 0.32; ^{1}H NMR (400 MHz, $CDCl_3$): δ 5.63 (s, 1H), 4.69 (d, J = 2.0 Hz, 1H), 4.56 (d, J = 2.0 Hz, 1H), 3.66 (s, 1H), 2.88 (d, J = 5.6 Hz, 1H), 2.27 – 2.22 (m, 2H), 2.17 (s, 1H), 2.13 – 2.09 (m, 3H), 2.00 – 1.86 (m, 4H), 1.73 – 1.61 (m, 2H), 1.54 – 1.46 (m, 2H), 1.32 – 1.14 (m, 4H), 0.89 (s, 9 H), 0.09 (s, 3H), 0.03 (s, 3H); ^{13}C NMR (100 MHz, $CDCl_3$): δ 151.9, 147.9, 121.9, 104.4, 78.4, 61.3, 50.3, 46.1, 42.8, 39.0, 37.1, 36.6, 35.8, 34.4, 32.9, 30.1, 29.5, 26.0, 24.0, 22.6, 17.8, –4.4, –4.9; IR (neat): ν_{max} = 3369, 2927, 2854, 1651, 1460, 1247, 1061, 861, 834, 776 cm^{-1}; HRMS (m/z): $[M+H]^+$ calcd. for $C_{25}H_{39}O_2Si$, 399.2719; found, 399.2714.

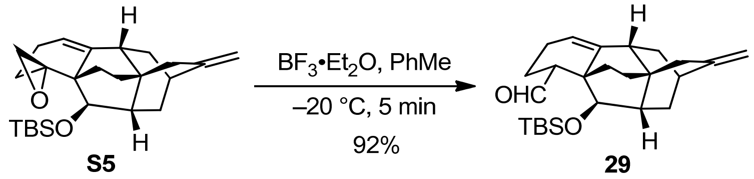

To a solution of epoxide **S5** (403 mg, 1.01 mmol, 1.0 eq.) in dry toluene (50 mL) was added BF_3•OEt_2 (100 μL, 0.810 mmol, 0.8 eq.) under the atmosphere of argon at –20 °C, and the reaction was stirred at the same temperature for 5 min before it was quenched by saturated $NaHCO_3$ solution (20 mL) and extracted with CH_2Cl_2 (3 × 10 mL). Organic layers were combined and washed with brine, and then dried over anhydrous $MgSO_4$, filtered, concentrated. Purification through flash column chromatography on silica gel (petroleum ether / ethyl acetate = 60:1) afforded aldehyde **29** (370 mg, 92%) as a pale yellow gum.

Compound 29: TLC (petroleum ether / ethyl acetate, 10:1 v/v): R_f = 0.48; ^{1}H NMR (400 MHz, $CDCl_3$): δ 9.87 (s, 1H), 5.55 (s, 1H), 4.69 (s, 1H), 4.56 (s, 1H), 3.56 (s, 1H), 2.41 (t, J = 10.8 Hz, 1H), 2.17–2.11 (m, 4H), 2.03 – 1.92 (m, 7H), 1.67 – 1.62 (m, 1H), 1.57 – 1.50 (m, 2H), 1.41 – 1.30 (m, 3H), 1.19 (dd, J = 12.0, 6.8 Hz, 1H), 0.88 (s, 9H), 0.03 (s, 3H), –0.03 (s, 3H); ^{13}C NMR (100 MHz, $CDCl_3$): δ 203.1, 151.6, 146.7, 121.9, 104.5, 78.1, 54.1, 44.7, 44.2, 39.0, 37.2, 36.5, 36.1, 34.9, 33.0, 29.9, 25.9, 25.6, 24.4, 18.5, 17.8, –3.7, –4.9; IR (neat): ν_{max} = 2951, 2927, 2856, 1715, 1651, 1462, 1361, 1253, 1077, 1060, 1050, 1018, 825, 775 cm^{-1}; HRMS (m/z): $[M+H]^+$ calcd. for $C_{25}H_{39}O_2Si$, 399.2719; found, 399.2708.

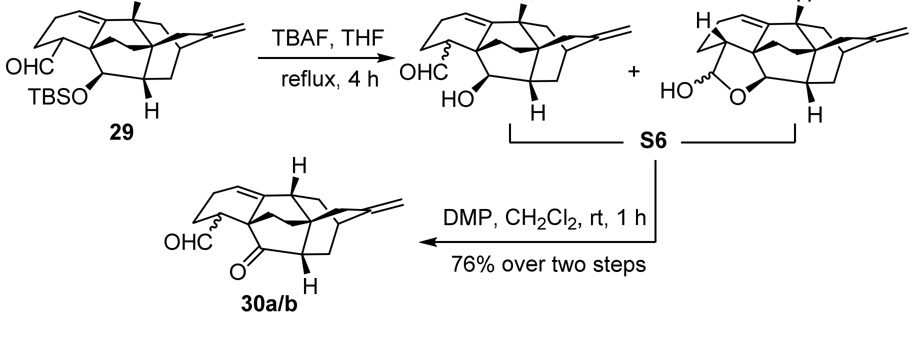

A solution of aldehyde **29** (312 mg, 0.784 mmol, 1.0 eq.) and TBAF (1.0 M in THF, 2.35 mL, 2.35 mmol, 3.0 eq.) in THF (40 mL) was heated under reflux for 4 h. Upon cooling to room temperature, the

mixture was poured into saturated NH_4Cl solution (30 mL) and extracted with EtOAc (3 × 20 mL). The organic extracts were combined, washed with brine, dried over anhydrous $MgSO_4$, filtered through a pad of silica gel, and concentrated to dryness. The resulting crude **S6** was dissolved in dry CH_2Cl_2 (30 mL), to which was added Dess-Martin periodinane (665 mg, 1.57 mmol, 2.0 eq.). After stirring for 1 h, the reaction was quenched with saturated solutions of $NaHCO_3$ (10 mL) and $Na_2S_2O_3$ (10 mL), and extracted with CH_2Cl_2 (3 × 10 mL). The combined organic layers were washed by brine, dried and filtered. After removal of the solvent, the residue was purified via flash column chromatography on silica gel (eluting with a gradient of petroleum ether /ethyl acetate = 30:1 to 10:1) to yield **30a** (77.6 mg, 35%) and **30b** (90.5 mg, 41%), both in the form of white powder.

Compounds S6: TLC (petroleum ether / ethyl acetate, 4:1 v/v): R_f = 0.27; ^{1}H NMR (400 MHz, $CDCl_3$): δ 9.89 (d, *J* = 2.0 Hz, 1H), 9.82 (d, *J* = 4.0 Hz, 1H), 5.70 (d, *J* = 3.6 Hz, 1H), 5.56 (d, *J* = 2.0 Hz, 1H), 5.50 (d, *J* = 3.2 Hz, 2H), 5.47 – 5.46 (m, 2H), 5.04 (s, 1H), 4.70 (d, *J* = 6.8 Hz, 6H), 4.56 (d, *J* = 5.2 Hz, 6H), 3.92 (d, *J* = 4.8 Hz, 1H), 3.74 (t, *J* = 6.4 Hz, 1H), 3.63 (d, *J* = 4.8 Hz, 2H), 3.57 (s, 1H), 3.48 (s, 2H), 2.48 (s, 1H), 2.41 – 2.35 (m, 2H), 2.28 (d, *J* = 8.8 Hz, 1H), 2.25 – 2.19 (m, 3H), 2.18 – 2.13 (m, 6H), 2.10 – 1.96 (m, 26), 1.96 – 1.89 (m, 6H), 1.89 – 1.82 (m, 2H), 1.81 – 1.71 (m, 2H), 1.71 – 1.54 (m, 7H), 1.52 – 1.41(m, 5H), 1.33 – 1.19 (m, 10H), 1.12 (t, *J* = 12.4 Hz, 1H); ^{13}C NMR (100 MHz, $CDCl_3$): δ 207.4, 205.4, 151.7, 151.6, 151.4, 151.3, 147.4, 147.3, 146.5, 145.6, 121.4, 121.1, 118.3, 118.2, 105.6, 104.7, 104.2, 100.3, 89.2, 87.5, 84.3, 54.2, 53.8, 49.3, 46.7, 45.6, 44.7, 44.3, 44.1, 43.8, 43.6, 39.9, 39.4, 38.9, 38.8, 38.6, 37.4, 37.2, 37.0, 36.9, 36.6, 36.4, 36.1, 35.6, 35.5, 35.4, 34.9, 34.7, 32.9, 32.2, 31.2, 31.1, 30.4, 29.8, 27.7, 27.5, 25.7, 25.0, 24.2, 24.0, 23.9, 23.8, 21.0, 20.7, 18.9.

Compound 30a/b (upper): TLC (petroleum ether / ethyl acetate, 6:1 v/v): R_f = 0.73; ^{1}H NMR (400 MHz, $CDCl_3$): δ 9.77 (s, 1H), 5.52 (d, *J* = 4.4 Hz, 1H), 4.79 (s, 1H), 4.66 (s, 1H), 3.28 (d, *J* = 11.2 Hz, 1H), 2.32 – 2.27 (m, 4H), 2.22 – 2.18 (m, 1H), 2.14 – 2.02 (m, 3H), 2.00 – 1.94 (m, 3H), 1.84 – 1.78 (m, 1H), 1.72 – 1.64 (m, 1H), 1.59 – 1.54 (m, 3H), 1.52 – 1.47 (m, 1H), 1.20 (dd, *J* = 13.2, 5.2 Hz, 1H); ^{13}C NMR (100 MHz, $CDCl_3$): δ 217.4, 203.9, 149.4, 143.2, 122.5, 106.2, 51.3, 47.8, 47.1, 40.7, 38.2, 36.7, 36.5, 34.4, 31.0, 29.3, 25.4, 23.8, 17.7; IR (neat): ν_{max} = 2930, 2864, 1776, 1713, 1653, 1461, 1254, 1118, 1032, 908, 873, 734 cm^{-1}; HRMS (*m/z*): $[M+H]^+$ calcd. for $C_{19}H_{23}O_2$, 283.1698; found, 283.1690.

Compound 30a/b (lower): TLC (petroleum ether / ethyl acetate, 6:1 v/v): R_f = 0.51; ^{1}H NMR (400 MHz, $CDCl_3$): δ 9.83 (s, 1H), 5.68 (d, *J* = 1.2 Hz, 1H), 4.78 (s, 1H), 4.64 (s, 1H), 2.45 (d, *J* = 9.6 Hz, 1H), 2.30 – 2.22 (m, 4H), 2.17 – 1.98 (m, 7H), 1.87 – 1.79 (m, 1H), 1.70 – 1.65 (m, 2H), 1.60 – 1.59 (m, 1H), 1.56 – 1.51 (m, 2H); ^{13}C NMR (100 MHz, $CDCl_3$): δ 215.9, 203.4, 149.4, 143.9, 124.0, 106.0, 54.1, 50.1, 47.9, 38.8, 38.1, 37.5, 36.5, 34.6, 31.3, 30.3, 29.7, 23.7, 21.1; IR (neat): ν_{max} = 3339, 2926, 2863, 1718, 1652, 1448, 1258, 1038, 1014, 870, 806, 524 cm^{-1}; HRMS (*m/z*): $[M+H]^+$ calcd. for $C_{19}H_{23}O_2$, 283.1698; found, 283.1692.

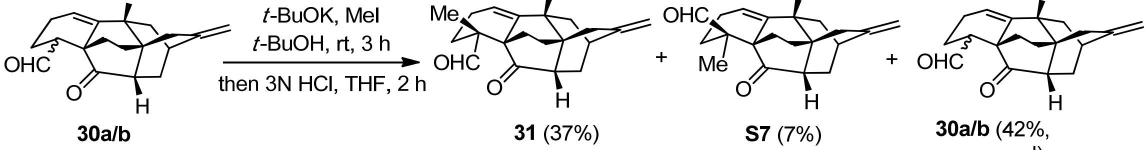

30a/b **31** (37%) **S7** (7%) **30a/b** (42%, recovered)

To a mixture of **30a**/**30b** (65.0 mg, 0.230 mmol, 1.0 eq.) in *t*-BuOH (10 mL) was added *t*-BuOK (1.0 M in *t*-BuOH, 922 μL, 0.922 mmol, 4.0 eq.) followed by addition of iodomethane (143 μL, 2.30 mmol,

10.0 eq.) 10 minutes later. The reaction was allowed to stir at room temperature for 3 h until TLC analysis indicated consumption of the starting material. A solution of HCl (3.0 M in water, 7.0 mL) and 3.0 mL THF were added to the reaction at the same temperature. After stirring for another 2 h, the reaction was quenched with saturated $NaHCO_3$ (10 mL) solution and extracted with EtOAc (3 × 15 mL). The combined organic layers were washed with saturated $NaHCO_3$ solution, filtered over anhydrous $MgSO_4$, and concentrated under reduced pressure. The crude residue was purified by column chromatography on silica gel (eluting with a gradient of petroleum ether / ethyl acetate = 30:1 to 10:1) to afford the compounds **31** (25.4 mg, 37%) and **S7** (4.9 mg, 7%), together with the recovered **30a/b** (27.1 mg, 42% recycling yield).

Compound 31: TLC (petroleum ether / ethyl acetate, 10:1 v/v): $R_f = 0.37$; ^{1}H NMR (400 MHz, $CDCl_3$): δ 9.66 (s, 1H), 5.56 (s, 1H), 4.80 (s, 1H), 4.67 (s, 1H), 2.37 – 2.32 (m, 1H), 2.28 – 2.24 (m, 3H), 2.22 – 2.19 (m, 1H), 2.08 – 1.89 (m, 5H), 1.88 – 1.77 (m, 2H), 1.69 – 1.54 (m, 4H), 1.23 (dd, *J* = 13.2, 5.6 Hz, 1H), 1.02 (s, 3H); ^{13}C NMR (100 MHz, $CDCl_3$): δ 217.0, 205.7, 149.3, 142.9, 121.6, 106.5, 54.9, 47.1, 45.2, 41.2, 38.2, 36.4, 35.9, 34.4, 31.1, 30.2, 29.2, 26.5, 21.9, 17.4; IR (neat): ν_{max} = 2935, 2859, 1714, 1655, 1461, 1368, 1089, 912, 877, 683 cm^{-1}; HRMS (*m/z*): $[M+H]^+$ calcd. for $C_{20}H_{25}O_2$, 297.1855; found, 297.1850.

Compound S7: TLC (petroleum ether / ethyl acetate, 10:1 v/v): $R_f = 0.42$; ^{1}H NMR (600 MHz, $CDCl_3$): δ 9.90 (s, 1H), 5.46 (s, 1H), 4.80 (s, 1H), 4.67 (s, 1H), 2.32 (br s, 1H), 2.26 (s, 1H), 2.22 – 2.18 (m, 3H), 2.08 – 2.05 (m, 2H), 2.03 – 1.99 (m, 1H), 1.98 – 1.94 (m, 2H), 1.88 – 1.85 (m, 1H), 1.83 – 1.78 (m, 1H), 1.73 – 1.68 (m, 1H), 1.58 – 1.52 (m, 2H), 1.51 – 1.46 (m, 1H), 1.43 (s, 3H), 1.22 (br s, 1H); ^{13}C NMR (150 MHz, $CDCl_3$): δ 216.1, 206.5, 149.2, 142.0, 120.9, 106.7, 55.2, 47.7, 47.4, 41.4, 38.3, 36.2, 35.3, 34.5, 31.5, 29.3, 29.0, 26.2, 21.0, 16.7; IR (neat): ν_{max} = 2973, 2930, 2857, 2728, 1713, 1655, 1460, 868 cm^{-1}; HRMS (*m/z*): $[M+H]^+$ calcd. for $C_{20}H_{25}O_2$, 297.1855; found, 297.1849.

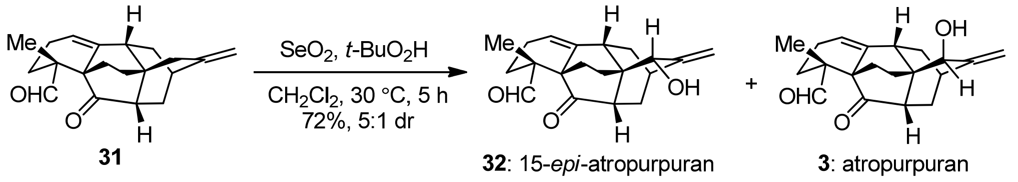

Procedure A (using an old SeO_2 stored in our laboratory): To a solution of **31** (19.2 mg, 0.060 mmol, 1.0 eq.) in CH_2Cl_2 (10 mL) was added SeO_2 (21.6 mg, 0.195 mmol, 3.0 eq.) followed by *t*-BuO_2H (70% in water, 265 μL, 1.95 mmol, 30.0 eq.). The mixture was stirred at 30 °C for 5 h before more SeO_2 (21.6 mg, 0.195 mmol, 3.0 eq.) was added. Another 5 h later, the reaction was quenched by saturated NH_4Cl solution (6 mL) and extracted with CH_2Cl_2 (3 × 5 mL). After the organic layers were washed with saturated $Na_2S_2O_3$ solution (5 mL), dried over anhydrous $MgSO_4$, filtered and evaporated to dryness, purification of the residue by flash column chromatography (petroleum ether / acetone = 8:1) gave an inseparable mixture of 15-*epi*-atropurpuran **32** and atropurpuran **3** (9.2 mg, 45%, **32** : **3** = 14: 1) as a white powder.

Procedure B (using a freshly ordered SeO_2): To a solution of **31** (20.5 mg, 0.069 mmol, 1.0 eq.) in CH_2Cl_2 (10 mL) was added SeO_2 (23.0 mg, 0.207 mmol, 3.0 eq.) followed by *t*-BuO_2H (70% in water, 267 μL, 2.070 mmol, 30.0 eq.), and the reaction was stirred at 30 °C for 5 h. Upon cooling, the reaction was quenched by saturated NH_4Cl solution (6 mL) and extracted with CH_2Cl_2 (3 × 5 mL). Usual work-up as described above gave a crude residue, which was purified by flash column chromatography

(petroleum ether / acetone = 8:1) to afford an inseparable mixture of 15-*epi*-atropurpuran **32** and atropurpuran **3** (15.6 mg, 72%, **32** : **3** = 5: 1) as a white powder. Pure 15-*epi*-atropurpuran **32** was obtained by further separation through a silica gel column chromatography (dichloromethane / petroleum ether / ethyl acetate = 5:5:1).

Compound 32: TLC (dichloromethane / petroleum ether / ethyl acetate, 3:3:1 v/v/v): R_f = 0.20; ^{1}H NMR (400 MHz, $CDCl_3$): δ9.66 (s, 1H), 5.57 (s, 1H), 5.08 (s, 1H), 5.05 (s, 1H), 4.02 (s, 1H), 2.57 (d, *J* = 11.2 Hz, 1H), 2.37 (s, 1H), 2.29 – 2.21 (m, 1H), 2.18 – 2.06 (m, 2H), 2.04 – 1.90 (m,4H), 1.88 – 1.75 (m, 2H), 1.70 – 1.62 (m, 1H), 1.59 – 1.48 (m, 3H), 1.22 – 1.17 (m, 1H), 1.03 (s, 3H); ^{13}C NMR (100 MHz, $CDCl_3$): δ 217.4, 205.6, 154.0, 142.3, 121.9, 111.3, 73.1, 54.9, 45.2, 41.8, 39.1, 38.7, 35.7, 35.1, 31.1, 30.3, 26.2, 25.2, 21.9, 17.4; IR (neat): ν_{max} = 3427, 2925, 2862, 1710, 1668, 1461, 1262, 1041, 1018, 900, 808, 724 cm^{-1}; HRMS (*m/z*): $[M+H]^+$ calcd. for $C_{20}H_{25}O_3$, 313.1804; found, 313.1798.

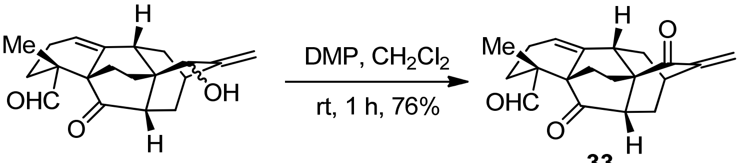

32: **3** = 5: 1 **33**

To a solution of a mixture of **32** and **3** (5:1, 12.3 mg, 0.039 mmol, 1.0 eq.) in CH_2Cl_2 (2 mL) was added Dess-Martin periodinane (49.9 mg, 0.117 mmol, 3.0 eq.). The suspension kept stirring at room temperature for 1 h before it was quenched with saturated $NaHCO_3$ solution (2 mL) and saturated $Na_2S_2O_3$ solution (2 mL), then extracted with CH_2Cl_2 (3 × 5 mL). The organic extracts were combined, filtered over anhydrous $MgSO_4$, and concentrated under vacuum. The resulting crude residue was subjected to silica gel column chromatography (petroleum ether / ethyl acetate = 3:1) to afford enone **33** (9.2 mg, 76%) as a white powder.

Compound 33: TLC (petroleum ether / acetone, 4:1 v/v): R_f = 0.52; ^{1}H NMR (400 MHz, $CDCl_3$): δ 9.68 (s, 1H), 6.02 (s, 1H), 5.64 (s, 1H), 5.26 (s, 1H), 2.80 (s, 1H), 2.68 – 2.64 (m, 1H), 2.45 (d, *J* = 12.0 Hz, 1H), 2.25 – 2.20 (m, 2H), 2.17 – 2.09 (m, 1H), 2.04 – 1.97 (m, 2H), 1.94 – 1.89 (m, 1H), 1.88 – 1.80 (m, 2H), 1.67 – 1.61 (m, 2H), 1.49 – 1.42 (m, 1H), 1.28 – 1.21 (m, 1H), 1.06 (s, 3H); ^{13}C NMR (100 MHz, $CDCl_3$): δ 214.6, 205.0, 200.2, 146.5, 141.0, 123.4, 117.8, 54.7, 46.6, 45.3, 44.6, 38.8, 36.1, 34.6, 31.9, 29.8, 25.8, 21.9, 21.6, 17.5; IR (neat): ν_{max} = 2926, 2808, 1710, 1633, 1460, 1176, 1048, 941 cm^{-1}; HRMS (*m/z*): $[M+H]^+$ calcd. for $C_{20}H_{23}O_3$, 311.1647; found, 311.1642.

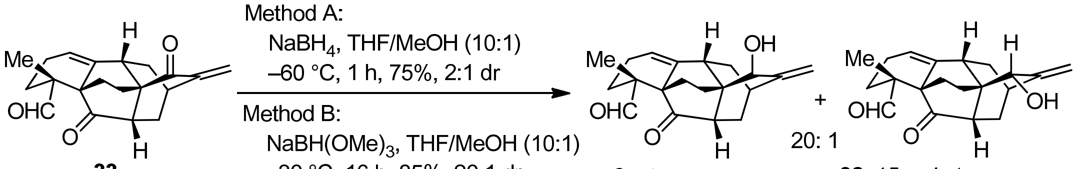

33 **3**: atropurpuran **32**: 15-*epi*-atropurpuran

Method A: A solution of ketone **33** (4.2 mg, 0.0135 mmol, 1.0 eq.) in THF/MeOH (10:1, 1.1 mL) was cooled to –60 °C, to which was added $NaBH_4$ (2.1 mg, 0.054 mmol, 4.0 eq.). The reaction was stirred at the same temperature for 1 h before being quenched with saturated NH_4Cl solution. After warming to room temperature, the mixture was extracted with EtOAc (3 × 3 mL). The organic extracts were combined, washed with brine, dried with anhydrous $MgSO_4$, filtered through a silica gel pad, and

concentrated under vacuum. The so obtained crude product (3.2 mg, 75%) was subjected to the proton NMR experiment: the ratio of compounds **3** to **32** was observed to be 2:1.

Method B: To a solution of **33** (5.3 mg, 0.017 mmol, 1.0 eq.) in THF/MeOH (10:1, 1.1 mL) was added $NaBH(OMe)_3$ (13.1 mg, 0.102 mmol, 6.0 eq.) at –30 °C, and the mixture was kept stirring at the same temperature for 16 h. After being quenched by addition of saturated NH_4Cl solution, the same work-up procedure as above described was carried out to afford a crude product. Purification by silica gel column chromatography (petroleum ether / acetone = 8:1) gave an inseparable mixture of atropurpuran **3** and 15-*epi*-atropurpuran **32** (4.6 mg, 85%, **3**: **32** = 20: 1) as a white powder. Pure atropurpuran **3** was obtained by further separation through a silica gel column chromatography (dichloromethane / petroleum ether / ethyl acetate = 5:5:1).

Atropurpuran 3: TLC (dichloromethane / petroleum ether / ethyl acetate, 3:3:1 v/v/v): R_f = 0.27; ^1H NMR (400 MHz, $CDCl_3$): δ 9.66 (s, 1H), 5.60 (s, 1H), 5.15 (s, 1H), 5.05 (s, 1H), 4.14 (s, 1H), 2.74 – 2.70 (m, 1H), 2.37 (s, 1H), 2.21 (d, J = 11.6 Hz, 1H), 2.10 – 1.96 (m, 5H), 1.92 – 1.81 (m, 2H), 1.70 – 1.55 (m, 4H), 1.23 – 1.19 (m, 1H), 1.03 (s, 3H); ^{13}C NMR (150 MHz, $CDCl_3$): δ 215.7, 205.3, 154.2, 142.3, 122.3, 108.7, 72.7, 54.9, 45.9, 45.2, 39.1, 36.0, 36.0, 34.0, 30.1, 29.9, 26.1, 25.2, 21.8, 17.3; IR (neat): ν_{max} = 3419, 2938, 2866, 1712, 1461, 1396, 1070, 1024, 999, 733 cm^{-1}; HRMS (*m/z*): $[M+H]^+$ calcd. for $C_{20}H_{25}O_3$, 313.1804; found, 313.1798.

X-ray Crystallographic Data

A single crystal of **27** was obtained by recrystallization from petroleum ether/diethyl ether (5:1). Crystallographic data for **27** ($C_{25}H_{40}O_5Si$, mp 171 – 172 °C) have been deposited in the Cambridge Crystallographic Data Centre (CCDC 1435700). These data can be obtained free of charge via www.ccdc.cam.ac.uk/data_request/cif.

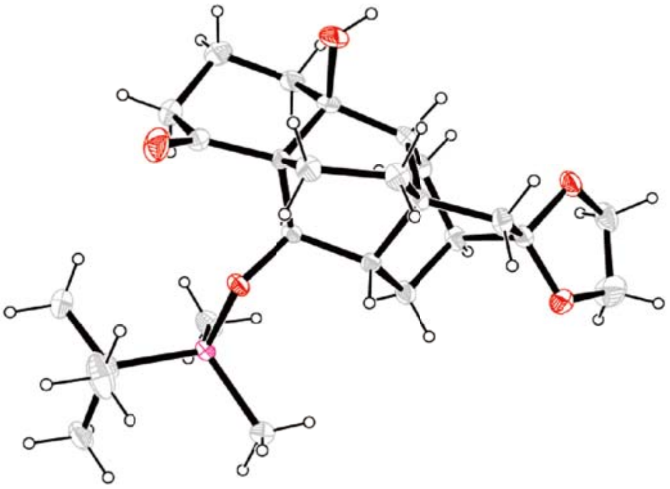

Crystal data and structure refinement for CCDC 1435700.

Identification code	151028_s1_qy_m
Empirical formula	$C_{25}H_{40}O_5Si$
Formula weight	448.66
Temperature/K	293.15
Crystal system	orthorhombic
Space group	Pbca
a/Å	11.5577(5)
b/Å	16.8408(7)
c/Å	24.6521(9)
α/°	90.00
β/°	90.00
γ/°	90.00
Volume/Å^3	4798.3(3)
Z	8
ρ_{calc}g/cm^3	1.242
μ/mm^{-1}	0.131
F(000)	1952.0
Crystal size/mm^3	0.3 × 0.2 × 0.2
Radiation	MoKα (λ = 0.71073)
2Θ range for data collection/°	5.86 to 52.74
Index ranges	$-14 \le h \le 8, -11 \le k \le 21, -21 \le l \le 30$
Reflections collected	14064

Independent reflections	4898 [R_{int} = 0.0247, R_{sigma} = 0.0333]
Data/restraints/parameters	4898/0/286
Goodness-of-fit on F^2	1.037
Final R indexes [I>=2σ (I)]	R_1 = 0.0479, wR_2 = 0.1143
Final R indexes [all data]	R_1 = 0.0699, wR_2 = 0.1270
Largest diff. peak/hole / e Å^{-3}	0.35/–0.23

Fractional Atomic Coordinates (×10^4) and Equivalent Isotropic Displacement Parameters (Å^2×10^3) U_{eq} is defined as 1/3 of of the trace of the orthogonalised U_{IJ} tensor.

Atom	*x*	*y*	*z*	U(eq)
Si1	4807.0(5)	4392.2(3)	6150.4(2)	36.38(16)
O1	5270.4(14)	880.5(9)	4236.0(6)	56.2(4)
O2	4158.0(15)	1934.3(10)	4016.5(6)	64.7(5)
O3	5627.6(17)	706.2(9)	6466.9(6)	61.7(4)
H3	5819	323	6283	93
O4	4739.3(16)	2440.3(11)	7341.4(6)	71.6(5)
O5	4547.2(12)	3446.2(8)	6271.0(5)	39.4(3)
C1	5613(3)	1101.9(16)	3702.9(9)	68.9(7)
H1A	5609	647	3461	83
H1B	6381	1335	3704	83
C2	4723(3)	1698(2)	3537.3(10)	100.0(11)
H2A	5087	2150	3364	120
H2B	4176	1463	3286	120
C3	4746.2(19)	1574.9(12)	4461.3(8)	45.8(5)
C4	5652.8(19)	2132.9(12)	4701.1(8)	45.4(5)
H4	6165	2327	4414	55
C5	5015(2)	2833.0(12)	4964.6(8)	47.9(5)
H5A	4443	3044	4715	58
H5B	5560	3253	5051	58
C6	4415.0(16)	2540.2(11)	5488.4(7)	35.0(4)
H6	3634	2766	5502	42
C7	4311.4(16)	1627.2(11)	5464.2(7)	36.6(4)
C8	3895.4(19)	1352.5(13)	4903.4(8)	47.5(5)
H8A	3793	781	4907	57
H8B	3151	1593	4826	57
C9	6357.0(18)	1698.2(13)	5130.2(8)	47.7(5)
H9A	6878	2069	5306	57
H9B	6820	1289	4958	57
C10	5550.9(17)	1312.2(11)	5563.5(8)	39.2(4)
H10	5540	739	5494	47

C11	3490.7(18)	1346.7(13)	5906.6(8)	47.2(5)
H11A	3546	775	5945	57
H11B	2701	1478	5808	57
C12	5875.5(18)	1430.2(11)	6173.4(8)	41.7(5)
C13	7149.9(19)	1630.3(14)	6275.4(9)	52.6(6)
H13A	7626	1193	6150	63
H13B	7355	2097	6066	63
C14	5043.9(16)	2065.5.3(11)	6418.9(7)	37.0(4)
C15	5061.6(16)	2773.7(10)	6016.3(7)	32.5(4)
H15	5867	2900	5927	39
C16	3800.0(19)	1746.2(13)	6446.2(8)	47.4(5)
H16A	3734	1365	6740	57
H16B	3268	2179	6517	57
C17	7409(2)	1785.1(16)	6871(1)	66.3(7)
H17A	8228	1893	6918	80
H17B	7214	1320	7085	80
C18	6701(2)	2494.5(15)	7062.5(9)	62.7(6)
H18A	6853	2590	7444	75
H18B	6932	2964	6862	75
C19	5433(2)	2348.7(13)	6980.8(8)	48.5(5)
C22	5453(3)	4752.5(18)	7217.3(10)	80.7(9)
H22A	5476	4192	7287	121
H22B	5292	5031	7549	121
H22C	6187	4923	7077	121
C23	4506.1(19)	4931.6(13)	6803.1(8)	47.2(5)
C24	3330(3)	4680.9(19)	7030.9(13)	91.2(10)
H24A	2734	4814	6776	137
H24B	3191	4953	7367	137
H24C	3328	4118	7093	137
C25	4484(2)	5828.7(15)	6697.2(11)	69.6(7)
H25A	5205	5989	6538	104
H25B	4373	6105	7034	104
H25C	3861	5953	6454	104
C26	3828(2)	4737.5(13)	5597.5(9)	58.8(6)
H26A	3978	4437	5274	88
H26B	3964	5291	5528	88
H26C	3037	4662	5706	88
C27	6338(2)	4534.3(14)	5932.8(11)	60.4(6)
H27A	6847	4320	6203	91

H27B	6491	5091	5888	91
H27C	6464	4265	5595	91

Anisotropic Displacement Parameters (Å^2×10^3). The Anisotropic displacement factor exponent takes the form: -2π^2[h^{2}a*^{2}U$_{11}$+2hka*b*U$_{12}$+…].

Atom	U_{11}	U_{22}	U_{33}	U_{23}	U_{13}	U_{12}
Si1	40.9(3)	29.5(3)	38.8(3)	–5.2(2)	0.9(2)	2.9(2)
O1	78.6(11)	42.9(9)	47.1(8)	–10.4(6)	7.8(8)	5.7(8)
O2	84.5(12)	63.0(11)	46.6(8)	–1.0(7)	–7.0(8)	15.7(9)
O3	90.7(13)	31.9(8)	62.6(10)	13.4(7)	–0.2(9)	1.9(8)
O4	90.0(13)	87.1(13)	37.7(8)	–3.7(8)	12.3(8)	–2.9(10)
O5	46.9(8)	31.1(7)	40.1(7)	–4.0(5)	8.4(6)	4.7(6)
C1	96(2)	61.2(16)	49.7(13)	–16.8(11)	15.9(13)	–0.5(15)
C2	135(3)	123(3)	42.3(14)	3.5(16)	9.1(16)	31(2)
C3	60.3(13)	37.2(11)	39.9(10)	–5.7(9)	0.5(10)	3.9(10)
C4	56.2(12)	42.1(12)	38(1)	–2.9(8)	13.8(9)	–6.8(10)
C5	72.5(15)	34.2(11)	37.1(10)	0.2(8)	3.7(10)	–4.9(10)
C6	37(1)	32.9(10)	35.1(9)	0.1(7)	2.2(8)	4.0(8)
C7	35.4(10)	32.3(10)	42.1(10)	–2.3(8)	3.2(8)	–2.6(8)
C8	47.4(12)	44.5(12)	50.5(11)	–9.6(9)	–1.3(10)	–6.7(10)
C9	41.7(11)	52.3(13)	48.9(11)	–10.3(10)	12.0(9)	–2.1(10)
C10	42.0(11)	26.8(9)	48.7(11)	–4.7(8)	5.2(9)	1.1(8)
C11	42.1(11)	45.6(12)	54.0(12)	1.4(9)	7.8(10)	–10.9(10)
C12	48.6(12)	27.3(10)	49.2(11)	7.6(8)	0.9(9)	3.2(9)
C13	45.4(12)	47.5(13)	64.9(13)	–2.8(10)	–9.1(10)	15(1)
C14	43.2(11)	33.2(10)	34.5(9)	3.6(8)	4.0(8)	–0.3(9)
C15	35.7(9)	27.4(9)	34.5(9)	0.1(7)	7.1(7)	2.9(8)
C16	50.8(12)	45.4(12)	46.1(11)	4.4(9)	12.9(10)	–7(1)
C17	62.8(15)	67.6(16)	68.5(15)	3.8(12)	–21.0(13)	11.7(13)
C18	71.2(16)	65.6(16)	51.3(13)	–6.2(11)	–16.8(12)	4.1(13)
C19	66.1(14)	40.9(12)	38.7(11)	5.7(9)	–2.4(10)	3.7(10)
C22	104(2)	85(2)	52.5(14)	–20.4(14)	–23.2(15)	19.6(17)
C23	50.1(12)	43.8(12)	47.6(11)	–17.8(9)	–0.7(10)	2.7(10)
C24	83(2)	100(2)	90(2)	–47.6(17)	42.5(17)	–20.5(17)
C25	78.8(18)	48.9(14)	81.2(18)	–29.4(13)	–9.8(14)	7.3(13)
C26	76.8(17)	47.4(13)	52.0(12)	–0.6(10)	-9.2(12)	12.6(12)
C27	55.3(14)	47.9(14)	77.9(16)	–7.7(11)	16.3(12)	–7.2(11)

Bond Lengths.

Atom	Atom	Length/Å	Atom	Atom	Length/Å
Si1	O5	1.6482(14)	C15	C14	1.552(2)

Si1	C27	1.864(2)	C4	C3	1.527(3)
Si1	C23	1.880(2)	C4	C5	1.535(3)
Si1	C26	1.865(2)	C4	C9	1.522(3)
O5	C15	1.425(2)	C6	C5	1.547(3)
O1	C3	1.429(2)	C3	C8	1.515(3)
O1	C1	1.423(3)	C14	C12	1.560(3)
O2	C3	1.425(3)	C14	C19	1.532(3)
O2	C2	1.407(3)	C14	C16	1.536(3)
O3	C12	1.447(2)	C12	C13	1.532(3)
O4	C19	1.207(3)	C19	C18	1.500(3)
C7	C10	1.547(3)	C16	C11	1.533(3)
C7	C6	1.543(3)	C13	C17	1.521(3)
C7	C11	1.521(3)	C17	C18	1.523(3)
C7	C8	1.535(3)	C1	C2	1.494(4)
C10	C12	1.562(3)	C23	C25	1.533(3)
C10	C9	1.559(3)	C23	C22	1.527(3)
C15	C6	1.551(3)	C23	C24	1.530(3)

Bond Angles.

Atom	Atom	Atom	Angle/°	Atom	Atom	Atom	Angle/°
O5	Si1	C27	110.42(9)	C8	C3	C4	108.61(16)
O5	Si1	C23	106.19(9)	C15	C14	C12	105.71(14)
O5	Si1	C26	108.83(9)	C19	C14	C15	109.58(15)
C27	Si1	C23	111.09(10)	C19	C14	C12	112.55(16)
C27	Si1	C26	109.01(12)	C19	C14	C16	110.10(16)
C26	Si1	C23	111.26(10)	C16	C14	C15	108.02(15)
C15	O5	Si1	127.79(11)	C16	C14	C12	110.70(16)
C1	O1	C3	105.23(17)	O3	C12	C10	109.07(16)
C2	O2	C3	107.72(19)	O3	C12	C14	105.17(15)
C6	C7	C10	105.29(15)	O3	C12	C13	107.08(16)
C11	C7	C10	110.96(16)	C14	C12	C10	108.22(15)
C11	C7	C6	109.27(15)	C13	C12	C10	114.65(17)
C11	C7	C8	110.91(16)	C13	C12	C14	112.19(16)
C8	C7	C10	109.22(15)	O4	C19	C14	120.7(2)
C8	C7	C6	111.06(15)	O4	C19	C18	122.0(2)
C7	C10	C12	109.33(15)	C18	C19	C14	117.31(19)
C7	C10	C9	107.58(16)	C11	C16	C14	109.50(15)
C9	C10	C12	117.56(16)	C7	C11	C16	109.91(16)
O5	C15	C6	111.72(14)	C17	C13	C12	112.70(19)
O5	C15	C14	108.88(13)	C3	C8	C7	111.72(17)

C6	C15	C14	109.59(15)	C4	C5	C6	108.89(16)
C3	C4	C5	107.89(17)	C4	C9	C10	110.91(16)
C9	C4	C3	109.88(17)	C13	C17	C18	109.13(18)
C9	C4	C5	109.40(16)	O1	C1	C2	103.7(2)
C7	C6	C15	108.80(14)	C25	C23	Si1	109.47(16)
C7	C6	C5	108.67(14)	C22	C23	Si1	110.14(15)
C5	C6	C15	113.80(16)	C22	C23	C25	108.7(2)
O1	C3	C4	111.30(18)	C22	C23	C24	109.7(2)
O1	C3	C8	110.63(17)	C24	C23	Si1	110.19(15)
O2	C3	O1	104.52(15)	C24	C23	C25	108.6(2)
O2	C3	C4	111.34(17)	C19	C18	C17	110.8(2)
O2	C3	C8	110.43(18)	O2	C2	C1	106.3(2)

A	B	C	D	Angle/°	A	B	C	D	Angle/°
Si1	O5	C15	C6	–86.46(18)	C12	C14	C19	O4	–137.6(2)
Si1	O5	C15	C14	152.36(13)	C12	C14	C19	C18	41.6(2)
O5	Si1	C23	C25	–168.45(16)	C12	C14	C16	C11	–46.1(2)
O5	Si1	C23	C22	72.12(19)	C12	C13	C17	C18	–61.2(3)
O5	Si1	C23	C24	–49.0(2)	C19	C14	C12	O3	74.9(2)
O5	C15	C6	C7	–138.36(15)	C19	C14	C12	C10	–168.64(15)
O5	C15	C6	C5	100.32(18)	C19	C14	C12	C13	–41.2(2)
O5	C15	C14	C12	–166.39(15)	C19	C14	C16	C11	–171.15(17)
O5	C15	C14	C19	–44.9(2)	C16	C14	C12	O3	–48.8(2)
O5	C15	C14	C16	75.08(18)	C16	C14	C12	C10	67.66(19)
O1	C3	C8	C7	112.54(19)	C16	C14	C12	C13	–164.86(16)
O1	C1	C2	O2	15.9(3)	C16	C14	C19	O4	–13.6(3)
O2	C3	C8	C7	–132.24(18)	C16	C14	C19	C18	165.62(19)
O3	C12	C13	C17	–62.1(2)	C11	C7	C10	C12	–45.4(2)
O4	C19	C18	C17	128.8(2)	C11	C7	C10	C9	–174.06(15)
C7	C10	C12	O3	94.93(18)	C11	C7	C6	C15	68.41(19)
C7	C10	C12	C14	–19.0(2)	C11	C7	C6	C5	–167.18(16)
C7	C10	C12	C13	–145.04(17)	C11	C7	C8	C3	–175.90(17)
C7	C10	C9	C4	–9.7(2)	C13	C17	C18	C19	58.1(3)
C7	C6	C5	C4	–18.9(2)	C8	C7	C10	C12	–167.92(16)
C10	C7	C6	C15	–50.82(18)	C8	C7	C10	C9	63.37(19)
C10	C7	C6	C5	73.58(18)	C8	C7	C6	C15	–168.92(15)
C10	C7	C11	C16	68.3(2)	C8	C7	C6	C5	–44.5(2)
C10	C7	C8	C3	–53.3(2)	C8	C7	C11	C16	–170.11(17)
C10	C12	C13	C17	176.78(18)	C5	C4	C3	O1	–176.48(16)
C15	C6	C5	C4	102.53(19)	C5	C4	C3	O2	67.3(2)

C15	C14	C12	O3	–165.52(15)	C5	C4	C3	C8	–54.5(2)
C15	C14	C12	C10	–49.06(19)	C5	C4	C9	C10	64.8(2)
C15	C14	C12	C13	78.42(19)	C9	C10	C12	O3	–142.11(18)
C15	C14	C19	O4	105.1(2)	C9	C10	C12	C14	103.97(19)
C15	C14	C19	C18	–75.7(2)	C9	C10	C12	C13	–22.1(2)
C15	C14	C16	C11	69.2(2)	C9	C4	C3	O1	–57.3(2)
C4	C3	C8	C7	–9.9(2)	C9	C4	C3	O2	–173.46(16)
C6	C7	C10	C12	72.76(17)	C9	C4	C3	C8	64.7(2)
C6	C7	C10	C9	–55.96(18)	C9	C4	C5	C6	–47.6(2)
C6	C7	C11	C16	–47.4(2)	C27	Si1	O5	C15	–31.04(18)
C6	C7	C8	C3	62.4(2)	C27	Si1	C23	C25	71.45(19)
C6	C15	C14	C12	71.13(18)	C27	Si1	C23	C22	–48.0(2)
C6	C15	C14	C19	–167.34(15)	C27	Si1	C23	C24	–169.11(19)
C6	C15	C14	C16	–47.39(19)	C1	O1	C3	O2	36.1(2)
C3	O1	C1	C2	–31.7(3)	C1	O1	C3	C4	–84.2(2)
C3	O2	C2	C1	6.1(3)	C1	O1	C3	C8	154.9(2)
C3	C4	C5	C6	71.9(2)	C23	Si1	O5	C15	–151.57(15)
C3	C4	C9	C10	–53.5(2)	C26	Si1	O5	C15	88.57(16)
C14	C15	C6	C7	–17.60(19)	C26	Si1	C23	C25	–50.2(2)
C14	C15	C6	C5	–138.91(16)	C26	Si1	C23	C22	–169.62(18)
C14	C12	C13	C17	52.8(2)	C26	Si1	C23	C24	69.2(2)
C14	C19	C18	C17	–50.4(3)	C2	O2	C3	O1	–25.8(3)
C14	C16	C11	C7	–19.1(2)	C2	O2	C3	C4	94.5(2)
C12	C10	C9	C4	–133.57(17)	C2	O2	C3	C8	–144.7(2)
